# Impact of Methodological Assumptions and Covariates on the Cutoff Estimation in ROC Analysis

**DOI:** 10.1002/bimj.70053

**Published:** 2025-04-27

**Authors:** Soutik Ghosal

**Affiliations:** ^1^ Division of Biostatistics Department of Public Health Sciences School of Medicine University of Virginia Charlottesville Virginia USA

**Keywords:** Alzheimer's disease, AUC, cutoff estimation, diagnostic accuracy, ROC curve

## Abstract

The receiver operating characteristic (ROC) curve stands as a cornerstone in assessing the efficacy of biomarkers for disease diagnosis. Beyond merely evaluating performance, it provides with an optimal cutoff for biomarker values, crucial for disease categorization. While diverse methodologies exist for cutoff estimation, less attention has been paid to integrating covariate impact into this process. Covariates can strongly impact diagnostic summaries, leading to variations across different covariate levels. Therefore, a tailored covariate‐based framework is imperative for outlining covariate‐specific optimal cutoffs. Moreover, recent investigations into cutoff estimators have overlooked the influence of ROC curve estimation methodologies. This study endeavors to bridge this gap by addressing the research void. Extensive simulation studies are conducted to scrutinize the performance of ROC curve estimation models in estimating different cutoffs in varying scenarios, encompassing diverse data‐generating mechanisms and covariate effects. In addition, leveraging the Alzheimer's Disease Neuroimaging Initiative (ADNI) data set, the research assesses the performance of different biomarkers in diagnosing Alzheimer's disease and determines the suitable optimal cutoffs.

## 1 Introduction

The receiver operating characteristic (ROC) curve is a graphical tool for evaluating the diagnostic accuracy of biomarkers for detecting disease with binary outcomes, making it one of the most widely embraced tools in medical research. This graphical representation (Green and Swets 1966) is crafted by plotting sensitivities (true positive rates) against 1‐specificities–(false positive rates) across various biomarker cutoffs. Over decades, ROC curve analysis has been integral to biomarker assessment, with the area under the ROC curve (AUC) emerging as a pivotal metric for quantifying performance in disease discrimination. However, ROC curve analysis goes beyond mere summarization. It facilitates the identification of optimal biomarker cutoffs, enabling precise disease categorization for future biomarker assessments. This additional dimension of the ROC curve elevates its significance beyond its graphical representation, enhancing its practicality and relevance in clinical settings.

In the domain of optimal cutoff estimation, a multitude of frameworks have emerged in the literature over the past 70 years. Among these, the Youden Index (Youden 1950) reigns as the oldest and most widely recognized. This method identifies the optimal cutoff by maximizing the sum of sensitivity and specificity, offering a foundational approach to cutoff determination. Subsequently, Perkins and Schisterman (2006) introduced the closest‐to‐(0,1) criterion, which chooses the optimum cutoff by minimizing the distance of ROC curve points from the perfect classifier point (0,1), where both sensitivity and specificity are at their maximum. Building upon this, Liu (2012) proposed the concordance probability method, which seeks the optimal cutoff by maximizing the product of sensitivity and specificity, adding a nuanced perspective to cutoff selection. In the latest decade, Unal (2017) introduced the index of union approach, which derives an optimal cutoff by concurrently maximizing sensitivity and specificity values from the AUC value.

While these methodologies have been actively applied in the literature, their capacity to incorporate covariates remains largely unexplored. Biomarker performance seldom exhibits uniformity, and its performance may vary across distinct subpopulations characterized by specific covariates. Consequently, covariates hold the potential to significantly influence the diagnostic efficacy of biomarkers, prompting the need for methodological frameworks to appropriately accommodate them (de Carvalho et al. 2013; Ishwaran and Gatsonis 2000; Pepe 1997; Toledano and Gatsonis 1996; Tosteson and Begg 1988). It is logical to presume that if diagnostic summaries differ across various levels of covariates, optimal cutoffs will correspondingly vary. For example, in the literature, various cerebrospinal fluid biomarkers are recognized for screening Alzheimer's disease (AD), and their overall performance has been evaluated for this purpose. Research indicates significant differences in biomarker levels between sexes (Mielke 2020; Sundermann et al. 2020), suggesting potential variations in diagnostic capacity in different sex groups. Consequently, it is essential to employ a framework that facilitates the estimation of sex‐specific cutoffs. Covariates can also be incorporated within a probability index model (PIM) framework (De Schryver and De Neve 2019; Thas et al. 2012; Vermeulen et al. 2015), which estimates the probability index or the probability of superiority of biomarkers between groups, conditioned on covariates. This approach is relevant to the ROC framework since the covariate‐adjusted AUC can be expressed as the probability index or Mann–Whitney statistic. Brumback et al. (2006) extended this concept to the ROC context; however, direct cutoff estimation within the PIM framework remains challenging. So far in the literature, several works (Faraggi 2003; Hu et al. 2021; de Carvalho et al. 2017; Schisterman et al. 2008; Xu et al. 2014) have been done to propose covariate‐adjusted frameworks to estimate only the Youden's index. To et al. (2022) extended some of the aforementioned optimal cutoff estimators to accommodate covariates for the ROC surface. However, all of the aforementioned methodologies have yet to be formally extended in the context of the ROC curves to incorporate covariate considerations, leaving a notable gap in current research efforts.

Over the years, numerous classes of ROC curves have emerged across various methodological frameworks, spanning empirical (DeLong et al. 1988), parametric (Dorfman and Alf 1968; Metz et al. 1998), semiparametric (Pepe 2000), and nonparametric (Hsieh and Turnbull 1996; Lloyd 1998; Zou et al. 1997) domains. While the empirical ROC curve remains popular among researchers for its simplicity and lack of distributional assumptions, model‐based ROC curves offer distinct advantages, including the ability to generate smooth estimates. In recent years, an alternative modeling framework for ROC curves gained traction, centered around placement value (PV), a standardization of diseased biomarker scores relative to healthy biomarker distributions (Pepe 2003). PV‐based models have proven valuable due to their direct link to the ROC curve, as well as their capability to accommodate covariate effects (Alonzo and Pepe 2002; Cai 2004; Pepe and Cai 2004; Stanley and Tubbs 2018) and constraints (Ghosal and Chen 2022; Ghosal et al. 2022). In addition, the literature has seen the emergence of shape‐constrained ROC models, ensuring strictly concave ROC curves to avoid “improper” curves with hooks at extreme specificity levels. Notable examples include bibeta (Mossman and Peng 2016), bigamma (Dorfman et al. 1996), and bichi‐squared (Hillis 2016) ROC curves. Gonçalves et al. (2014) have aptly summarized many of these methodologies. It is important to note that methodological assumptions of ROC curve modeling could play a significant role in optimal cutoff estimation. Given that the ROC curve is dependent on the distributions of healthy and diseased biomarkers, estimating cutoffs involves optimizing the functions involving sensitivity and specificity, both of which are functions of these distributions. Any variation in these distributions within the ROC framework can naturally influence the estimation of cutoff points. However, the comparative evaluation of different cutoff estimators has overlooked the impact of various ROC estimation models (Hajian‐Tilaki 2018; Rota and Antolini 2014; Unal 2017), creating a gap in research.

The novelty of our work lies in addressing these gaps in diagnostic accuracy research. Primarily, we aim to shed light on the performance of optimal cutoff methods across various ROC estimating models. When selecting a cutoff point, it is crucial to recognize that different criteria may lead to distinct optimal values (details of which will be illustrated later). This study aims to investigate how various methodological assumptions influence the estimation of these different cutoffs. By examining the robustness of these cutoff frameworks under diverse conditions, we can gain valuable insights into their reliability and applicability in different contexts. In addition, we seek to enhance current optimal cutoff frameworks by integrating covariate considerations. This comprehensive approach promises to provide valuable insights into both cutoff estimation methodologies and the impact of covariates on diagnostic accuracy assessment. The rest of the article is organized as follows. Section 2 provides detailed descriptions of different ROC curve models, optimal cutoff frameworks, covariate adjustment, and estimation mechanisms. We demonstrate the performance of the methodology through extensive simulation in Section 3 and present application with the AD data in Section 4. We conclude with a brief discussion in Section 5.

## 2 Methodology

### 2.1 General Framework

Let $Y_{0}$ and $Y_{1}$ be the continuous distributions of the biomarkers for the healthy and diseased subjects, respectively, discriminating the disease D of binary classification (D=0 or D=1, respectively, denoting whether the corresponding group is healthy or diseased). Conventionally, we assume that

$$Y_{0}∼F_{0}\left(\cdot \right)\;\text{and}\;Y_{1}∼F_{1}\left(\cdot \right),$$

where $F_{0}$ and $F_{1}$ denote the continuous distribution functions of healthy and diseased biomarkers, respectively. Then, the corresponding ROC curve and its summary AUC can be written as

(1) $$\begin{array}{cc} ROC(t) & =1-F_{1}\left(F_{0}^{-1}\left(1-t\right)\right),\;t\in \left(0,1\right), \end{array}$$

(2) $$\begin{array}{cc} AUC & =\int_{0}^{1}ROC\left(t\right)dt. \end{array}$$

Without loss of generality, let us assume that higher values of the biomarker indicate a diseased group, that is, $Y_{0}<Y_{1}$. Based on the distributional assumption of $Y_{0}$ and $Y_{1}$, we can write the sensitivity (se) and specificity (sp) at a certain cutoff c as

(3) $$\begin{array}{cc} se(c) & =P\left[Y_{1}>c\right]=1-F_{1}\left(c\right),\;\text{and}\\ sp(c) & =P\left[Y_{0}<c\right]=F_{0}\left(c\right). \end{array}$$

#### 2.1.1 Special ROC Frameworks

Drawing from the general definition of the ROC curve in (1), various types of ROC frameworks have emerged in the literature, including empirical, parametric, and nonparametric approaches. In this study, we will compare several of these ROC models, including the empirical (Emp) model, the Kernel‐based nonparametric model (NonPar), and the widely used binormal (BN) model. For a comprehensive overview of these frameworks and others, please refer to Section A.1 in the Appendix.

#### 2.1.2 Optimal Cutoffs

In this section, we introduce the mathematical definitions of the most renowned optimal cutoff methods employed in diagnostic accuracy research. Unal (2017) provided a thorough summary of various cutoff estimators. However, for the sake of completeness, we will provide a brief overview of them here as well.

1. **Youden's index** (J): Youden's index (Youden 1950) stands out as one of the oldest and most widely used optimal cutoff frameworks. It determines the cutoff by maximizing the sum of sensitivities and specificities across various cutoff points.
(4) $$\begin{array}{cc} J(c) & =se(c)+sp(c)-1,\\ c_{J} & =\underset{c\in ℜ}{arg max}J\left(c\right). \end{array}$$
2. **Closest to** (0,1) **criteria** (ER): In this criterion (Perkins and Schisterman 2006), the optimal cutoff is derived by minimizing the Euclidean distance between the ROC curve and the perfect classifier point, which is located at coordinates (0,1). The optimal cutoff is thus determined as the cutoff for which the sensitivity and 1‐ specificity pair on the ROC curve is closest to (0,1).
(5) $$\begin{array}{cc} ER(c) & =\sqrt{{\left(1-se\left(c\right)\right)}^{2}+{\left(1-sp\left(c\right)\right)}^{2}},\\ c_{ER} & =\underset{c\in ℜ}{arg min}ER\left(c\right). \end{array}$$
3. **Concordance probability method criteria** (CZ): This criterion (Liu 2012) operates by maximizing the product of sensitivity and specificity across different cutoffs. The optimal cutoff is identified as the cutoff that achieves the maximum product.
(6) $$\begin{array}{cc} CZ(c) & =se(c)\times sp(c),\\ c_{CZ} & =\underset{c\in ℜ}{arg max}CZ\left(c\right). \end{array}$$
4. **Index of union criteria** (IU): The index of union, as outlined by Unal (2017), represents one of the latest criteria. It operates on the premise that the optimal cutoff occurs where sensitivity and specificity are simultaneously close to the AUC, while also minimizing the difference between sensitivity and specificity.
(7) $$\begin{array}{cc} IU(c) & =|se(c)-AUC|+|sp(c)-AUC|,\\ c_{IU} & =\underset{c\in ℜ}{arg min}IU\left(c\right) \end{array}$$

As previously mentioned, sensitivity and specificity are functions of $F_{0}$ and $F_{1}$ (from Equation 3). By substituting Equation (3) into Equations (4)–(7), we can express the optimal cutoffs in terms of $F_{0}$ and $F_{1}$ as follows:

(8) $$\begin{array}{cc} c_{J} & =\underset{c\in ℜ}{arg max}J\left(c\right)=\underset{c\in ℜ}{arg max}\left\{F_{0}\left(c\right)-F_{1}\left(c\right)\right\}\\ c_{ER} & =\underset{c\in ℜ}{arg min}ER\left(c\right)=\underset{c\in ℜ}{arg min}\left\{\sqrt{{\left(F_{1}\left(c\right)\right)}^{2}+{\left(1-F_{0}\left(c\right)\right)}^{2}}\right\}\\ c_{CZ} & =\underset{c\in ℜ}{arg max}CZ\left(c\right)=\underset{c\in ℜ}{arg max}\left\{\left(1-F_{1}\left(c\right)\right)\times F_{0}\left(c\right)\right\}\\ c_{IU} & =\underset{c\in ℜ}{arg min}IU\left(c\right)\\ & =\underset{c\in ℜ}{arg min}\{|1-F_{1}\left(c\right)-AUC|+|F_{0}\left(c\right)-AUC\left|\right\}. \end{array}$$

### 2.2 Alternative ROC Framework: PV‐Based Model

Unlike the conventional approach of individually modeling $F_{0}$ and $F_{1}$ within the general ROC curve framework, this alternative framework utilizes PV. PV can be conceptualized as a standardization of the diseased biomarkers ($Y_{1}$) with respect to the distribution of healthy biomarkers ($F_{0}$). It quantifies the separation between healthy and diseased biomarkers (Pepe 2003). Given $Y_{0}$ and $Y_{1}$, PV can be calculated as

(9) $$Z=1-F_{0}\left(Y_{1}\right).$$

The adoption of PV proves beneficial, as it can be shown that the distribution function of Z corresponds to the ROC curve. This alternative framework leveraging PV enables the direct modeling of the ROC curve. Furthermore, this approach streamlines the incorporation of covariates into the model as needed.

#### 2.2.1 Special PV‐Based ROC Frameworks

Assuming F represents the CDF of the PV random variable Z, representing the ROC curve, the PV‐based framework requires the modeling of $F_{0}$ and F. Hence, the overall structure of modeling PV‐based ROC is

(10) $$\begin{array}{cc} Y_{0} & ∼F_{0}\left(\cdot \right),\\ Z & =1-F_{0}\left(Y_{1}\right),\\ \eta^{-1}\left(Z\right) & ∼F(\cdot ), \end{array}$$

where η is a suitable transformation on the PV. Based on how we specify F, η can be chosen accordingly. Based on how $F_{0}$ and F are specified, different types of PV‐based models can be proposed. For example, Chen and Ghosal (2021) showed the performances of the PV‐based models by choosing Gaussian distributions for both $F_{0}$ and F, and using both logit and probit links as η. Later, Ghosal and Chen (2022) proposed a transformed normal PV‐regression model by accounting for covariates and using similar distributional assumptions. Stanley and Tubbs (2018) used a quantile regression approach for estimating the covariate‐adjusted conditional distribution of $F_{0}$ and assumed a Beta distribution for F and used an identity link for η. There are also examples of semiparametric and nonparametric considerations for $F_{0}$ and F in the literature (de Carvalho and Rodriguez‐Alvarez 2018; Ghosal et al. 2022; de Carvalho and Rodríguez‐Álvarez 2022).

In this article, we employ several PV‐based frameworks, including a parametric PV (PV) model and a semiparametric PV (Semi.PV) model, to assess their efficacy in estimating the cutoffs. These models are delineated in Section A.2 in the Appendix.

#### 2.2.2 Optimal Cutoffs

In this section, we introduce the process of estimating cutoffs within the PV‐based framework, which depends on estimating $F_{0}$ and F. Now, based on Equation (9), we can write:

$$\begin{array}{cc} F_{0}\left(Y_{1}\right) & =1-Z,\\ \Rightarrow Y_{1} & =F_{0}^{-1}\left(1-Z\right). \end{array}$$

Then, analogous to Equation (3), the sensitivity and specificity for the PV‐based setup can be rewritten in terms of $F_{0}$ and F as

(11) $$\begin{array}{cc} sp(c) & =P\left[Y_{0}<c\right]=F_{0}\left(c\right),\\ se(c) & =P\left[Y_{1}>c\right]=P\left[F_{0}^{-1}\left(1-Z\right)>c\right]\\ & =1-P[F_{0}^{-1}\left(1-Z\right)<c]\\ & =1-P[1-z<F_{0}\left(c\right)]\\ & =1-P[z>1-F_{0}\left(c\right)]\\ & =P[z<1-F_{0}\left(c\right)]\\ & =F\left(1-F_{0}\left(c\right)\right). \end{array}$$

Hence, substituting the above into Equations (4)–(7), we can express the optimal cutoffs for a PV‐based framework in terms of $F_{0}$ and F as

(12) $$\begin{array}{cc} c_{J} & =\underset{c\in ℜ}{arg max}J\left(c\right)=\underset{c\in ℜ}{arg max}\left\{F_{0}\left(c\right)+F\left(1-F_{0}\left(c\right)\right)-1\right\}\\ c_{ER} & =\underset{c\in ℜ}{arg min}ER\left(c\right)=\underset{c\in ℜ}{arg min}\left\{\sqrt{{\left(1-F\left(1-F_{0}\left(c\right)\right)\right)}^{2}+{\left(1-F_{0}\left(c\right)\right)}^{2}}\right\}\\ c_{CZ} & =\underset{c\in ℜ}{arg max}CZ\left(c\right)=\underset{c\in ℜ}{arg max}\left\{F\left(1-F_{0}\left(c\right)\right)\times F_{0}\left(c\right)\right\}\\ c_{IU} & =\underset{c\in ℜ}{arg min}IU\left(c\right)=\underset{c\in ℜ}{arg min}\{|F\left(1-F_{0}\left(c\right)\right)-AUC|+|F_{0}\left(c\right)-AUC\left|\right\}. \end{array}$$

### 2.3 Covariate Adjustment

In this section, we extend the optimal cutoff estimating frameworks to allow for covariates in the estimation of the cutoffs. For the sake of simplicity, we illustrate the adjustment for one covariate, however, it can easily be extended for multiple covariates. Assume that $X_{0}$ and $X_{1}$ are, respectively, the covariates for the healthy and diseased biomarker groups. In the next subsections, we will introduce the covariates in the general and the PV‐based framework, and lay out the forms of optimal cutoffs for the corresponding frameworks.

#### 2.3.1 Covariates in General ROC Framework

For the general framework of the ROC curve, we can model $Y_{0}$ and $Y_{1}$ on the corresponding covariates as

$$\begin{array}{cc} Y_{0} & ∼F_{0}\left(\cdot |X_{0}\right)\;\text{and}\;Y_{1}∼F_{1}\left(\cdot |X_{1}\right). \end{array}$$

A covariate x‐specific ROC and AUC estimates will have the forms given as

(13) $$\begin{array}{cc} ROC_{x}\left(t\right) & =1-F_{1}\left(F_{0}^{-1}\left(1-t|X_{0}=x\right)|X_{1}=x\right),\;t\in \left(0,1\right),\\ AUC_{x} & =\int_{0}^{1}ROC_{x}\left(t\right)dt. \end{array}$$

Furthermore, the covariate x‐specific sensitivity and specificity can be written as

$$\begin{array}{cc} se(c|x) & =P\left[Y_{1}>c|X_{1}=x\right]=1-F_{1}\left(c|x\right),\\ sp(c|x) & =P\left[Y_{0}<c|X_{0}=x\right]=F_{0}\left(c|x\right). \end{array}$$

Following Equation (8), we can introduce covariates in the cutoff estimators as

(14) $$\begin{array}{cc} c_{J,x} & =\underset{c\in ℜ}{arg max}J_{x}\left(c\right)=\underset{c\in ℜ}{arg max}\left\{F_{0}\left(c|x\right)-F_{1}\left(c|x\right)\right\}\\ c_{ER,x} & =\underset{c\in ℜ}{arg min}ER_{x}\left(c\right)=\underset{c\in ℜ}{arg min}\left\{\sqrt{{\left(F_{1}\left(c|x\right)\right)}^{2}+{\left(1-F_{0}\left(c|x\right)\right)}^{2}}\right\}\\ c_{CZ,x} & =\underset{c\in ℜ}{arg max}CZ_{x}\left(c\right)=\underset{c\in ℜ}{arg max}\left\{\left(1-F_{1}\left(c|x\right)\right)\times F_{0}\left(c|x\right)\right\}\\ c_{IU,x} & =\underset{c\in ℜ}{arg min}IU_{x}\left(c\right)\\ & =\underset{c\in ℜ}{arg min}\{|1-F_{1}\left(c|x\right)-AUC_{x}\left|+\right|F_{0}\left(c|x\right)-AUC_{x}\left|\right\}, \end{array}$$

where $AUC_{x}$ is the covariate x‐specific estimate of AUC.

Given this structure, covariates can be incorporated into various frameworks of ROC curve modeling. For instance, when considering covariates within the widely popular BN framework, we anticipate employing separate linear regression models to characterize the healthy and diseased biomarkers, as illustrated below

$$\begin{array}{cc} y_{0i} & =\beta_{00}+\beta_{10}X_{0i}+\varepsilon_{0i},\;\varepsilon_{0i}∼N\left(0,\sigma_{0}^{2}\right),\;i=1,2,\text{…},n_{i},\\ y_{1j} & =\beta_{01}+\beta_{11}X_{1j}+\varepsilon_{1j},\;\varepsilon_{1j}∼N\left(0,\sigma_{1}^{2}\right),\;j=1,2,\text{…},n_{j}, \end{array}$$

where i and j, respectively, corresponds to healthy and diseased subjects, $n_{k}$ corresponds to the sample size, $\beta_{0k}$ and $\beta_{1k}$’s are different intercept and slope parameters, and $\varepsilon_{k}$’s are the errors corresponding to kth groups with k=0,1. Then, following the conventional BN framework in Section A.1, we have the covariate x‐specific BN similar to (A3) as

$$\begin{array}{c} a_{x}=\frac{\left(\beta_{01}-\beta_{00}\right)+\left(\beta_{11}-\beta_{10}\right)\cdot x}{\sigma_{1}},\;b=\frac{\sigma_{0}}{\sigma_{1}}, \end{array}$$

and the covariate x‐specific ROC and AUC estimates:

$$\begin{array}{cc} ROC_{x}\left(t\right) & =\Phi (a_{x}+b\cdot \Phi^{-1}\left(t\right)),\;t\in \left(0,1\right),\\ AUC_{x} & =\Phi \left(\frac{a_{x}}{\sqrt{1+b^{2}}}\right). \end{array}$$

We can specify $F_{0}\left(\cdot |X_{0}=x\right)=\Phi \left(\cdot ;\beta_{00}+\beta_{10}x,\sigma_{0}\right)$ and $F_{1}\left(\cdot |X_{1}=x\right)=\Phi \left(\cdot ;\beta_{01}+\beta_{11}x,\sigma_{1}\right)$ in (14) to estimate BN version of different cutoffs, where Φ(y) corresponds to a standard normal CDF obtained at y and Φ(y;μ,σ) denotes a normal CDF with mean μ and standard deviation (SD) σ obtained at y.

#### 2.3.2 Covariates in PV‐Based Framework

In the PV‐based framework, PV is estimated by incorporating the diseased biomarker $Y_{1}$ and corresponding covariate $X_{1}$ in the healthy biomarker CDF $F_{0}$ as:

$$\begin{array}{cc} z|(X_{1}=x_{1}) & =1-F_{0}\left(y_{1}|X_{0}=x_{1}\right). \end{array}$$

Then, the covariate x‐specific ROC curve and AUC have the form in terms of Z as

(15) $$\begin{array}{cc} ROC_{x}\left(t\right) & =F\left(t|X_{1}=x\right)=P\left[Z<t|X_{1}=x\right],\;t\in \left(0,1\right),\\ AUC_{x} & =\int_{0}^{1}ROC_{x}\left(t\right)dt. \end{array}$$

Then, the covariate x‐specific sensitivity and specificity (following (11)) can be written as:

(16) $$\begin{array}{cc} sp(c|x) & =P\left[Y_{0}<c|X_{0}=x\right]=F_{0}\left(c|x\right),\\ se(c|x) & =F\left(1-F_{0}\left(c|X_{0}=x\right)|X_{1}=x\right). \end{array}$$

Then, based on Equation (12), we have the following forms of covariate‐specific optimal cutoffs for the PV‐based setup:

(17) $$\begin{array}{cc} c_{J,x} & =\underset{c\in ℜ}{arg max}J_{x}\left(c\right)=\underset{c\in ℜ}{arg max}\left\{F_{0}\left(c|x\right)+F\left(1-F_{0}\left(c|x\right)|x\right)-1\right\}\\ c_{ER,x} & =\underset{c\in ℜ}{arg min}ER_{x}\left(c\right)\\ & =\underset{c\in ℜ}{arg min}\left\{\sqrt{{\left(1-F\left(1-F_{0}\left(c|x\right)|x\right)\right)}^{2}+{\left(1-F_{0}\left(c|x\right)\right)}^{2}}\right\}\\ c_{CZ,x} & =\underset{c\in ℜ}{arg max}CZ_{x}\left(c\right)=\underset{c\in ℜ}{arg max}\left\{F\left(1-F_{0}\left(c|x\right)|x\right)\times F_{0}\left(c|x\right)\right\}\\ c_{IU,x} & =\underset{c\in ℜ}{arg min}IU_{x}\left(c\right)\\ & =\underset{c\in ℜ}{arg min}\{|F\left(1-F_{0}\left(c|x\right)|x\right)-AUC_{x}\left|+\right|F_{0}\left(c|x\right)-AUC_{x}\left|\right\}. \end{array}$$

For a parametric PV regression model (similar to one in Section A.2), we can accommodate covariates and estimate covariate x‐specific ROC and AUC in the following way:

$$\begin{array}{cc} y_{0i} & =\beta_{00}+\beta_{10}X_{0i}+\varepsilon_{0i},\;\varepsilon_{0i}∼N\left(0,\sigma_{0}^{2}\right),\\ z|(X_{1}=x_{1}) & =1-\Phi \left(y_{1};\beta_{00}+\beta_{10}x_{1},\sigma_{0}\right),\\ ROC_{x}\left(t\right) & =\Phi \left(\Phi^{-1}\left(t\right);\beta_{0}+\beta_{1}x,\sigma \right),\;t\in \left(0,1\right),\\ AUC_{x} & =\int_{0}^{1}ROC_{x}\left(t\right)dt. \end{array}$$

Finally, we can specify $F_{0}\left(\cdot |X_{0}=x\right)=\Phi \left(\cdot ;\beta_{00}+\beta_{10}x,\sigma_{0}\right)$ and $F\left(\cdot |X_{1}=x\right)=\Phi \left(\cdot ;\beta_{0}+\beta_{1}x,\sigma \right)$ in (17) to estimate parametric PV version of different cutoffs.

The inclusion of covariates in the Semi.PV model proceeds in a similar manner. For further details, please refer to Equation (A6) and consult Ghosal et al. (2022) and de Carvalho et al. (2017).

### 2.4 Bayesian Computational Aspects

We take a Bayesian approach for the inference purposes discussed so far. We use proper objective priors for all the parameters. Specifically, each of the mean parameters μ’s along with regression coefficients such as intercept ($\beta_{0}$’s) and slope ($\beta_{1}$’s) parameters follow N(0,100) priors and variance parameter $\sigma^{2}$ follows IG(0.01,0.01).

We use RJAGS to implement Monte Carlo Markov chain (MCMC) algorithms to generate samples from the posterior distribution of the model parameters given the data. Both visual inspection of the trace plots and diagnostic tools (Gelman and Rubin 1992) are used to ensure convergence of the MCMC chains. After convergence, we thin the iterations to produce a sample of 5000 to produce posterior means, SD, and 95% credible intervals (CI). The algorithm is implemented in R (R Core Team 2023).

## 3 Simulation

To assess the efficacy of various optimal cutoff estimators across diverse ROC estimation methodologies, we have conducted extensive simulations. These simulations encompass scenarios both with and without the inclusion of covariates, which will be detailed in the following two subsections.

### 3.1 Simulation Without Covariate

A list of simulation scenarios is considered in this subsection to understand the performances of different cutoff methodologies for different ROC curve estimation frameworks. The primary objective of this simulation exercise is to assess the performance of various cutoff metrics across a range of conditions, including different data‐generating mechanisms, sample sizes, and AUC values. The simulation settings vary concerning different data‐generating mechanisms, varying AUC levels (low, medium, and high), and sample sizes (small, medium, and high). The different data‐generating mechanisms mimic that of Hajian‐Tilaki et al. (1997) and Faraggi and Reiser (2002) to ensure that the biomarker values are generated from varying distributions (normal, skewed, and mixture distributions). The true parameters were chosen to produce AUC values that fall into low, medium, and high categories. The details of the scenarios are tabulated in Table 1. The true values of the AUCs and optimal cutoffs corresponding to different scenarios are tabulated in Section A of the online Supporting Information.

**TABLE 1:** Details of simulation mechanism without covariate.

Data‐generating mechanisms	Fitting models	AUC level	Sample size
BN equal	Empirical (Emp)	Low	Small (N=50)
BN unequal	Binormal (BN)	Medium	Medium (N=100)
Skewed I	Kernel‐based nonparametric (NonPar)	High	High (N=500)
Skewed II	Placement value‐based parametric (PV)		
Skewed III	Placement value‐based semiparametric (Semi.PV)		
Mixed I			
Mixed II			

By varying the types of data‐generating mechanisms and AUC levels, for each sample size we have 7×3=21 distinct simulation settings. For each of the simulation settings, a series of ROC curve estimation methodologies have been used including several parametric, nonparametric, and semiparametric methods mentioned in Table 1 to estimate AUC, ROC, and the four different optimal cutoffs discussed in Section 2.1.2. We create 1000 data replicates and report the median and IQR (interquartile range) of biases obtained by different ROC estimation methods to estimate AUC and four different optimal cutoffs. We also plot the biases incurred by different ROC methods for different simulation mechanisms. We opted for median and IQR instead of mean and SD due to occasional skewness in the distribution of cutoff estimates obtained from different data replicates. In addition, this choice aligns with the presentation in the figures for consistency. The details of the data‐generating mechanism and corresponding true parameter values are tabulated in Table 2. Subsequently, the true AUC and cutoff estimates are tabulated in Table A.1 of the Supporting Information. In this paper, we will illustrate the simulation results with the medium sample size, that is, N=100 for both healthy and diseased biomarker samples. The simulation results for the rest two sample sizes will be described in the Supporting Information.

**TABLE 2:** Data generation mechanism details with true parameters, no covariate framework.

Data‐generating	Data	True parameter		
scenario	generation	Low AUC	Medium AUC	High AUC
BN equal	$Y_{0}∼N\left(\mu_{0},\;\sigma^{2}\right)$	$\mu_{0}=0$, $\mu_{1}=0.2$,	$\mu_{0}=0$, $\mu_{1}=1$,	$\mu_{0}=0$, $\mu_{1}=2.5$,
	$Y_{1}∼N\left(\mu_{1},\;\sigma^{2}\right)$	σ=1	σ=1	σ=1
BN unequal	$Y_{0}∼N\left(\mu_{0},\;\sigma_{0}^{2}\right)$	$\mu_{0}=0$, $\mu_{1}=0.2$,	$\mu_{0}=0$, $\mu_{1}=1$,	$\mu_{0}=1$, $\mu_{1}=2.9$,
	$Y_{1}∼N\left(\mu_{1},\;\sigma_{1}^{2}\right)$	$\sigma_{0}=1.2$, $\sigma_{1}=0.8$	$\sigma_{0}=1.2$, $\sigma_{1}=0.5$	$\sigma_{0}=0.5$, $\sigma_{1}=1.2$
Skewed I	$Y_{0}^{\frac{1}{2}}∼N\left(\mu_{0},\;\sigma_{0}^{2}\right)$	$\mu_{0}=0$, $\mu_{1}=0.2$,	$\mu_{0}=0$, $\mu_{1}=1$,	$\mu_{0}=1$, $\mu_{1}=2.5$,
	$Y_{1}^{\frac{1}{2}}∼N\left(\mu_{1},\;\sigma_{1}^{2}\right)$	$\sigma_{0}=1.2$, $\sigma_{1}=1$	$\sigma_{0}=1$, $\sigma_{1}=0.7$	$\sigma_{0}=1$, $\sigma_{1}=0.5$
Skewed II	$log\left(Y_{0}\right)∼N\left(\mu_{0},\;\sigma_{0}^{2}\right)$	$\mu_{0}=0$, $\mu_{1}=0.2$,	$\mu_{0}=0$, $\mu_{1}=1$,	$\mu_{0}=1$, $\mu_{1}=2.5$,
	$log\left(Y_{1}\right)∼N\left(\mu_{1},\;\sigma_{1}^{2}\right)$	$\sigma_{0}=1$, $\sigma_{1}=1$	$\sigma_{0}=1$, $\sigma_{1}=0.7$	$\sigma_{0}=1$, $\sigma_{1}=0.5$
Skewed III	$Y_{0}∼Gamma\left(k,\;\theta_{0}\right)$	k=0.5, $\theta_{0}=0.1$	k=0.5, $\theta_{0}=0.1$,	k=0.5, $\theta_{0}=0.1$,
	$Y_{0}∼Gamma\left(k,\;\theta_{1}\right)$	$\theta_{1}=0.15$	$\theta_{1}=0.6$	$\theta_{1}=7$
	k: shape, $\theta_{j}$: scale			
Mixed I	$Y_{0}∼N\left(\mu_{0},\;\sigma_{0}^{2}\right)$	$\mu_{0}=0$, $\sigma_{0}=1$,	$\mu_{0}=0$, $\sigma_{0}=1$,	$\mu_{0}=0$, $\sigma_{0}=1$,
	$Y_{1}∼N(\pi \mu_{11}+\left(1-\pi \right)\mu_{12}$,	π=0.5,	π=0.5,	π=0.5,
	$\pi^{2}\sigma_{11}^{2}+{\left(1-\pi \right)}^{2}\sigma_{12}^{2})$	$\mu_{11}=0$, $\sigma_{11}=1$	$\mu_{11}=0$, $\sigma_{11}=1$	$\mu_{11}=0$, $\sigma_{11}=1$
		$\mu_{12}=1$, $\sigma_{12}=5$	$\mu_{12}=4$, $\sigma_{12}=5$	$\mu_{12}=8$, $\sigma_{12}=5$
Mixed II	$Y_{0}∼N(\pi_{0}\mu_{01}+\left(1-\pi_{0}\right)\mu_{02}$,	$\mu_{01}=0$, $\sigma_{01}=1$,	$\mu_{01}=0$, $\sigma_{01}=1$,	$\mu_{01}=0$, $\sigma_{01}=1$,
	$\pi_{0}^{2}\sigma_{01}^{2}+{\left(1-\pi_{0}\right)}^{2}\sigma_{02}^{2})$	$\mu_{02}=1$, $\sigma_{02}=2$,	$\mu_{02}=1$, $\sigma_{02}=5$,	$\mu_{02}=1$, $\sigma_{02}=5$,
	$Y_{1}∼N(\pi_{1}\mu_{11}+\left(1-\pi_{1}\right)\mu_{12}$,	$\pi_{0}=0.5$	$\pi_{0}=0.5$	$\pi_{0}=0.5$
	$\pi_{1}^{2}\sigma_{11}^{2}+{\left(1-\pi_{1}\right)}^{2}\sigma_{12}^{2})$	$\mu_{11}=0$, $\sigma_{11}=1$,	$\mu_{11}=0$, $\sigma_{11}=1$,	$\mu_{11}=0$, $\sigma_{11}=1$,
		$\mu_{12}=1.5$, $\sigma_{12}=2.5$,	$\mu_{12}=2.5$, $\sigma_{12}=2.5$,	$\mu_{12}=5$, $\sigma_{12}=2.5$,
		$\pi_{1}=0.4$	$\pi_{1}=0.4$	$\pi_{1}=0.4$

Table 3a, 3b, 3c presents the median and IQR of biases in estimating AUC and four optimal cutoff estimates across various ROC fitting models under different data‐generating mechanisms, specifically focusing on a medium sample size of N=100. When the data are generated from “BN equal,” all models exhibit similar performance across different AUC levels. However, at low AUC level, the estimation of Youden's index shows higher variability. Conversely, for data generated from “BN unequal,” the empirical (Emp) and kernel‐based (NonPar) models perform less effectively compared to the binormal (BN) and PV‐based parametric (BN) and semiparametric (Semi.PV) models. For the “Skewed I” data‐generating mechanism, Emp and NonPar demonstrate minimal biases in estimating the cutoffs at low and medium AUC levels, with BN and PV models also performing well. In the case of “Skewed II” data‐generating mechanism, Emp and NonPar consistently perform satisfactorily across all AUC levels, while Semi.PV occasionally outperforms them, especially at low AUC levels. Here, the performance of the Semi.PV model occasionally emerges as the superior choice, notably excelling in certain scenarios such as estimating J and IU at low AUC level, and estimating J at medium AUC level. Furthermore, in the majority of cases, it closely rivals the performance of the Emp and NonPar models. Conversely, while the performance of the BN and PV models is not subpar, they seldom achieve top rankings, with only occasional instances of outperforming others, such as in estimating ER at high AUC levels. In the context of the “Skewed III” mechanism, Emp and NonPar maintain consistent performance at low AUC levels, while Semi.PV and BN models display the lowest bias at medium and high AUC levels, respectively. For the “Mixed I” and “Mixed II” mechanisms, BN and PV models exhibit the least bias, with Semi.PV closely following. In the case of these final two mechanisms, the performances of the Emp and NonPar models were notably suboptimal, attributed to the data‐generating mechanism involving a mixture of normals. Interestingly, these mechanisms mirrored the “BN unequal” mechanism, yielding comparable findings.

**TABLE 3a:** Biases of estimating AUC and optimal cutoffs for different fitting models for medium sample size, low AUC, no covariate framework.

Data‐generating mechanism	Fitting model	Median ± IQR				
		AUC	J	ER	CZ	IU
BN equal	Emp	0.001 ± 0.053	0.001 ± 0.099	0.002 ± 0.097	0.001 ± 0.096	0.001 ± 0.092
	BN	0.000 ± 0.050	0.008 ± 0.646	−0.001 ± 0.113	−0.002 ± 0.124	−0.001 ± 0.107
	NonPar	0.000 ± 0.052	0.001 ± 0.130	0.001 ± 0.111	0.001 ± 0.110	0.001 ± 0.098
	PV	0.001 ± 0.050	0.003 ± 0.645	0.000 ± 0.113	−0.001 ± 0.124	−0.001 ± 0.107
	Semi.PV	0.000 ± 0.049	0.009 ± 0.651	0.001 ± 0.114	0.001 ± 0.123	0.000 ± 0.108
BN unequal	Emp	0.001 ± 0.052	0.726 ± 0.109	0.163 ± 0.105	0.200 ± 0.105	0.013 ± 0.095
	BN	0.001 ± 0.051	−0.001 ± 0.190	−0.001 ± 0.103	0.000 ± 0.114	0.001 ± 0.101
	NonPar	0.001 ± 0.052	0.715 ± 0.176	0.156 ± 0.122	0.194 ± 0.128	0.014 ± 0.103
	PV	0.001 ± 0.051	−0.001 ± 0.190	−0.001 ± 0.103	0.000 ± 0.114	0.001 ± 0.101
	Semi.PV	−0.001 ± 0.049	0.001 ± 0.195	0.001 ± 0.106	0.001 ± 0.114	−0.006 ± 0.100
Skewed I	Emp	−0.049 ± 0.058	1.014 ± 0.152	1.007 ± 0.150	1.008 ± 0.151	0.995 ± 0.172
	BN	−0.048 ± 0.056	1.603 ± 6.038	1.021 ± 0.302	1.026 ± 0.313	1.021 ± 0.145
	NonPar	−0.048 ± 0.057	1.016 ± 0.187	1.000 ± 0.177	1.003 ± 0.169	0.956 ± 0.259
	PV	−0.048 ± 0.055	1.595 ± 5.970	1.020 ± 0.301	1.025 ± 0.310	1.021 ± 0.145
	Semi.PV	0.085 ± 0.149	1.367 ± 3.105	1.253 ± 1.204	1.330 ± 1.087	1.005 ± 1.062
Skewed II	Emp	0.001 ± 0.053	0.714 ± 0.259	0.697 ± 0.250	0.697 ± 0.255	0.672 ± 0.292
	BN	−0.011 ± 0.052	1.769 ± 2.853	0.843 ± 0.516	0.883 ± 0.624	0.719 ± 0.226
	NonPar	0.000 ± 0.052	0.713 ± 0.299	0.675 ± 0.292	0.676 ± 0.288	0.596 ± 0.488
	PV	−0.012 ± 0.053	1.684 ± 2.689	0.818 ± 0.407	0.851 ± 0.496	0.718 ± 0.224
	Semi.PV	0.068 ± 0.151	0.459 ± 4.221	0.733 ± 1.537	0.932 ± 1.491	0.565 ± 1.330
Skewed III	Emp	−0.002 ± 0.059	0.001 ± 0.009	0.031 ± 0.010	0.030 ± 0.009	0.031 ± 0.010
	BN	0.012 ± 0.051	0.056 ± 0.023	0.043 ± 0.013	0.047 ± 0.016	0.033 ± 0.010
	NonPar	−0.003 ± 0.058	0.001 ± 0.011	0.030 ± 0.011	0.030 ± 0.011	0.029 ± 0.017
	PV	0.012 ± 0.052	0.053 ± 0.022	0.042 ± 0.012	0.045 ± 0.015	0.033 ± 0.010
	Semi.PV	0.061 ± 0.136	0.039 ± 0.131	0.048 ± 0.056	0.050 ± 0.059	0.033 ± 0.047
Mixed I	Emp	0.002 ± 0.056	−1.142 ± 0.205	−0.389 ± 0.197	−0.577 ± 0.205	0.065 ± 0.180
	BN	0.000 ± 0.051	0.004 ± 0.172	−0.001 ± 0.126	0.001 ± 0.144	0.001 ± 0.144
	NonPar	0.002 ± 0.055	−1.089 ± 0.685	−0.358 ± 0.258	−0.543 ± 0.287	0.033 ± 0.188
	PV	0.000 ± 0.051	0.003 ± 0.171	−0.003 ± 0.127	0.000 ± 0.144	−0.002 ± 0.145
	Semi.PV	−0.002 ± 0.052	−0.029 ± 0.179	−0.030 ± 0.13	−0.043 ± 0.141	−0.004 ± 0.148
Mixed II	Emp	−0.016 ± 0.053	−0.798 ± 0.136	−0.159 ± 0.129	−0.208 ± 0.133	−0.083 ± 0.121
	BN	−0.017 ± 0.050	−0.006 ± 0.280	0.003 ± 0.130	−0.001 ± 0.143	−0.048 ± 0.132
	NonPar	−0.017 ± 0.052	−0.777 ± 0.187	−0.143 ± 0.148	−0.192 ± 0.152	−0.088 ± 0.137
	PV	−0.018 ± 0.050	−0.005 ± 0.280	0.003 ± 0.130	−0.001 ± 0.143	−0.048 ± 0.131
	Semi.PV	−0.018 ± 0.052	−0.008 ± 0.278	−0.001 ± 0.132	−0.004 ± 0.140	−0.049 ± 0.133

These findings are visually depicted in Figures B.1–B.6 of the Supporting Information for medium sample size, while Tables B.3a–B.4c in the Supporting Information present biases corresponding to low and high sample sizes. The trends observed in the medium sample size roughly align with those from low and high sample sizes, as demonstrated in Figures B.7–B.18.

### 3.2 Simulation With Covariate

In simulation with covariates, we consider similar scenarios as without covariates. The simulation settings vary for different data‐generating mechanisms and sample sizes. For each of the simulation settings, only the BN, PV, and Semi.PV models are used to estimate AUC and four different cutoffs at different covariate levels because of the inability of the rest of the models (empirical and nonparametric models) to accommodate covariates. Similar to the simulation without covariates, we selected the true parameters in the covariate case so that the resulting AUC values for different covariate levels would fall into various categories within the plausible AUC range. The details of the scenarios are tabulated in Table 4. The true values of the AUCs and optimal cutoffs corresponding to different scenarios of simulation with covariates are also tabulated in Section A of the online Supporting Information.

**TABLE 3b:** Biases of estimating AUC and optimal cutoffs for different fitting models for medium sample size, medium AUC, no covariate framework.

Data‐generating mechanism	Fitting model	Median ± IQR				
		AUC	J	ER	CZ	IU
BN equal	Emp	0.003 ± 0.042	0.003 ± 0.096	0.002 ± 0.095	0.003 ± 0.095	0.002 ± 0.095
	BN	0.000 ± 0.041	0.000 ± 0.142	0.000 ± 0.089	0.002 ± 0.095	−0.001 ± 0.139
	NonPar	0.001 ± 0.042	0.004 ± 0.111	0.002 ± 0.104	0.002 ± 0.106	0.003 ± 0.104
	PV	−0.001 ± 0.041	0.000 ± 0.141	0.000 ± 0.089	0.002 ± 0.095	0.000 ± 0.139
	Semi.PV	−0.004 ± 0.043	−0.001 ± 0.142	−0.004 ± 0.092	0 ± 0.098	−0.001 ± 0.137
BN unequal	Emp	0.003 ± 0.045	0.170 ± 0.092	−0.049 ± 0.085	0.061 ± 0.088	−0.106 ± 0.098
	BN	−0.001 ± 0.043	−0.001 ± 0.074	−0.001 ± 0.062	−0.001 ± 0.064	0.004 ± 0.089
	NonPar	0.000 ± 0.045	0.165 ± 0.106	−0.045 ± 0.09	0.058 ± 0.096	−0.080 ± 0.133
	PV	−0.001 ± 0.043	−0.001 ± 0.075	−0.001 ± 0.061	−0.001 ± 0.064	0.004 ± 0.089
	Semi.PV	−0.003 ± 0.043	−0.003 ± 0.075	−0.003 ± 0.065	−0.001 ± 0.067	0.001 ± 0.09
Skewed I	Emp	−0.169 ± 0.054	1.118 ± 0.157	0.982 ± 0.155	1.038 ± 0.154	1.048 ± 0.173
	BN	−0.200 ± 0.056	1.498 ± 0.718	1.036 ± 0.191	1.116 ± 0.219	1.095 ± 0.160
	NonPar	−0.170 ± 0.053	1.102 ± 0.193	0.960 ± 0.202	1.016 ± 0.206	0.996 ± 0.329
	PV	−0.200 ± 0.056	1.497 ± 0.720	1.035 ± 0.190	1.115 ± 0.218	1.095 ± 0.160
	Semi.PV	−0.166 ± 0.133	1.688 ± 2.817	1.166 ± 1.125	1.230 ± 1.099	1.151 ± 0.998
Skewed II	Emp	0.005 ± 0.041	1.098 ± 0.263	0.859 ± 0.268	0.950 ± 0.267	0.981 ± 0.290
	BN	−0.086 ± 0.058	1.590 ± 0.629	0.921 ± 0.291	1.140 ± 0.339	1.061 ± 0.313
	NonPar	0.002 ± 0.041	1.052 ± 0.370	0.808 ± 0.402	0.904 ± 0.385	0.911 ± 0.479
	PV	−0.083 ± 0.059	1.548 ± 0.552	0.901 ± 0.277	1.107 ± 0.307	1.076 ± 0.300
	Semi.PV	−0.054 ± 0.212	0.922 ± 2.416	0.925 ± 1.585	1.010 ± 1.665	0.972 ± 1.447
Skewed III	Emp	−0.001 ± 0.047	0.064 ± 0.032	0.105 ± 0.034	0.093 ± 0.034	0.102 ± 0.037
	BN	−0.032 ± 0.032	0.075 ± 0.031	0.069 ± 0.022	0.089 ± 0.026	0.024 ± 0.017
	NonPar	−0.002 ± 0.047	0.060 ± 0.037	0.099 ± 0.043	0.088 ± 0.041	0.095 ± 0.054
	PV	0.015 ± 0.038	0.050 ± 0.030	0.055 ± 0.021	0.064 ± 0.025	0.034 ± 0.018
	Semi.PV	0.034 ± 0.158	0.024 ± 0.097	0.043 ± 0.066	0.038 ± 0.074	0.038 ± 0.063
Mixed I	Emp	0.003 ± 0.046	−0.376 ± 0.193	0.094 ± 0.184	−0.141 ± 0.183	0.249 ± 0.224
	BN	−0.001 ± 0.044	0.003 ± 0.147	0.001 ± 0.135	0.002 ± 0.136	0.007 ± 0.174
	NonPar	0.002 ± 0.046	−0.364 ± 0.237	0.085 ± 0.199	−0.140 ± 0.215	0.180 ± 0.324
	PV	−0.001 ± 0.045	−0.005 ± 0.147	0.000 ± 0.136	−0.003 ± 0.140	0.005 ± 0.173
	Semi.PV	−0.005 ± 0.053	−0.096 ± 0.174	−0.059 ± 0.158	−0.099 ± 0.173	−0.003 ± 0.203
Mixed II	Emp	−0.036 ± 0.047	−0.400 ± 0.136	−0.059 ± 0.130	−0.134 ± 0.134	−0.206 ± 0.127
	BN	−0.038 ± 0.045	−0.002 ± 0.192	−0.001 ± 0.121	0.000 ± 0.129	−0.124 ± 0.139
	NonPar	−0.037 ± 0.046	−0.389 ± 0.172	−0.057 ± 0.151	−0.129 ± 0.154	−0.214 ± 0.140
	PV	−0.038 ± 0.045	−0.002 ± 0.192	0 ± 0.122	0.000 ± 0.129	−0.125 ± 0.138
	Semi.PV	−0.041 ± 0.046	−0.007 ± 0.190	−0.004 ± 0.125	−0.005 ± 0.130	−0.128 ± 0.142

**TABLE 3c:** Biases of estimating AUC and optimal cutoffs for different fitting models for medium sample size, high AUC, no covariate framework.

Data‐generating mechanism	Fitting model	Median ± IQR				
		AUC	J	ER	CZ	IU
BN equal	Emp	0.003 ± 0.015	0.001 ± 0.095	0.002 ± 0.095	0.002 ± 0.095	0.001 ± 0.095
	BN	−0.001 ± 0.014	0.003 ± 0.100	0.004 ± 0.115	0.004 ± 0.103	0.003 ± 0.100
	NonPar	0.001 ± 0.016	0.004 ± 0.107	0.002 ± 0.103	0.002 ± 0.107	0.004 ± 0.106
	PV	−0.003 ± 0.014	0.003 ± 0.100	0.003 ± 0.114	0.004 ± 0.103	0.003 ± 0.100
	Semi.PV	−0.006 ± 0.016	0.001 ± 0.104	−0.001 ± 0.117	0.001 ± 0.107	0.001 ± 0.104
BN unequal	Emp	0.001 ± 0.026	0.147 ± 0.086	0.282 ± 0.088	0.179 ± 0.087	0.217 ± 0.089
	BN	0.000 ± 0.024	0.003 ± 0.072	0.004 ± 0.079	0.004 ± 0.076	0.002 ± 0.114
	NonPar	0.001 ± 0.026	0.137 ± 0.097	0.269 ± 0.111	0.168 ± 0.101	0.199 ± 0.125
	PV	0.001 ± 0.024	−0.001 ± 0.077	0.007 ± 0.080	0.001 ± 0.077	0.005 ± 0.108
	Semi.PV	−0.014 ± 0.035	−0.075 ± 0.178	−0.056 ± 0.142	−0.079 ± 0.172	−0.067 ± 0.140
Skewed I	Emp	0.004 ± 0.029	−5.615 ± 0.257	−4.709 ± 0.255	−4.834 ± 0.256	−3.383 ± 0.253
	BN	−0.010 ± 0.039	−5.626 ± 0.240	−4.731 ± 0.324	−4.840 ± 0.259	−3.392 ± 0.238
	NonPar	0.000 ± 0.028	−5.647 ± 0.325	−4.740 ± 0.303	−4.866 ± 0.307	−3.414 ± 0.308
	PV	−0.011 ± 0.039	−5.626 ± 0.240	−4.731 ± 0.324	−4.840 ± 0.257	−3.393 ± 0.238
	Semi.PV	−0.012 ± 0.092	−5.089 ± 1.587	−4.124 ± 1.296	−4.294 ± 1.439	−2.843 ± 1.370
Skewed II	Emp	0.005 ± 0.029	3.044 ± 0.735	2.268 ± 0.740	2.813 ± 0.736	2.809 ± 0.735
	BN	−0.060 ± 0.056	3.334 ± 0.932	2.007 ± 0.885	2.855 ± 0.785	2.972 ± 0.917
	NonPar	0.001 ± 0.028	2.889 ± 1.258	2.116 ± 1.080	2.662 ± 1.112	2.656 ± 1.037
	PV	−0.058 ± 0.056	3.300 ± 0.914	2.003 ± 0.878	2.828 ± 0.770	3.000 ± 0.857
	Semi.PV	−0.022 ± 0.120	3.209 ± 4.981	2.979 ± 4.202	3.256 ± 4.472	3.486 ± 4.105
Skewed III	Emp	0.000 ± 0.027	1.512 ± 0.381	1.578 ± 0.381	1.528 ± 0.381	1.570 ± 0.381
	BN	−0.164 ± 0.032	0.093 ± 0.048	0.076 ± 0.036	0.101 ± 0.047	−0.044 ± 0.020
	NonPar	0.000 ± 0.027	1.438 ± 0.514	1.505 ± 0.514	1.455 ± 0.514	1.497 ± 0.514
	PV	0.037 ± 0.021	1.542 ± 0.349	1.609 ± 0.349	1.559 ± 0.349	1.600 ± 0.349
	Semi.PV	−0.005 ± 0.079	0.154 ± 1.265	0.161 ± 1.075	0.141 ± 1.179	0.155 ± 0.743
Mixed I	Emp	0.001 ± 0.027	0.349 ± 0.184	0.637 ± 0.185	0.413 ± 0.184	0.529 ± 0.194
	BN	−0.001 ± 0.025	0.008 ± 0.152	0.009 ± 0.162	0.008 ± 0.156	0.006 ± 0.237
	NonPar	0.001 ± 0.027	0.328 ± 0.199	0.608 ± 0.231	0.392 ± 0.206	0.494 ± 0.266
	PV	0.002 ± 0.024	−0.007 ± 0.160	0.015 ± 0.165	−0.002 ± 0.160	0.024 ± 0.222
	Semi.PV	−0.016 ± 0.040	−0.186 ± 0.398	−0.129 ± 0.304	−0.187 ± 0.382	−0.149 ± 0.303
Mixed II	Emp	−0.036 ± 0.026	−0.016 ± 0.126	0.113 ± 0.128	0.034 ± 0.127	−0.018 ± 0.126
	BN	−0.041 ± 0.027	0.001 ± 0.122	0.006 ± 0.138	0.002 ± 0.122	0.000 ± 0.122
	NonPar	−0.037 ± 0.027	−0.019 ± 0.144	0.108 ± 0.138	0.030 ± 0.137	−0.022 ± 0.138
	PV	−0.042 ± 0.027	0.001 ± 0.123	0.006 ± 0.137	0.002 ± 0.122	0.000 ± 0.122
	Semi.PV	−0.045 ± 0.027	−0.018 ± 0.128	−0.003 ± 0.140	−0.014 ± 0.129	−0.019 ± 0.128

**TABLE 4:** Details of simulation mechanism with covariate.

Data‐generating scenario	Fitting models	Sample size
BN	Binormal (BN)	Small (N=50)
Skewed	Placement value‐based parametric (PV)	Medium (N=100)
Mixed	Placement value‐based semiparametric (Semi.PV)	High (N=500)

First, we generate the healthy and diseased covariates from uniform distributions. Each of $X_{0}$ and $X_{1}$ was generated from U(−0.5,1.5) where U(a,b) is a continuous uniform distribution with support a≤x≤b. Then, we generate 1000 data replicates based on the covariates from different mechanisms and report median and IQR of biases obtained by different ROC estimation methods to estimate AUC and four different optimal cutoffs obtained at x=0 and x=1. We also plot the biases incurred by different ROC methods for different simulation mechanisms. The details of the data‐generating mechanisms and corresponding true parameter values are tabulated in Table 5.

**TABLE 5:** Data generation mechanism details with true parameters, with covariate.

Data‐generating mechanism	Data generation	True parameters
BN	$Y_{0}∼N\left(b_{00}+b_{01}X_{0},\;\sigma_{0}^{2}\right)$	$b_{00}=1$, $b_{01}=1$, $\sigma_{0}=1$
	$Y_{1}∼N\left(b_{10}+b_{11}X_{1},\;\sigma_{1}^{2}\right)$	$b_{10}=1.5$, $b_{11}=2$, $\sigma_{1}=1$
Skewed	$Y_{0}∼Gamma\left(k,\;\theta_{0}\right)$	$b_{00}=3$, $b_{01}=0.1$
	$Y_{0}∼Gamma\left(k,\;\theta_{1}\right)$	$b_{10}=5$, $b_{11}=9$
	k: shape, $\theta_{j}$: scale	k=2
	$\theta_{0}=b_{00}+b_{01}X_{0}$	
	$\theta_{1}=b_{10}+b_{11}X_{1}$	
Mixed	$Y_{0}∼N\left(\mu_{0},\;\sigma_{0}^{2}\right)$	$a_{00}=0$, $a_{01}=1$, $\sigma_{0}=1$
	$Y_{1}∼N\left(\mu_{1},\;\sigma_{1}^{2}\right)$	$a_{101}=0$, $a_{111}=1$
	$\mu_{0}=a_{00}+a_{01}X_{0}$	$a_{102}=1$, $a_{112}=5$
	$\mu_{1}=\pi \mu_{11}+\left(1-\pi \right)\mu_{12}$	$\sigma_{1}=1.5$
	$\mu_{11}=a_{101}+a_{111}X_{0}$	π=0.5
	$\mu_{12}=a_{102}+a_{112}X_{1}$	

Table 6 illustrates the median and IQR of biases in estimating AUC and four different optimal cutoff estimates from various ROC fitting models at covariate values x=0 and x=1. When data are generated from the “BN” mechanism, it becomes evident that the BN and PV models exhibit the least biases across both covariate levels, owing to their correct model specification. Similar trends are observed for the “Mixed” data‐generating mechanism. In these cases, the Semi.PV model's performance closely rivals that of the BN and PV models, particularly when estimating ER at x=0. However, when the data‐generating mechanism deviates from the BN and PV models, as seen in the “Skewed” data‐generating mechanism, the Semi.PV model consistently demonstrates the least median biases, except when estimating IU. Interestingly, IU is estimated most effectively by the BN model at both covariate levels. In addition, it is noted that the estimation of AUC and optimal cutoff points from the Semi.PV model results in higher variability, as evidenced by the elevated IQR estimates across all data‐generating mechanisms.

**TABLE 6:** Biases of estimating AUC and optimal cutoffs for different fitting models at different covariate levels for medium sample size, with covariate framework.

Data‐generating mechanism	Fitting model	Median ± IQR				
		AUC	J	ER	CZ	IU
**x = 0**						
BN	BN	0.001 ± 0.068	−0.001 ± 0.283	0.001 ± 0.128	0.002 ± 0.134	0.001 ± 0.158
	PV	0.002 ± 0.068	−0.002 ± 0.277	0.000 ± 0.127	0.002 ± 0.133	−0.003 ± 0.276
	Semi.PV	−0.040 ± 0.139	−0.012 ± 0.587	0.002 ± 0.233	0.007 ± 0.251	−0.016 ± 0.402
Skewed	BN	−0.076 ± 0.044	4.958 ± 1.407	2.748 ± 0.915	3.812 ± 0.998	−0.527 ± 0.734
	PV	−0.035 ± 0.050	3.840 ± 1.288	2.159 ± 0.786	2.818 ± 0.835	1.823 ± 0.907
	Semi.PV	−0.033 ± 0.138	2.553 ± 3.672	1.582 ± 2.800	1.880 ± 2.843	1.738 ± 2.795
Mixed	BN	0.001 ± 0.070	−0.001 ± 0.271	0.002 ± 0.154	−0.002 ± 0.159	−0.436 ± 0.168
	PV	0.002 ± 0.070	−0.010 ± 0.271	0.000 ± 0.154	−0.003 ± 0.159	−0.020 ± 0.164
	Semi.PV	−0.027 ± 0.126	0.044 ± 0.588	0.002 ± 0.285	−0.014 ± 0.298	−0.042 ± 0.362
**x = 1**						
BN	BN	−0.004 ± 0.044	0.000 ± 0.132	0.002 ± 0.127	0.000 ± 0.125	0.000 ± 0.132
	PV	−0.002 ± 0.044	−0.001 ± 0.131	0.002 ± 0.128	0.001 ± 0.125	0.047 ± 0.230
	Semi.PV	−0.031 ± 0.165	−0.044 ± 0.319	−0.052 ± 0.355	−0.050 ± 0.353	−0.051 ± 0.539
Skewed	BN	0.015 ± 0.032	2.057 ± 1.289	1.694 ± 1.242	2.175 ± 1.277	−0.869 ± 1.613
	PV	0.035 ± 0.028	1.354 ± 1.315	1.513 ± 1.266	1.560 ± 1.295	−3.494 ± 1.065
	Semi.PV	−0.004 ± 0.104	0.326 ± 4.133	0.700 ± 4.007	0.476 ± 4.05	−3.004 ± 4.435
Mixed	BN	−0.002 ± 0.032	0.007 ± 0.154	0.003 ± 0.165	0.004 ± 0.156	−0.185 ± 0.159
	PV	−0.002 ± 0.031	0.004 ± 0.154	0.001 ± 0.165	0.002 ± 0.156	−0.645 ± 0.277
	Semi.PV	−0.033 ± 0.149	−0.052 ± 0.331	−0.065 ± 0.419	−0.076 ± 0.403	−0.720 ± 0.497

These findings are further supported by bias plots depicted in Figures B.19–B.20 of the Supporting Information for medium sample size. Similar simulations have been conducted for low (N=50) and high (N=500) sample sizes, and the overall conclusions drawn from the medium sample size simulations are consistent across different sample sizes. See the Supporting Information for more details. The corresponding bias tables for other sample sizes can be found in Tables B.5–B.6, and similar bias plots are available in Figures B.21– B.24.

## 4 Data Application

Data used in this article were obtained from the Alzheimer's Disease Neuroimaging Initiative (ADNI) database^1^ (adni.loni.usc.edu). The ADNI was launched in 2003 as a public–private partnership, led by Principal Investigator Michael W. Weiner, MD. The primary goal of ADNI has been to test whether serial magnetic resonance imaging, positron emission tomography, other biological markers, and clinical and neuropsychological assessment can be combined to measure the progression of mild cognitive impairment and early AD.

In this context, our aim is to assess the diagnostic accuracy and determine the optimal cutoffs for various fluid biomarkers in AD diagnosis. The focus biomarkers include plasma amyloid‐β (Aβ42) (Teunissen et al. 2018), tau (total‐tau or t‐tau) (Holper et al. 2022), and phosphorylated tau (p‐tau) (Gonzalez‐Ortiz et al. 2023). To achieve this, we utilized the data set from the ADSP Phenotype Harmonization Consortium (PHC), which collected fluid biomarker levels from various studies and cohorts, then merged the biomarker data across these cohorts. The fluid biomarker scores were harmonized across data sets such as ADNI, the National Alzheimer's Coordinating Center (NACC), and the Memory and Aging Project at Knight Alzheimer's Disease Research Center (MAP at Knight ADRC). Subsequently, the scores were co‐calibrated and standardized to create z‐score versions of the biomarkers. Table 7 illustrates the overall summary of the ADSP data.

**TABLE 7:** ADSP PHC standardized fluid biomarker data. p‐value (p) corresponds to the tests to compare covariates between the normal cognition group and the AD group. In the table, biomarkers include Aβ42, Tau, and pTau. Rest are listed as covariates.

Covariates & biomarkers	Overall	Normal cognition	AD	p
	N = 682	N = 360 (52.79%)	N = 322 (47.21%)	
Continuous	Mean (SD)			
Aβ42	−0.03 (1.01)	0.52 (0.88)	−0.65 (0.77)	<0.001
Tau	0.03 (1.06)	−0.50 (0.86)	0.63 (0.93)	<0.001
pTau	0.14 (1.03)	−0.23 (0.96)	0.56 (0.95)	<0.001
Age	74.95 (7.08)	74.53 (6.41)	75.41 (7.75)	0.107
Categorical covariates	N (%)			
Sex				0.001
Male	362 (53.1)	169 (46.9)	193 (59.9)	
Female	320 (46.9)	191 (53.1)	129 (40.1)	
Race				0.010
American Indian/Alaskan Native	1 (0.1)	1 (0.3)	0 (0.0)	
Asian	9 (1.3)	3 (0.8)	6 (1.9)	
African American	27 (4.0)	21 (5.8)	6 (1.9)	
White	637 (93.4)	328 (91.1)	309 (96.0)	
>1 Race	8 (1.2)	7 (1.9)	1 (0.3)	

Among the 682 patients in the ADSP data set, 52.79% exhibit normal cognition, while the remainder are diagnosed with AD. Table 7 illustrates variations in standardized biomarker levels (p<0.001 for all biomarkers) between the disease groups, with the diseased cohort showing notably lower standardized Aβ42 values and higher standardized Tau and pTau values. While age does not differ much between disease groups (p=0.107), there are notable differences in sex and race distributions (p = 0.001 and 0.010, respectively).

In the subsequent two subsections, we will assess the overall diagnostic accuracy and optimal cutoffs for all three standardized biomarkers, as well as explore sex‐specific diagnostic accuracy and optimal cutoffs.

### 4.1 Overall Diagnostic Accuracy

**FIGURE 1:**
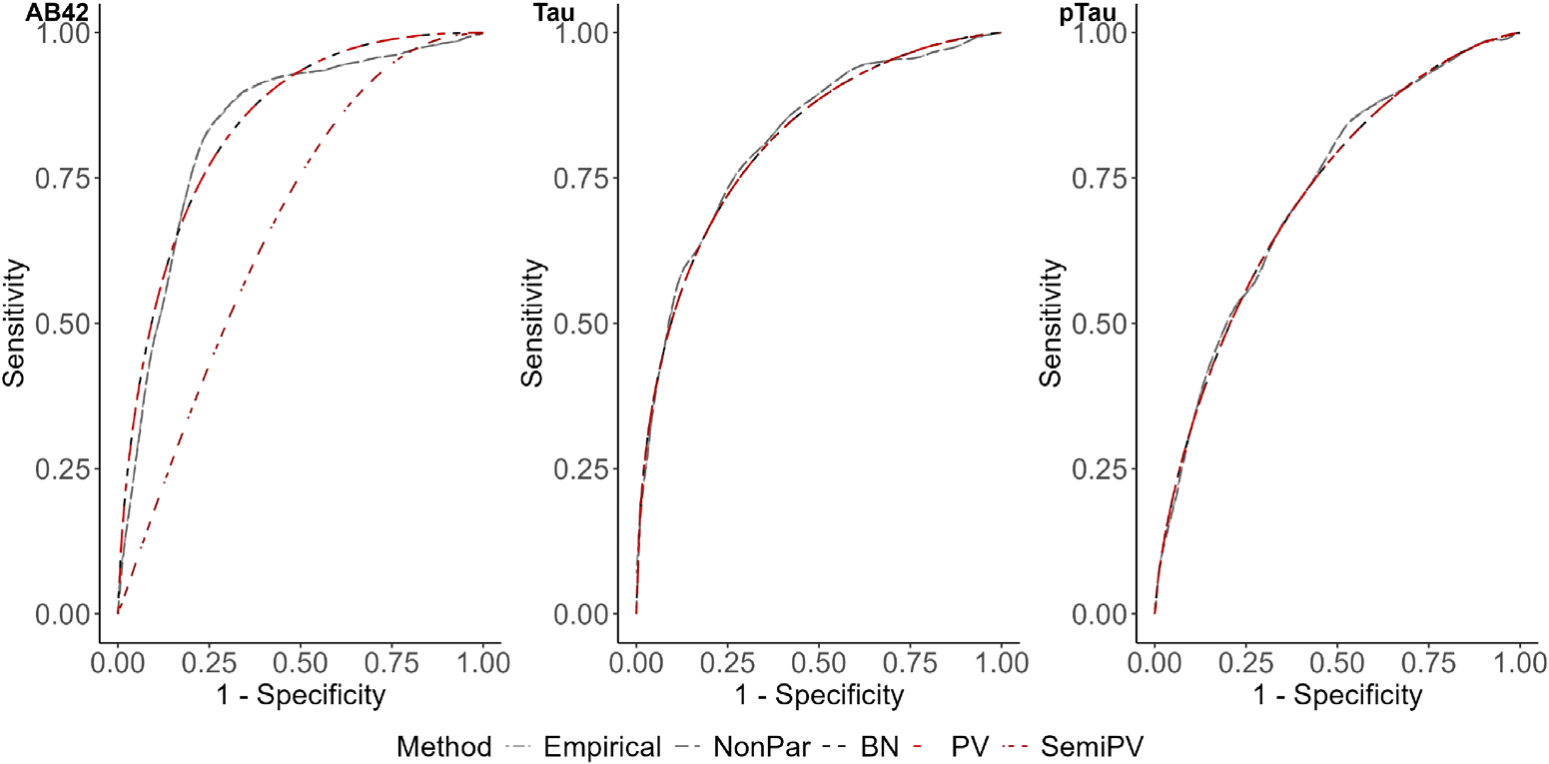
ROC curves for different ROC estimating methods for different biomarkers.

In this section, our focus lies in assessing the comprehensive diagnostic accuracy of the standardized fluid biomarkers Aβ42, Tau, and pTau. We aim to estimate the mean and 95% confidence interval (CI)t of AUC and four distinct optimal cutoffs obtained from the methodologies delineated in Section 3.1, encompassing Emp, NonPar, BN, PV, and Semi.PV. These results are consolidated in Table 8. To derive CI for the Emp and NonPar models, we utilize 1000 bootstrap samples. In addition, corresponding ROC curves are presented in Figure 1.

**TABLE 8:** Estimates of AUC and optimal cutoffs for different ROC estimating methods for different biomarkers.

Biomarker	Method	Metrics (Mean (95% CI))				
		AUC	J	ER	CZ	IU
Aβ42	Emp	0.833 (0.801, 0.865)	−0.037 (−0.164, 0.048)	−0.042 (−0.206, 0.037)	−0.041 (−0.200, 0.041)	−0.05 (−0.305, 0.030)
	NonPar	0.834 (0.800, 0.865)	−0.038 (−0.171, 0.048)	−0.042 (−0.211, 0.036)	−0.039 (−0.177, 0.038)	−0.049 (−0.284, 0.028)
	BN	0.841 (0.824, 0.857)	−0.026 (−0.066, 0.015)	−0.074 (−0.110, −0.036)	−0.053 (−0.089, −0.016)	−0.026 (−0.066, 0.015)
	PV	0.841 (0.829, 0.851)	−0.026 (−0.091, 0.038)	−0.074 (−0.139, −0.011)	−0.053 (−0.118, 0.010)	−0.026 (−0.091, 0.038)
	Semi.PV	0.667 (0.639, 0.694)	0.311 (0.006, 0.525)	0.093 (−0.136, 0.320)	0.127 (−0.116, 0.355)	0.098 (−0.135, 0.325)
Tau	Emp	0.815 (0.782, 0.847)	0.034 (−0.038, 0.101)	0.033 (−0.033, 0.098)	0.034 (−0.035, 0.100)	0.033 (−0.038, 0.098)
	NonPar	0.815 (0.783, 0.847)	0.032 (−0.050, 0.124)	0.032 (−0.036, 0.104)	0.032 (−0.040, 0.114)	0.032 (−0.041, 0.114)
	BN	0.812 (0.793, 0.830)	0.104 (0.056, 0.153)	0.064 (0.024, 0.103)	0.079 (0.039, 0.119)	0.104 (0.056, 0.153)
	PV	0.812 (0.798, 0.825)	0.104 (0.038, 0.171)	0.064 (0.001, 0.127)	0.079 (0.015, 0.143)	0.104 (0.038, 0.171)
	Semi.PV	0.812 (0.797, 0.825)	0.105 (0.038, 0.172)	0.064 (0.000, 0.127)	0.079 (0.014, 0.143)	0.105 (0.038, 0.172)
pTau	Emp	0.722 (0.685, 0.760)	0.143 (−0.114, 0.385)	0.144 (0.066, 0.218)	0.144 (0.062, 0.218)	0.145 (0.063, 0.221)
	NonPar	0.721 (0.682, 0.759)	0.138 (−0.169, 0.306)	0.143 (0.059, 0.223)	0.142 (0.052, 0.227)	0.142 (0.060, 0.223)
	BN	0.719 (0.697, 0.741)	0.156 (0.083, 0.232)	0.164 (0.123, 0.205)	0.162 (0.117, 0.206)	0.156 (0.083, 0.232)
	PV	0.719 (0.703, 0.734)	0.158 (0.078, 0.236)	0.164 (0.099, 0.232)	0.163 (0.095, 0.230)	0.158 (0.078, 0.236)
	Semi.PV	0.719 (0.702, 0.736)	0.160 (0.077, 0.241)	0.166 (0.095, 0.234)	0.164 (0.093, 0.235)	0.160 (0.077, 0.241)

**FIGURE 2:**
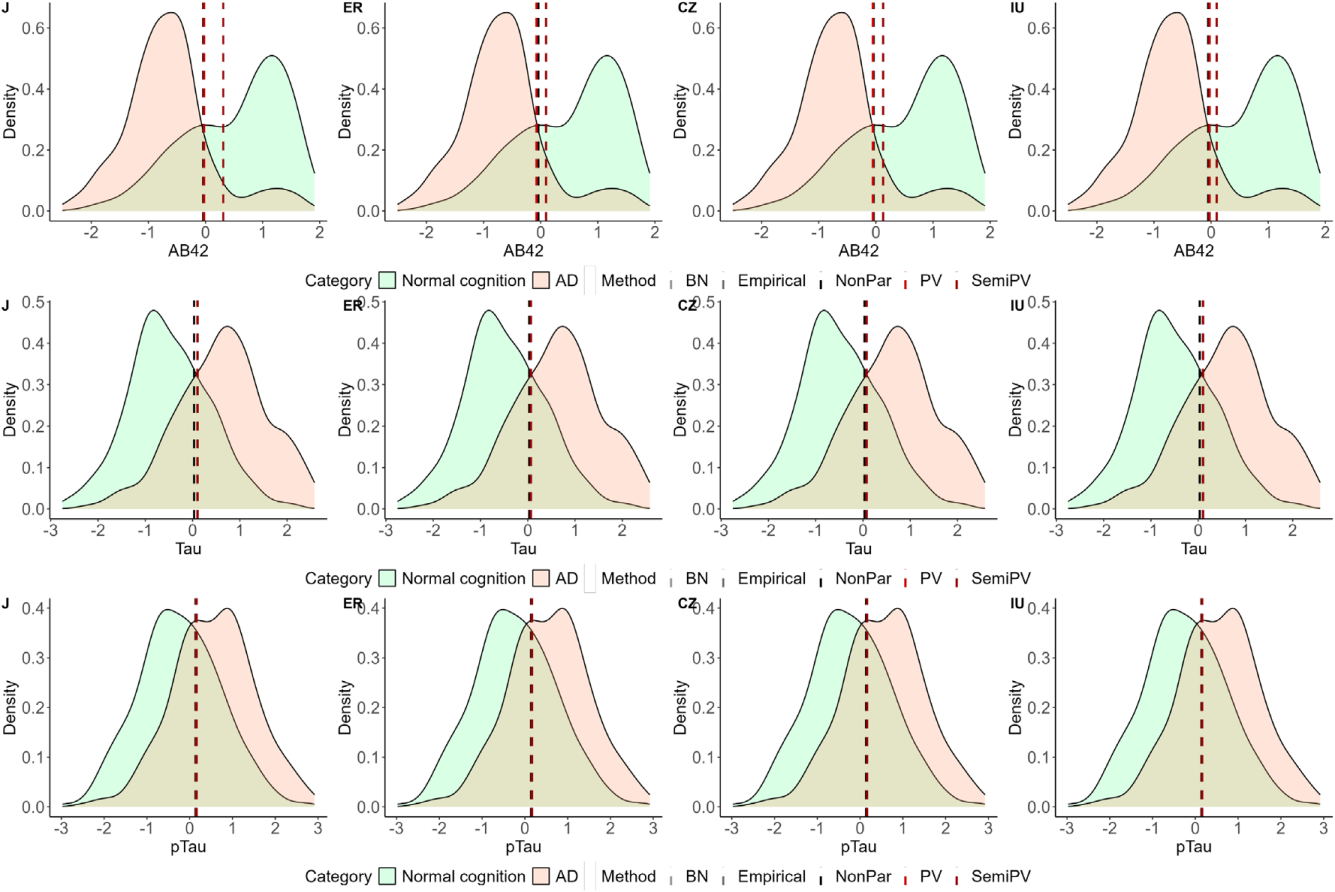
Biomarker densities and estimated cutoffs of all biomarkers. The three rows, respectively, correspond to the estimated cutoffs of all the optimal cutoff estimators for the biomarkers Aβ42, Tau, and pTau.

From Table 8, it is evident that the diagnostic accuracy of Aβ42 is notably high, ranging from 0.833 to 0.841, as estimated by most models, with the exception of Semi.PV, which yields a barely moderate AUC estimate. The optimal cutoff metrics for Aβ42 are consistently estimated similarly by the Emp and NonPar models. The BN and PV models also produce comparable yet distinct estimates compared to Emp and NonPar. However, the estimates from Semi.PV are notably different. It is important to note that for the biomarker Aβ42, values lower than the estimated cutoff would be classified as diseased. When comparing all the cutoffs produced by different ROC models, it is observed that the Emp and NonPar models exhibit low variability in their estimates, ranging from −0.059 to −0.037. Conversely, for BN and PV models, the variability in estimating different cutoffs is higher, spanning between −0.073 and −0.026. Notably, the Semi.PV model produces markedly different positive cutoff estimates, ranging from 0.093 to 0.311. For a better visual understanding, please refer to Figure 2.

When analyzing the biomarker Tau, we observe two distinct groups of estimates. The Emp and NonPar models comprise one group, while the remaining models form another. Despite the consistency in AUC estimates between groups (ranging from 0.812 to 0.815), the cutoff estimates differ notably. Similar to Aβ42, for Tau as well, the cutoffs exhibit minimal variability for Emp and NonPar across the four optimal cutoff metrics (ranging from 0.032 to 0.034). Conversely, for BN, PV, and Semi.PV, the cutoff estimates are higher. While ER and CZ have estimates of 0.064 and 0.079, respectively, the estimates for J and IU are even higher, ranging from around 0.104 to 0.105. For Tau, biomarker values higher than the cutoff would be classified as diseased.

The trends observed in the Tau results closely mirror those of pTau. However, pTau exhibits lower diagnostic accuracy, ranging from 0.719 to 0.722, compared to Aβ42 or Tau. The cutoff estimates obtained by Emp and NonPar range between 0.138 and 0.145, while those from the other models vary between 0.156 and 0.166. In this instance, unlike Tau, the estimates for ER and CZ from BN, PV, and Semi.PV (ranging from 0.162 to 0.166) are higher than the estimates for J and IU (ranging from 0.156 to 0.160). For pTau too, biomarker values higher than the cutoff would be classified as diseased.

### 4.2 Sex‐Specific Diagnostic Accuracy

**FIGURE 3:**
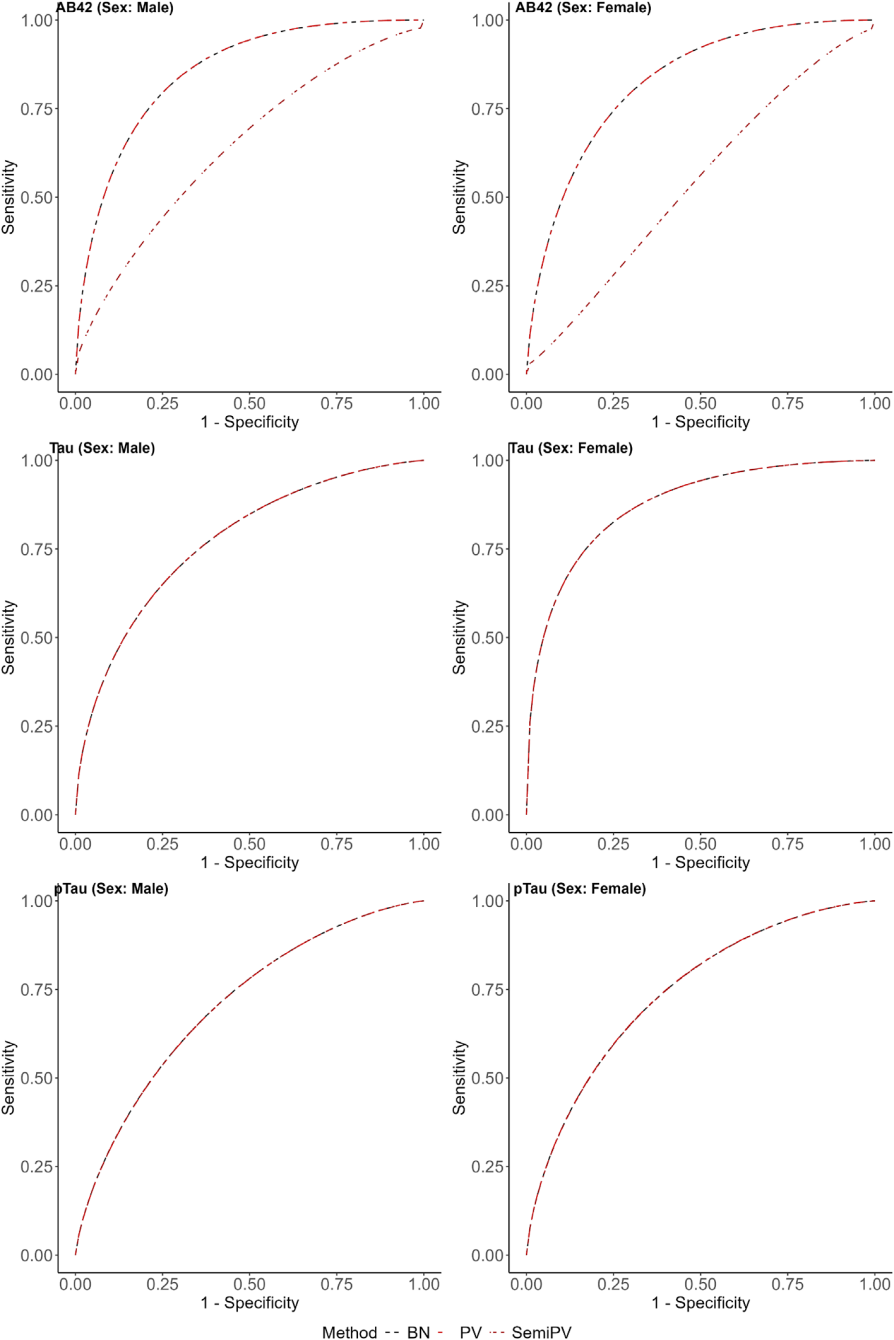
Sex‐specific ROC curves for different ROC estimating methods for different biomarkers.

In this section, we aim to examine whether potential covariates impact the diagnostic performances of biomarkers. Specifically, we aim to determine if biological sex influences diagnostic accuracy, assessing whether there are differences in diagnostic accuracy and optimal cutoffs between male and female patients. Similar to the previous section, our objective here is to estimate the mean and 95% CI of the AUC and four different cutoff metrics obtained from the methodologies employed in Section 3.2, namely, the BN, PV, and Semi.PV models, chosen for their ability to accommodate covariates. These results will be consolidated in Table 9, and the corresponding ROC curves will be presented in Figure 3.

**TABLE 9:** Sex‐specific estimates of AUC and optimal cutoffs for different ROC estimating methods for different biomarkers.

Biomarker	Covariate level	Method	Metrics (Mean (95% CI))				
		AUC	J	ER	CZ	IU
Aβ42	Sex: Male	BN	0.853 (0.833, 0.873)	−0.014 (−0.067, 0.037)	−0.058 (−0.109, −0.01)	−0.038 (−0.089, 0.011)	−0.014 (−0.067, 0.037)
		PV	0.853 (0.840, 0.866)	−0.014 (−0.102, 0.076)	−0.059 (−0.146, 0.029)	−0.038 (−0.125, 0.050)	−0.014 (−0.102, 0.076)
		Semi.PV	0.642 (0.505, 0.695)	−0.215 (−1.488, 0.617)	−0.168 (−1.470, 0.624)	−0.172 (−1.446, 0.622)	−0.186 (−1.761, 0.618)
	Sex: Female	BN	0.826 (0.801, 0.850)	−0.032 (−0.089, 0.028)	−0.083 (−0.135, −0.029)	−0.062 (−0.115, −0.008)	−0.032 (−0.089, 0.028)
		PV	0.826 (0.808, 0.843)	−0.031 (−0.121, 0.055)	−0.083 (−0.171, 0.003)	−0.062 (−0.150, 0.023)	−0.031 (−0.121, 0.055)
		Semi.PV	0.541 (0.436, 0.631)	−0.124 (−10.847, 10.322)	0.321 (−0.984, 2.104)	0.378 (−0.975, 2.452)	0.204 (−1.550, 1.873)
Tau	Sex: Male	BN	0.772 (0.744, 0.798)	−0.018 (−0.084, 0.048)	−0.044 (−0.096, 0.008)	−0.037 (−0.090, 0.017)	−0.018 (−0.084, 0.048)
		PV	0.772 (0.753, 0.790)	−0.020 (−0.109, 0.071)	−0.046 (−0.130, 0.040)	−0.038 (−0.122, 0.048)	−0.02 (−0.109, 0.071)
		Semi.PV	0.771 (0.752, 0.790)	−0.018 (−0.107, 0.072)	−0.044 (−0.129, 0.046)	−0.036 (−0.122, 0.054)	−0.018 (−0.107, 0.072)
	Sex: Female	BN	0.874 (0.852, 0.894)	0.239 (0.181, 0.298)	0.224 (0.166, 0.282)	0.232 (0.176, 0.289)	0.239 (0.181, 0.298)
		PV	0.873 (0.856, 0.889)	0.240 (0.156, 0.330)	0.225 (0.138, 0.314)	0.233 (0.147, 0.322)	0.240 (0.156, 0.330)
		Semi.PV	0.873 (0.856, 0.888)	0.240 (0.151, 0.331)	0.224 (0.137, 0.318)	0.232 (0.145, 0.324)	0.240 (0.151, 0.331)
pTau	Sex: Male	BN	0.707 (0.677, 0.736)	0.096 (0.006, 0.180)	0.108 (0.050, 0.166)	0.106 (0.044, 0.165)	0.096 (0.006, 0.180)
		PV	0.707 (0.686, 0.727)	0.097 (−0.009, 0.200)	0.108 (0.014, 0.201)	0.106 (0.012, 0.199)	0.097 (−0.009, 0.200)
		Semi.PV	0.707 (0.687, 0.727)	0.095 (−0.013, 0.203)	0.107 (0.010, 0.204)	0.104 (0.008, 0.203)	0.095 (−0.013, 0.203)
	Sex: Female	BN	0.742 (0.711, 0.771)	0.229 (0.147, 0.311)	0.239 (0.177, 0.302)	0.236 (0.173, 0.302)	0.229 (0.147, 0.311)
		PV	0.742 (0.718, 0.765)	0.229 (0.124, 0.336)	0.239 (0.144, 0.338)	0.236 (0.141, 0.335)	0.229 (0.124, 0.336)
		Semi.PV	0.741 (0.717, 0.764)	0.228 (0.129, 0.334)	0.238 (0.147, 0.334)	0.236 (0.143, 0.333)	0.228 (0.129, 0.334)

The sex‐specific AUC and optimal cutoff estimates in Table 9 echo the trends observed in Table 8. For the biomarker Aβ42, the BN and PV models yield nearly identical estimates of AUC and optimal cutoffs, while the Semi.PV model produces markedly different results. According to the BN and PV models, the diagnostic performance of Aβ42 is marginally higher for males (AUC = 0.853) compared to females (AUC = 0.826). The slope parameter ($\beta_{1}$ = 0.15, 95% CI = (0.037, 0.264)) corresponding to the sex variable in the PV regression model in Section 2.3.2 confirms the significance of this difference. In contrast, the Semi.PV model yields lower AUC values (0.642 for males and 0.541 for females). Moreover, the estimates of the cutoff points vary between these models. While the BN and PV models yield identical estimates for J and IU (−0.014), the ER (approximately −0.058) and CZ (−0.038) estimates differ. In contrast, the Semi.PV model yields distinct estimates of cutoff points for different sex groups, as detailed in the rows corresponding to the biomarker Aβ42 of Table 9.

For the biomarker Tau, the performance of all models exhibits notable similarity. However, based on the AUC estimates corresponding to Tau, we observe notably higher diagnostic capacity for females (AUC: 0.873–0.874) compared to males (AUC: 0.771–0.772), as evidenced by the slope parameter ($\beta_{1}$ = −0.582, 95% CI = (−0.716, −0.449)) corresponding to the sex variable from the PV regression model. Consequently, the cutoffs for males and females also differ remarkably. For males, all ROC models yield similar estimates of J, IU (−0.020 to −0.018), ER (−0.046 to −0.044), and CZ (−0.038 to −0.036). Conversely, for females, the pattern remains the same but with different cutoff estimates of J, IU (0.239–0.240), ER (0.224–0.225), and CZ (0.232–0.233).

In the case of the biomarker pTau, once again, the ROC models behave similarly as compared to Tau. Here, we observe marginally higher performance of pTau for females (AUC: 0.741–0.742) than males (AUC: 0.707). However, similar to Aβ42, the statistical difference is marginal, as the 95% CI of the slope parameter ($\beta_{1}$ = −0.148, 95% CI = (−0.278, −0.024)) corresponding to the sex variable from the PV model barely includes 0. The cutoff estimates remain consistent across all ROC models, with J, IU (0.095–0.097) estimates being similar, as well as ER (0.107–0.108) and CZ (0.104–0.106) estimates. The pattern remains consistent for females, with similar estimates of J, IU (0.228–0.229), ER (0.238–0.239), and CZ (0.236).

**FIGURE 4:**
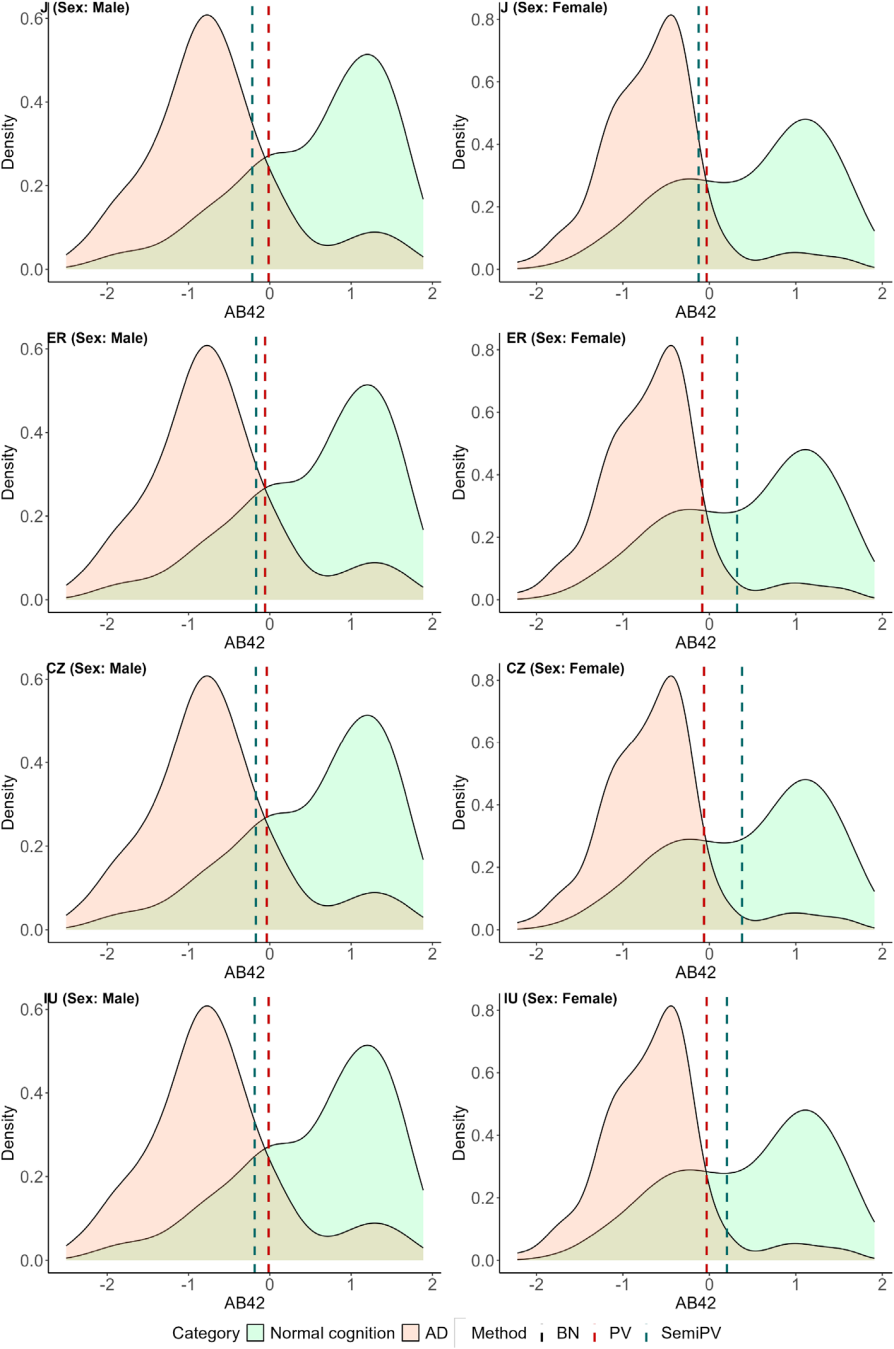
Sex‐specific biomarker densities and estimated cutoffs of biomarker Aβ42. The left row corresponds to the cutoff estimates of “Male” subjects and the right row corresponds to that of the “Female” subjects.

**FIGURE 5:**
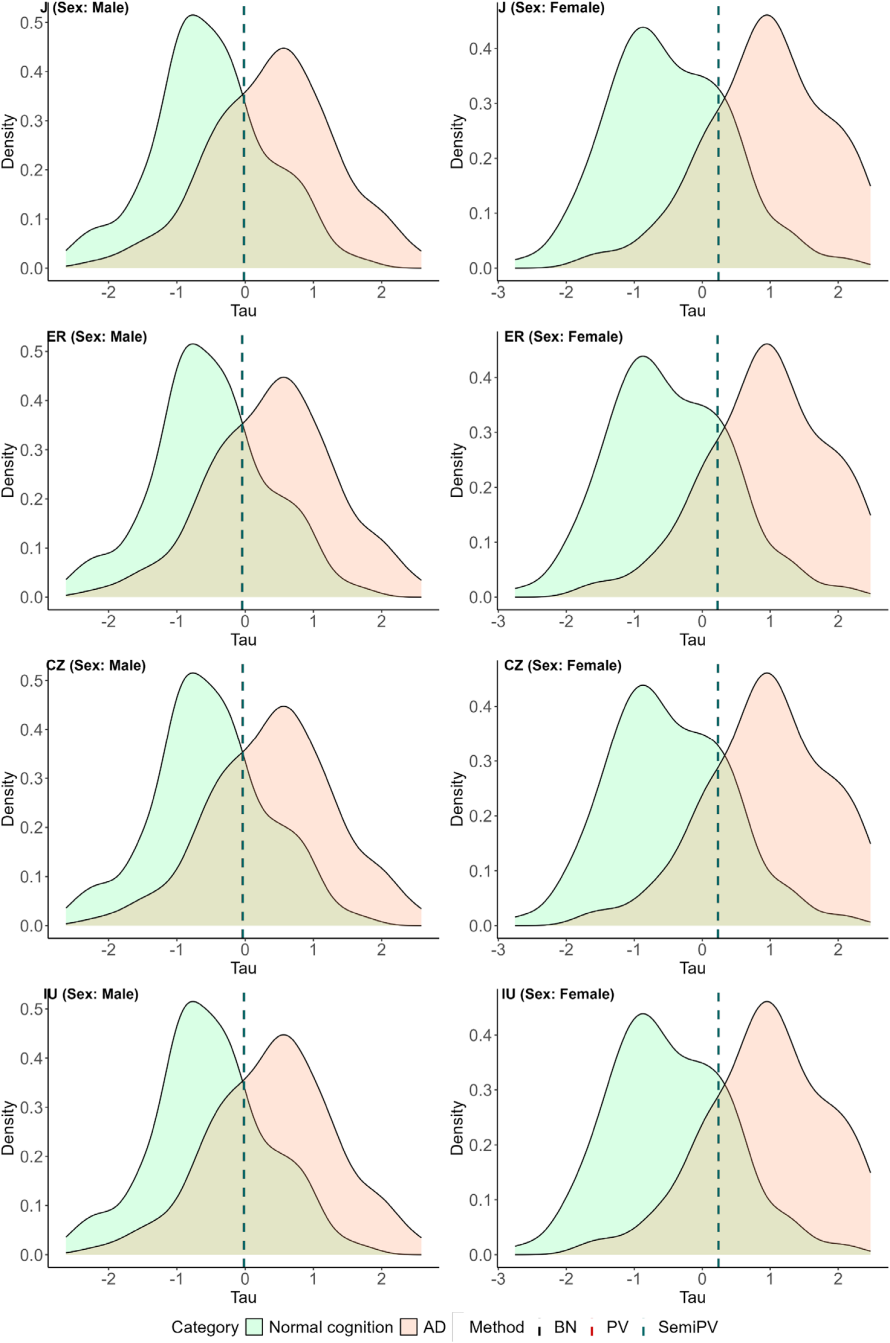
Sex‐specific biomarker densities and estimated cutoffs of biomarker Tau. The left row corresponds to the cutoff estimates of “Male” subjects and the right row corresponds to that of the “Female” subjects.

**FIGURE 6:**
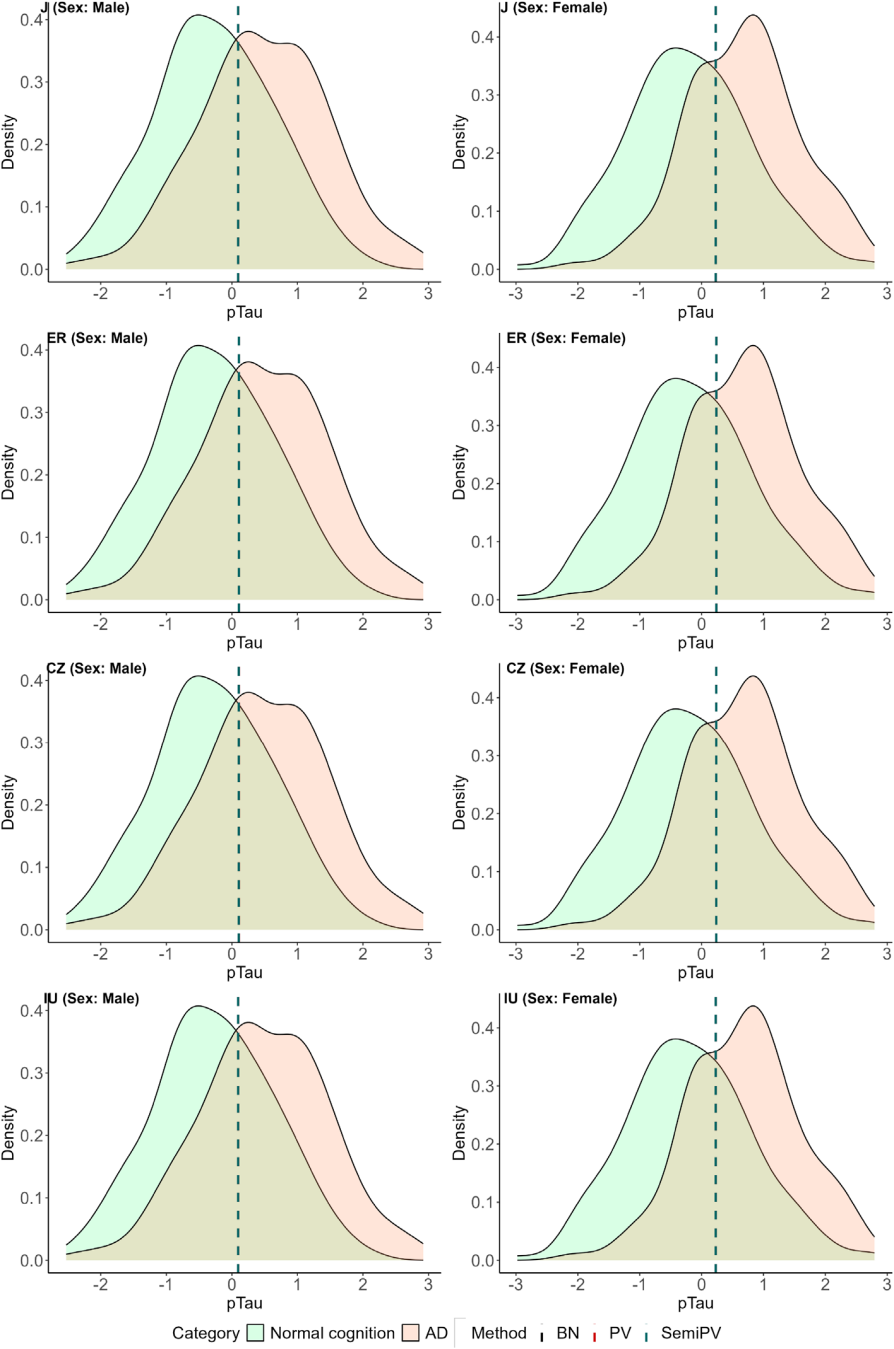
Sex‐specific biomarker densities and estimated cutoffs of biomarker pTau. The left row corresponds to the cutoff estimates of “Male” subjects and the right row corresponds to that of the “Female” subjects.

All of these cutoffs are visually represented on the healthy and diseased density plots of the three biomarkers in Figures 4, 5, 6.

## 5 Discussion

In this article, our aim was to evaluate the performance of various existing optimal cutoff techniques. While this task has been previously explored using a single ROC estimation technique, typically the empirical one, we hypothesized that such an approach might not provide a comprehensive understanding. Although the empirical ROC model is simple to fit, widely used, and often the default method for most of the available softwares; extensive simulations revealed few instances where its performance was inadequate, and alternative ROC modeling techniques proved to be more effective. In addition, the absence of well‐established options for estimating covariate‐specific optimal cutoffs prompted our investigation. This article not only presents some of these options but also compares their performances under various settings.

Through extensive simulation exercises, we found the empirical model (Emp) to be a powerful tool for estimating AUC and optimal cutoffs. Despite concerns regarding the smoothness of the empirical ROC curve, using a moderately dense set of points to estimate the empirical ROC curve might mitigate this issue. However, the Kernel‐based nonparametric (NonPar) model often outperformed the Emp model in estimating cutoffs. Moreover, the BN and PV models demonstrated robustness, particularly outperforming the Emp and NonPar models unless the biomarkers were generated from highly skewed models. The Semi.PV model, although the most complex, consistently performed well and competed with both parametric and nonparametric models, especially in situations with adequate sample sizes. Similar observations were made in simulations involving covariates: correctly specified parametric models exhibited superior performance, but as biomarkers became more skewed, the Semi.PV model demonstrated its superiority.

Regarding the choice of optimal cutoffs, in many scenarios, the estimation of J resulted in high bias and variability, particularly evident at low AUC levels with parametric and semiparametric models. Conversely, IU, CZ, and ER estimates remained more consistent across different scenarios. Although concave models were mentioned, they were consciously avoided in the simulation and data analysis due to their strict shape restrictions and tendency for bias in the event of model misspecification. We also deliberately excluded another framework for modeling ROC curves from our analysis, namely, the Lehmann family of ROC curves (Gönen and Heller 2010), due to the prerequisite of adhering to the Lehmann assumption.

The data analysis revealed distinct patterns in the behavior of various ROC estimating models and cutoff estimators. For the standardized Aβ42 biomarker, we identified three distinct groups of ROC estimating models based on their estimation characteristics. The first group, comprising the Emp and NonPar models, exhibited similar estimates of AUC and produced different optimal cutoffs with less variability. In contrast, the second group, consisting of the parametric models (BN and PV), yielded AUC estimates similar to the first group but different cutoff estimates, albeit with similar J and IU estimates. Notably, the third group exclusively comprised the Semi.PV model due to its markedly different AUC and cutoff estimates. The notably smaller AUC observed in this group stemmed from the clear bimodality in both healthy and diseased group densities, as depicted in the first row of Figure 2. Consequently, the estimated cutoffs from the Semi.PV model exhibited significant divergence. As for the biomarkers Tau and pTau, the majority of the aforementioned patterns persisted, albeit with the Semi.PV model exhibiting behavior more akin to the parametric models. This similarity may be attributed to the more bell‐shaped densities of the biomarkers. This pattern in the data analysis was also observed in the simulation scenarios as well.

The covariate‐specific analysis revealed important differences in biomarker performance between males and females. However, it is important to note a couple of caveats. First, our model only accounted for linear covariate effects, which may be limiting for continuous covariates like age. Future research could explore nonlinear covariate effects for more comprehensive insights. In addition, for the Aβ42 and pTau biomarkers, the slope parameters associated with sex in the PV regression models had confidence intervals that barely included 0, warranting cautious interpretation of findings for these biomarkers. Nevertheless, for Tau, there is strong evidence indicating divergent performance between males and females.

As noted in the literature (Leeflang et al. 2008), data‐driven selection of optimal cutoffs can often result in biased estimates of sensitivity and specificity, particularly in scenarios with limited sample sizes. This bias can lead to overly optimistic performance assessments. To mitigate this issue and ensure more reliable evaluations, further exploration in the future is required by combining advanced machine learning techniques and the discussed methodologies. These approaches can help to reduce the impact of bias and provide more accurate estimates of diagnostic test performance. Another limitation of our study is the assumption of a 50% prevalence for the diseased group. We followed the sample size choices from previous studies, most of which assumed equal sample sizes for both healthy and diseased groups, leading to a 50% prevalence. In future work, we plan to vary the prevalence to assess its impact on the performance of the cutoffs. Furthermore, there is a limitation concerning the estimation of PV‐based models, which may underestimate variability in PV estimation, potentially affecting cutoff estimation. Strategies such as the Bayesian bootstrap proposed by de Carvalho et al. (2013) offer potential solutions, but their translation to the cutoff estimation process remains unclear. These considerations highlight avenues for future research and refinement of methodologies in biomarker diagnostic accuracy assessment. Another exciting extension of this work would be to propose unified metrics that combine sensitivity, specificity, and ROC curves while accounting for distributional variations and covariates. Notably, Lange and Brunner (2012) defined sensitivity and specificity as specific AUCs within a nonparametric, empirical framework. In the future, this approach could be expanded to incorporate covariates, offering an alternative framework for estimating the optimal cutoff.

## Conflicts of Interest

The author declares no conflicts of interest.

## Code Availability Statement

The R codes used for the analysis in this manuscript are publicly available in the GitHub repository at [https://github.com/soutikghosal/Covariate‐wise‐optimal‐cutoff.git ]. The repository contains all the scripts and relevant documentation required to reproduce the results presented in the paper.

### Open Research Badges

This article has earned an Open Data badge for making publicly available the digitally‐shareable data necessary to reproduce the reported results. The data is available in the Supporting Information section.

This article has earned an open data badge “**Reproducible Research**” for making publicly available the code necessary to reproduce the reported results. The results reported in this article were reproduced partially due to data confidentiality issues.

## Supporting information

Supporting Information

## Data Availability

The ADNI data are publicly available online. Users can access the data sets and biosamples following an approval process. To request approval, please visit https://adni.loni.usc.edu/data‐samples/access‐data/.

## Endnotes

1 As such, the investigators within the ADNI contributed to the design and implementation of ADNI and/or provided data but did not participate in analysis or writing of this article. A complete listing of ADNI investigators can be found at: https://adni.loni.usc.edu/wp‐content/uploads/how_to_apply/ADNI_Acknowledgement_List.pdf.

## Appendix A Noted Receiver Operating Characteristic (ROC) Frameworks

### A.1 General ROC Model

The prevailing ROC curve frameworks, including the empirical, parametric (such as the binormal model, bigamma model, etc.), and nonparametric (such as kernel‐based ROC models), are all derivatives of the general ROC model framework. Within this framework, the distributions $F_{0}$ and $F_{1}$ are characterized either empirically, parametrically, or nonparametrically. Below, we briefly describe some of these widely recognized models:

- **Empirical model**: The empirical model assumes no particular form of the ROC curve, rather is characterized by the empirical CDFs estimated as
  (A1)
  $$\begin{array}{cc} F_{j}\left(x\right) & =\frac{1}{n_{j}}\sum_{i=1}^{n_{j}}I\left(Y_{ji}<x\right),\;j=0,1, \end{array}$$
  and the corresponding ROC curve can be obtained from Equation (1) and the AUC can be estimated from DeLong et al. (1988) as
  $$\begin{array}{c} AUC=\frac{1}{n_{0}n_{1}}\sum_{i=1}^{n_{0}}\sum_{j=1}^{n_{1}}\Psi \left(Y_{0i},Y_{1j}\right), \end{array}$$
  where
  $$\begin{array}{cc} & \Psi \left(a,b\right)=\left\{\begin{array}{cc} 1, & a<b\\ 0, & a>b. \end{array}\right. \end{array}$$
- **Nonparametric models**: While the empirical estimates are useful because of simplicity in structure and no requirement of model assumptions, the empirical estimates of ROC curve are not smooth. To overcome the lack of smoothness, the nonparametric models especially the Kernel‐based smooth models became more popular (Zou et al. 1997; Lloyd 1998). Based on Kernel estimators, we can specify $F_{0}$ and $F_{1}$ as
  (A2)
  $$\begin{array}{cc} F_{j}\left(x\right) & =\frac{1}{n_{j}}\sum_{i=1}^{n_{j}}\kappa \left(\frac{x-Y_{ji}}{h_{j}}\right),\\ h_{j} & =0.9\cdot min\left(SD\left(Y_{j}\right),\;IQR\left(Y_{j}\right)/1.34\right)\cdot n_{j}^{-1/5},\;j=0,1, \end{array}$$
  where κ is the kernel, $h_{j}$’s are the bandwidths, SD(x) and IQR(x) corresponds to, respectively, the standard deviation and interquartile range of x. In this article, Gaussian kernels were employed for estimating ROC curves from nonparametric models.
- **Binormal model**: Consider $Y_{0}∼N\left(\mu_{0},\sigma_{0}^{2}\right)$ and $Y_{1}∼N\left(\mu_{1},\sigma_{1}^{2}\right)$, where N(a,b) denotes normal distribution with mean a and variance b. Then according to the binormal model, we can estimate
  (A3)
  $$a=\frac{\mu_{1}-\mu_{0}}{\sigma_{1}},\;b=\frac{\sigma_{0}}{\sigma_{1}},$$
  and the binormal ROC and AUC have the closed form given by
  (A4)
  $$\begin{array}{cc} ROC(t) & =\Phi (a+b\cdot \Phi^{-1}\left(t\right)),\;t\in \left(0,1\right),\\ AUC & =\Phi \left(\frac{a}{\sqrt{1+b^{2}}}\right), \end{array}$$
  where Φ(y) corresponds to a standard normal CDF obtained at y. More on this can be found in Metz and Kronman (1980).

### A.2 PV‐Based ROC Model

Some special PV‐based ROC models are:

- **Parametric PV model**: Following the transformed normal model in Ghosal and Chen (2022), we can come up with a parametric version of a PV‐based model, where $Y_{0}∼F_{0}\left(\cdot \right)\equiv N\left(\mu_{0},\sigma_{0}^{2}\right)$ similar to the BN model, and PV can be estimated as $Z=1-\Phi \left(Y_{1};\mu_{0},\sigma_{0}\right)$. We can model Z as $\Phi^{-1}\left(Z\right)∼N\left(\mu ,\sigma \right)$, and the corresponding ROC curve and AUC can be estimated as
  (A5)
  $$\begin{array}{cc} ROC(t) & =\Phi \left(\Phi^{-1}\left(t\right);\mu ,\sigma \right),\;t\in \left(0,1\right),\\ AUC & =\int_{0}^{1}ROC\left(t\right)dt. \end{array}$$
- **Semiparametric PV model**: The PV‐based semiparametric model is based on the Dirichlet process mixture (DPM) model which models the data as a mixture of normals where the DP prior is assumed on the mixing distribution. For example, we can write $F_{0}$ in terms of DPM as
  $$\begin{array}{cc} F_{0}\left(x\right) & =\int \Phi \left(x;\mu_{0},\sigma_{0}\right)G_{0}\left(\mu_{0}\right),\\ G_{0} & ∼DP(\alpha_{0},G_{0}^{*}), \end{array}$$
  where $\alpha_{0}$ is the concentration parameter, $G_{0}^{*}\left(\mu_{0}\right)$ is the baseline distribution with appropriate normal priors. Note that, $G_{0}$ can include both $\mu_{0}$ and $\sigma_{0}$. We opt to solely incorporate the location parameter in the DP prior, guided by literature (Ghosal et al. 1999; Lijoi et al. 2005), which demonstrates that any density on the real line can be approximated utilizing a Dirichlet process with location mixtures of normals. Considering the stick‐breaking representation of the DP (Sethuraman 1994) and truncated DP process (Ishwaran and James 2002), we can write the infinite mixtures of normal representation of DPM as a finite mixture of normals given by
  $$\begin{array}{cc} F_{0}\left(x\right) & =\sum_{k=1}^{K}\pi_{0k}\Phi \left(x;\mu_{0k},\sigma_{0}\right), \end{array}$$
  where K is a finite number of clusters, say K=30, $\pi_{0}=\left(\pi_{01},\pi_{02},\text{…},\pi_{0K}\right)$ corresponds to the mixing weights correspond to $F_{0}$. Now, based on the above specification, we can estimate the PV as
  $$\begin{array}{cc} z_{j} & =1-\sum_{k=1}^{K}\pi_{0k}\Phi \left(y_{1j};\mu_{0k},\sigma_{0}\right),\;j=1,\text{…},n_{j}, \end{array}$$
  and fit the $\eta^{-1}\left(z\right)$ as a separate yet similar DPM structure as above given as before, which results in the estimation of the CDF of PV as F, that is, the ROC curve and the corresponding AUC as
  (A6)
  $$\begin{array}{cc} F(t)=ROC(t) & =\sum_{k=1}^{K}\pi_{k}\Phi \left(\eta^{-1}\left(t\right);\mu_{k},\sigma \right),\;t\in \left(0,1\right),\\ AUC & =\int_{0}^{1}ROC\left(t\right)dt, \end{array}$$
  where the interpretations of μ, σ, π are similar for the estimation of F or the ROC curve.
