# Supplementary material for: Impact of Methodological Assumptions and Covariates on the Cutoff Estimation in ROC Analysis
Source: Biom J. 2025 Apr 27;67(3):e70053. doi: 10.1002/bimj.70053 (PMC12035111; doi:10.1002/bimj.70053)
Supplement: Supplementary file 1 — Supporting Information [file BIMJ-67-e70053-s001.pdf]

Biometrical Journal **XX** (XXXX) XX, zzz–zzz / DOI: 10.1002/bimj.200100000

Supplement for "Impact of methodological assumptions and covariates on the cutoff estimation in ROC analysis"

Soutik Ghosal[*,1]

[1] Division of Biostatistics, Department of Public Health Sciences, School of Medicine, University of Virginia, Charlottesville, VA 22903

In this supplementary material accompanying the main paper, we provide technical information along with supporting tables and figures. Section A details the true values of AUC and optimal cutoffs for simulated data with and without covariates. Section B presents bias plots for simulations across all sample sizes, along with tables for low and high sample sizes. Finally, Section **??** showcases density plots of the biomarkers used in the data analyses, with different cutoff estimates obtained from various ROC methodologies overlaid on the plots.

A True cutoff

Table A.1 and Table A.2 shows the true values of AUC, cutoff estimates for different data generating mechanism under no covariate and with covariate framework respectively.

B Other simulations

This section contains figures and tables corresponding to the simulation that could not be accommodated in the main paper due to space constraints.

B.1 Without covariates

Figures B.1, B.3, and B.5 respectively shows the biases for different AUC levels, obtained from different ROC methodologies in estimating four different cutoffs under different data generating mechanism, when covariates don't impact the data generating process and the sample size is medium, i.e. $N = 100$ for both healthy and diseased biomarker. Figures B.2, B.4, and B.6 respectively shows the corresponding biases for estimating AUCs. The bias plots for cutoffs and AUCs are separated because the bias for estimating AUCs is bounded and on a different scale compared to that for estimating the cutoffs.

Tables B.3a - B.3c present the biases in estimating AUCs and different cutoffs from different ROC methodologies under different data generating mechanism and different AUC levels for no covariate framework with low sample size.

Figures B.7, B.9, and B.11 respectively shows the biases for different AUC levels, obtained from different ROC methodologies in estimating four different cutoffs under different data generating mechanism, when covariates don't impact the data generating process and the sample size is low, i.e. $N = 50$ for both healthy and diseased biomarker. Figures B.8, B.10, and B.12 respectively shows the corresponding biases for estimating AUCs.

*Corresponding author: e-mail: soutik.ghosal@virginia.edu, Phone: +1-434 924 8222

© XXXX WILEY-VCH Verlag GmbH & Co. KGaA, Weinheim

Tables B.4a - B.4c present the biases in estimating AUCs and different cutoffs from different ROC methodologies under different data generating mechanism and different AUC levels for no covariate framework with high sample size.

Figures B.13, B.15, and B.17 respectively shows the biases for different AUC levels, obtained from different ROC methodologies in estimating four different cutoffs under different data generating mechanism, when covariates don't impact the data generating process and the sample size is high, i.e. $N = 500$ for both healthy and diseased biomarker. Figures B.14, B.16, and B.18 respectively shows the corresponding biases for estimating AUCs.

B.2 With covariates

Figure B.19 shows the biases for different covariate levels, obtained from different ROC methodologies in estimating four different cutoffs under different data generating mechanism, when covariates impacts the data generating process and the sample size is medium, i.e. $N = 100$ for both healthy and diseased biomarker. Figure B.20 shows the corresponding biases for estimating AUCs.

Table B.5 presents the biases in estimating AUCs and different cutoffs from different ROC methodologies under different data generating mechanism and different covariate levels with low sample size.

Figure B.21 shows the biases for different covariate levels, obtained from different ROC methodologies in estimating four different cutoffs under different data generating mechanism, when covariates impacts the data generating process and the sample size is low, i.e. $N = 50$ for both healthy and diseased biomarker. Figure B.22 shows the corresponding biases for estimating AUCs.

Table B.6 presents the biases in estimating AUCs and different cutoffs from different ROC methodologies under different data generating mechanism and different covariate levels with high sample size.

Figure B.23 shows the biases for different covariate levels, obtained from different ROC methodologies in estimating four different cutoffs under different data generating mechanism, when covariates impacts the data generating process and the sample size is high, i.e. $N = 500$ for both healthy and diseased biomarker. Figure B.24 shows the corresponding biases for estimating AUCs.

© XXXX WILEY-VCH Verlag GmbH & Co. KGaA, Weinheim

Table A.1 True AUC and cutoff estimates under no covariate framework.

Data generating mechanism	AUC level	Method	True AUC	True Cutoff	Data generating mechanism	AUC level	Method	True AUC	True Cutoff
BN.equal	Low	J	0.556	0.100	Skewed.III	Low	J	0.564	0.061
		ER		0.100			ER		0.031
		CZ		0.100			CZ		0.031
		IU		0.100			IU		0.030
	Medium	J	0.760	0.500		Medium	J	0.753	0.108
		ER		0.500			ER		0.065
		CZ		0.500			CZ		0.077
		IU		0.500			IU		0.067
	High	J	0.961	1.250		High	J	0.924	0.216
		ER		1.250			ER		0.149
		CZ		1.250			CZ		0.199
		IU		1.250			IU		0.157
BN.unequal	Low	J	0.555	-0.636	Mixed.I	Low	J	0.572	1.415
		ER		-0.069			ER		0.658
		CZ		-0.107			CZ		0.847
		IU		0.089			IU		0.182
	Medium	J	0.779	0.325		Medium	J	0.767	1.389
		ER		0.548			ER		0.911
		CZ		0.436			CZ		1.151
		IU		0.616			IU		0.730
	High	J	0.928	1.804		High	J	0.928	1.650
		ER		1.667			ER		1.360
		CZ		1.772			CZ		1.584
		IU		1.730			IU		1.461
Skewed.I	Low	J	0.556	0.010	Mixed.II	Low	J	0.600	1.505
		ER		0.010			ER		0.864
		CZ		0.010			CZ		0.913
		IU		0.010			IU		0.783
	Medium	J	0.794	0.128		Medium	J	0.737	1.408
		ER		0.258			ER		1.065
		CZ		0.203			CZ		1.140
		IU		0.183			IU		1.208
	High	J	0.910	9.851		High	J	0.944	1.768
		ER		8.946			ER		1.634
		CZ		9.070			CZ		1.716
		IU		7.620			IU		1.768
Skewed.II	Low	J	0.556	1.105					
		ER		1.105					
		CZ		1.105					
		IU		1.105					
	Medium	J	0.794	1.430					
		ER		1.662					
		CZ		1.570					
		IU		1.532					
	High	J	0.910	5.994					
		ER		6.762					
		CZ		6.222					
		IU		6.229					

© XXXX WILEY-VCH Verlag GmbH & Co. KGaA, Weinheim

Table A.2 True AUC and cutoff estimates under covariate framework.

**Data generating mechanism**	**Covariate level**	**Method**	**True AUC**	**True cutoff**
BN	$X = 0$	J	0.638	1.250
		ER		1.250
		CZ		1.250
		IU		1.250
	$X = 1$	J	0.856	2.750
		ER		2.750
		CZ		2.750
		IU		2.750
Skewed	$X = 0$	J	0.683	7.663
		ER		6.731
		CZ		6.874
		IU		7.663
	$X = 1$	J	0.911	12.006
		ER		10.844
		CZ		11.588
		IU		13.355
Mixed	$X = 0$	J	0.609	0.949
		ER		0.405
		CZ		0.472
		IU		0.716
	$X = 1$	J	0.917	2.234
		ER		2.095
		CZ		2.186
		IU		2.424

© XXXX WILEY-VCH Verlag GmbH & Co. KGaA, Weinheim

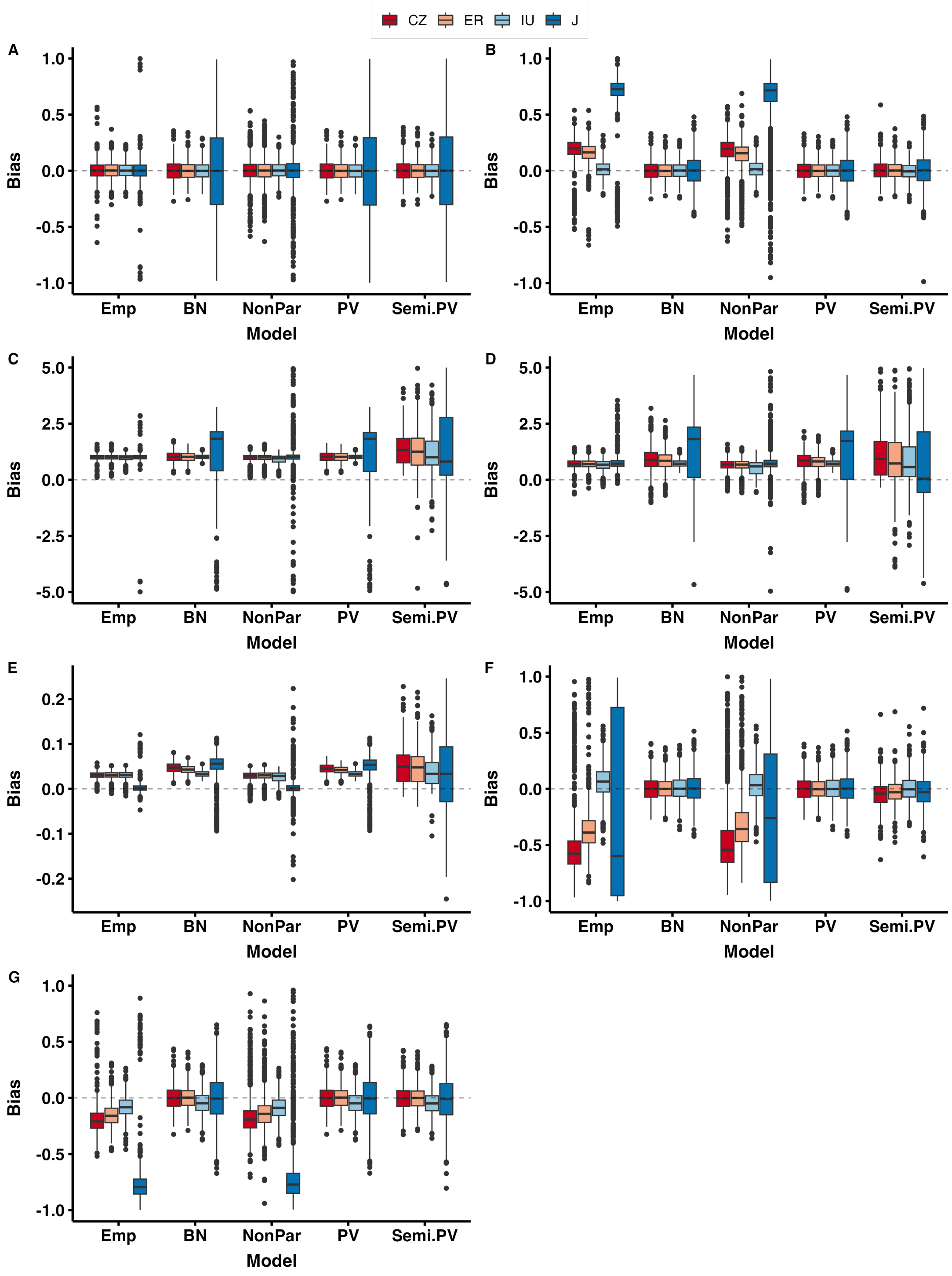

Figure B.1 Bias in estimation of optimal cutoff for medium sample size, when AUC level is low. Panels A - G respectively correspond to the simulation scenarios: BN equal, BN unequal, Skewed I, Skewed II, Skewed III, Mixed I, and Mixed II.

© XXXX WILEY-VCH Verlag GmbH & Co. KGaA, Weinheim

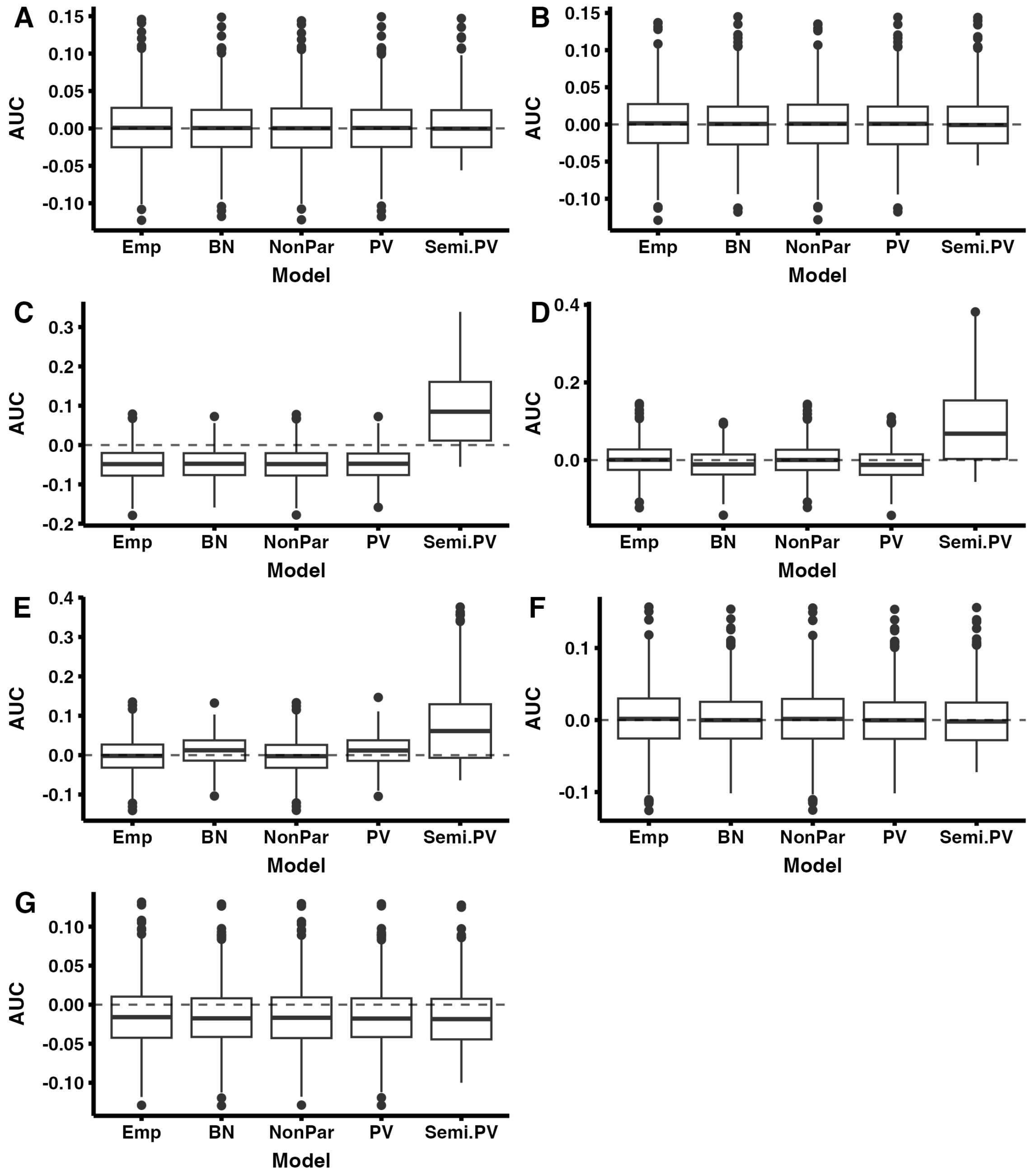

Figure B.2 Bias in estimation of AUC for medium sample size, when AUC level is low. Panels A - G respectively correspond to the simulation scenarios: BN equal, BN unequal, Skewed I, Skewed II, Skewed III, Mixed I, and Mixed II.

© XXXX WILEY-VCH Verlag GmbH & Co. KGaA, Weinheim

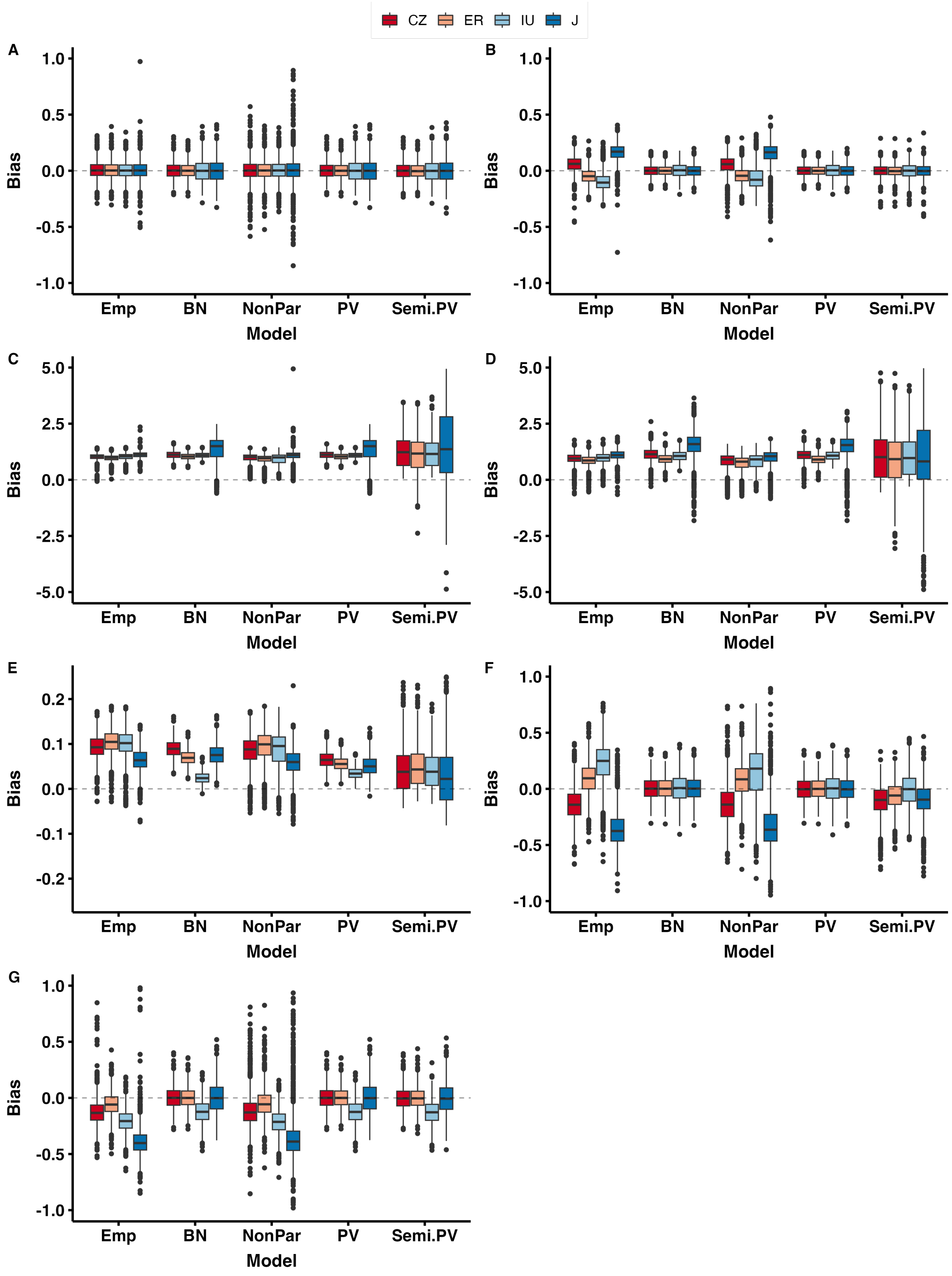

Figure B.3 Bias in estimation of optimal cutoff for medium sample size, when AUC level is medium. Panels A - G respectively correspond to the simulation scenarios: BN equal, BN unequal, Skewed I, Skewed II, Skewed III, Mixed I, and Mixed II.

© XXXX WILEY-VCH Verlag GmbH & Co. KGaA, Weinheim

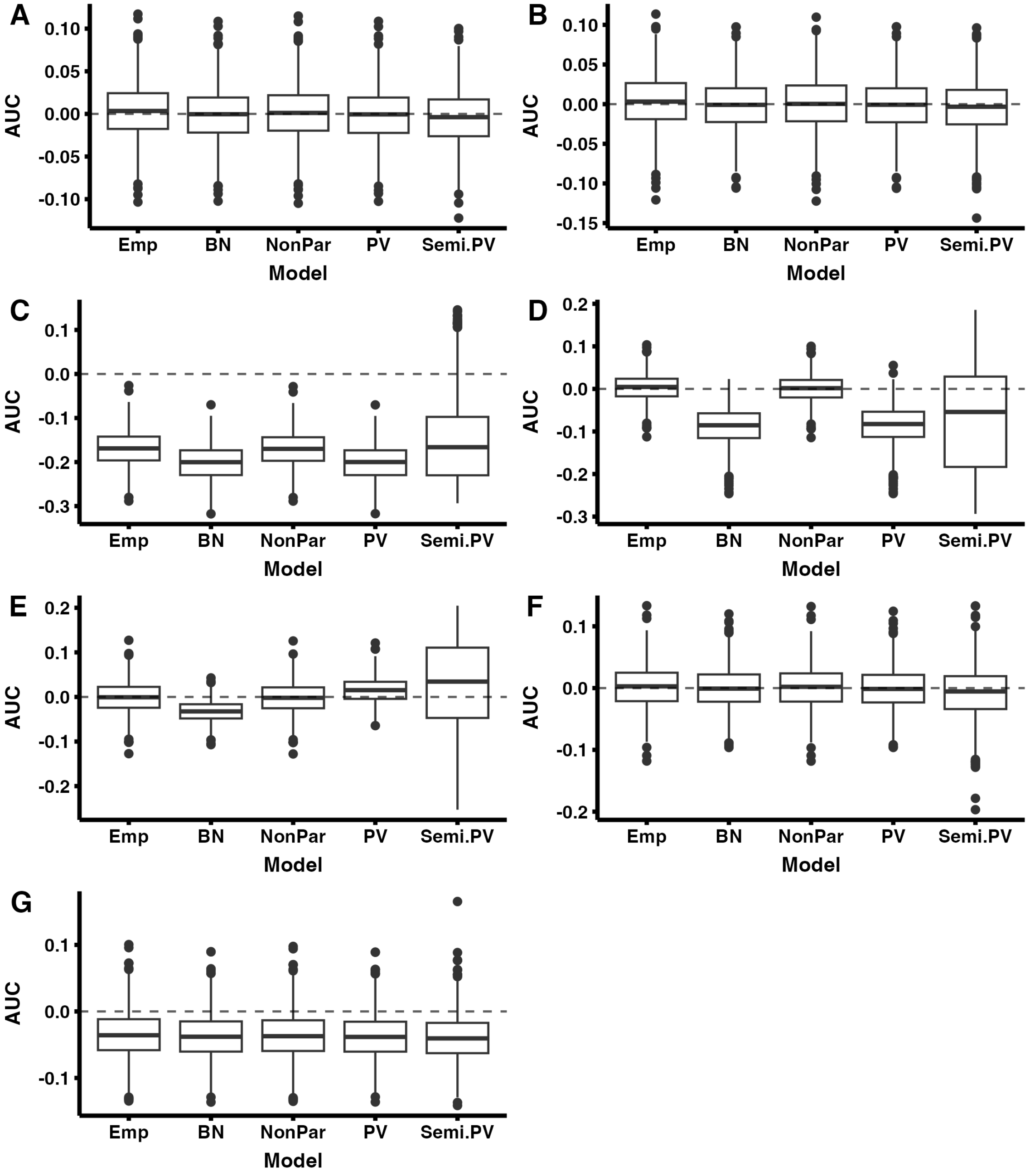

Figure B.4 Bias in estimation of AUC for medium sample size, when AUC level is medium. Panels A - G respectively correspond to the simulation scenarios: BN equal, BN unequal, Skewed I, Skewed II, Skewed III, Mixed I, and Mixed II.

© XXXX WILEY-VCH Verlag GmbH & Co. KGaA, Weinheim

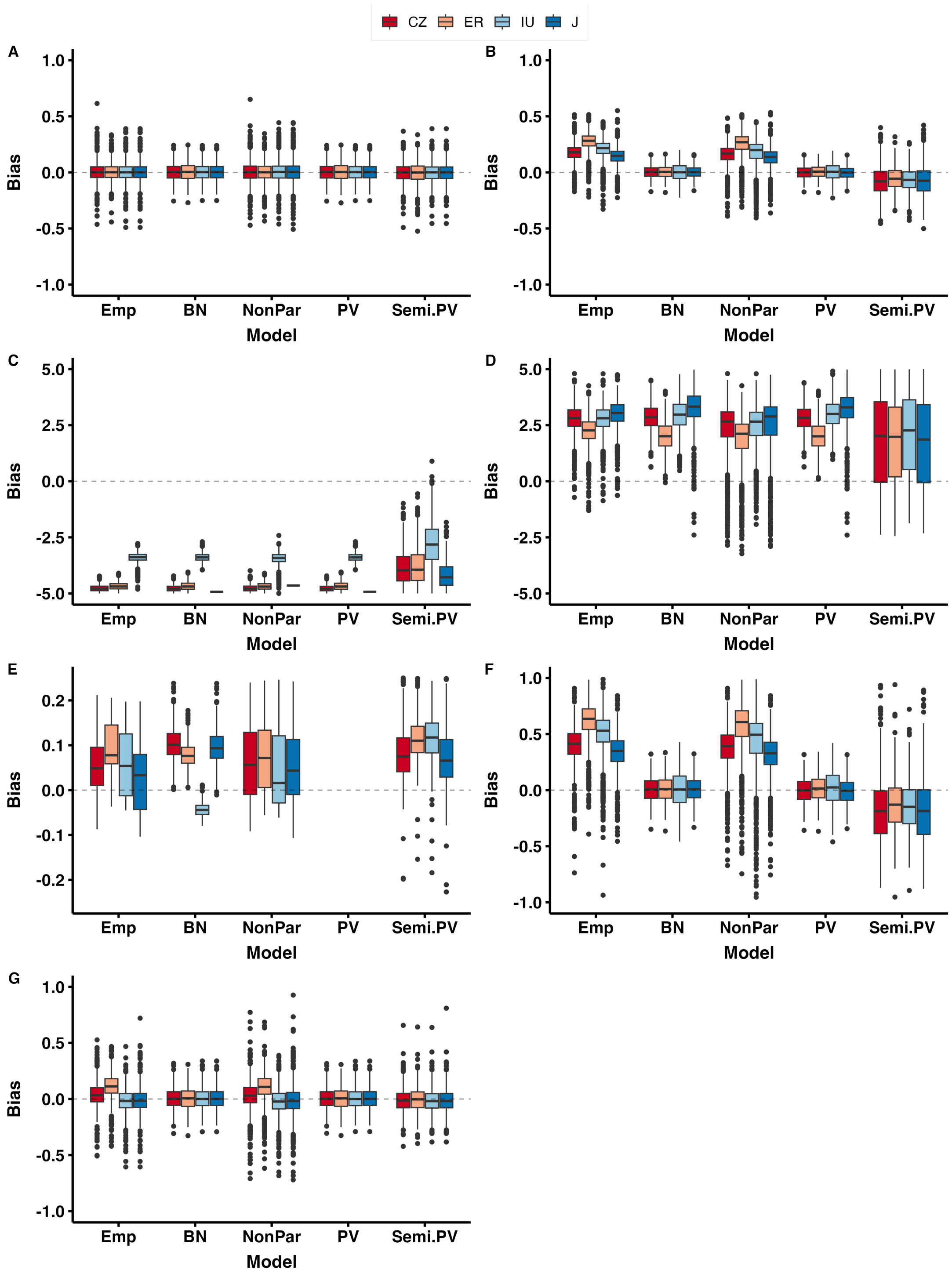

Figure B.5 Bias in estimation of optimal cutoff for medium sample size, when AUC level is high. Panels A - G respectively correspond to the simulation scenarios: BN equal, BN unequal, Skewed I, Skewed II, Skewed III, Mixed I, and Mixed II.

© XXXX WILEY-VCH Verlag GmbH & Co. KGaA, Weinheim

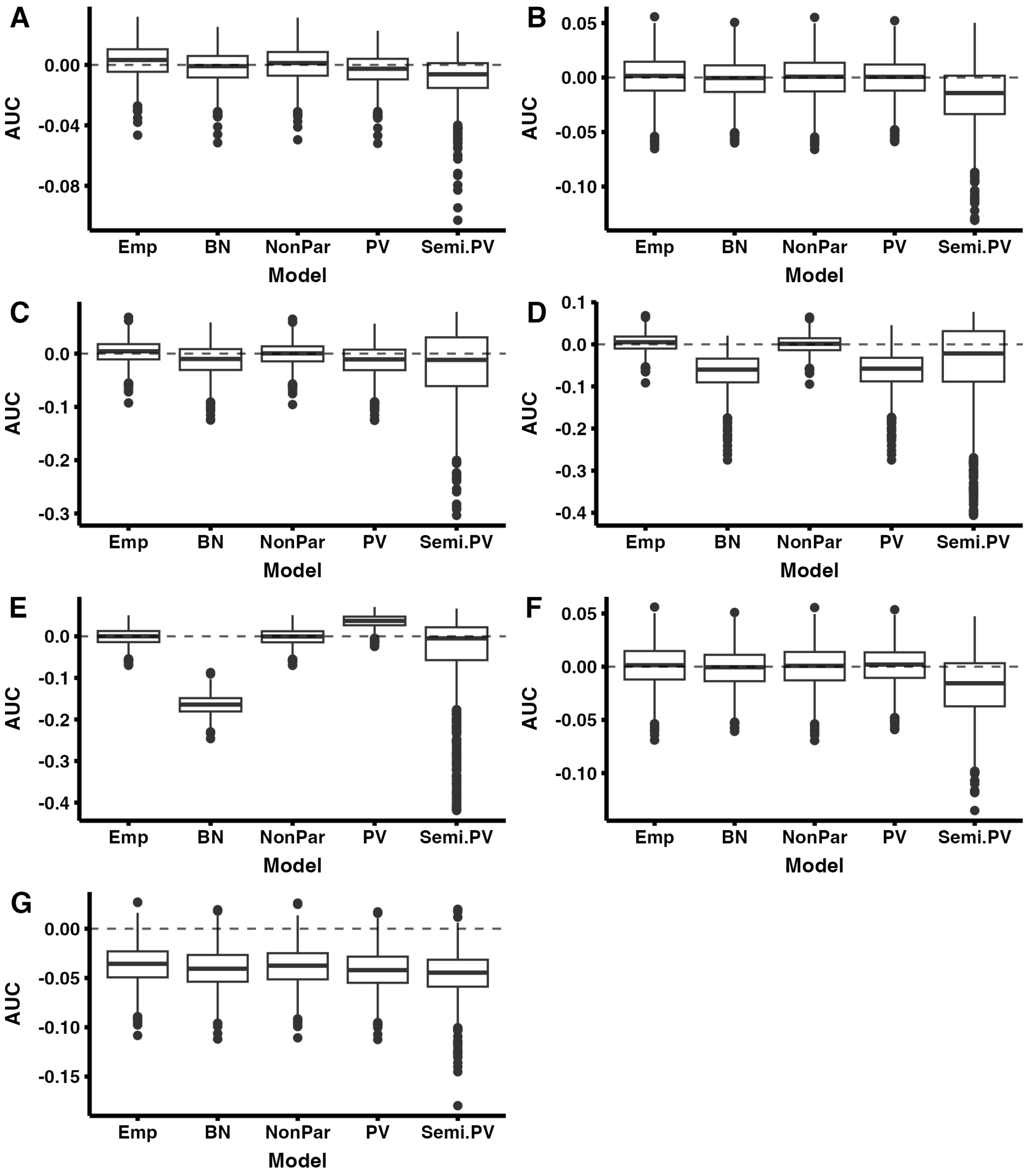

Figure B.6 Bias in estimation of AUC for medium sample size, when AUC level is high. Panels A - G respectively correspond to the simulation scenarios: BN equal, BN unequal, Skewed I, Skewed II, Skewed III, Mixed I, and Mixed II.

© XXXX WILEY-VCH Verlag GmbH & Co. KGaA, Weinheim

Table B.3a Biases of estimating AUC and optimal cutoffs for different fitting models and low AUC level for low sample size.

Data generating mechanism	Fitting model	Median ± IQR				
		AUC	J	ER	CZ	IU
BN equal	Emp	0.004 ± 0.08	0 ± 0.139	0 ± 0.138	0 ± 0.138	-0.001 ± 0.134
	BN	0.001 ± 0.077	0.016 ± 0.813	0.001 ± 0.165	0.002 ± 0.178	0.004 ± 0.144
	NonPar	0.003 ± 0.079	0.001 ± 0.214	-0.001 ± 0.175	0.001 ± 0.178	-0.003 ± 0.147
	PV	0.001 ± 0.077	0.021 ± 0.813	0.001 ± 0.166	0.002 ± 0.177	0.004 ± 0.143
	Semi.PV	0.002 ± 0.065	0.017 ± 0.818	0 ± 0.167	0 ± 0.178	0.001 ± 0.158
BN unequal	Emp	0.002 ± 0.086	0.73 ± 0.141	0.164 ± 0.139	0.203 ± 0.138	0.01 ± 0.133
	BN	0 ± 0.078	-0.002 ± 0.297	0.001 ± 0.147	0.003 ± 0.161	-0.003 ± 0.141
	NonPar	0.001 ± 0.084	0.716 ± 0.216	0.152 ± 0.173	0.193 ± 0.17	0.021 ± 0.143
	PV	0 ± 0.079	-0.001 ± 0.298	0.001 ± 0.146	0.004 ± 0.161	-0.004 ± 0.14
	Semi.PV	0.001 ± 0.067	-0.001 ± 0.308	0.004 ± 0.149	0.005 ± 0.161	-0.025 ± 0.141
Skewed I	Emp	-0.053 ± 0.079	1.005 ± 0.212	1 ± 0.211	1 ± 0.209	0.991 ± 0.217
	BN	-0.046 ± 0.072	1.545 ± 2.981	1.017 ± 0.373	1.028 ± 0.389	1.016 ± 0.21
	NonPar	-0.053 ± 0.078	1.004 ± 0.265	0.986 ± 0.235	0.984 ± 0.232	0.905 ± 0.366
	PV	-0.046 ± 0.072	1.534 ± 2.954	1.014 ± 0.374	1.023 ± 0.382	1.015 ± 0.208
	Semi.PV	0.064 ± 0.138	0.939 ± 2.628	1.152 ± 1.061	1.267 ± 0.982	0.995 ± 0.904
Skewed II	Emp	0.003 ± 0.08	0.698 ± 0.343	0.689 ± 0.334	0.691 ± 0.333	0.677 ± 0.342
	BN	-0.008 ± 0.078	1.599 ± 3.009	0.807 ± 0.617	0.874 ± 0.74	0.691 ± 0.309
	NonPar	0.003 ± 0.079	0.686 ± 0.408	0.654 ± 0.38	0.664 ± 0.364	0.552 ± 0.518
	PV	-0.01 ± 0.079	1.56 ± 2.907	0.781 ± 0.568	0.845 ± 0.669	0.689 ± 0.296
	Semi.PV	0.064 ± 0.158	0.432 ± 3.454	0.581 ± 1.246	0.755 ± 1.36	0.517 ± 1.074
Skewed III	Emp	0.004 ± 0.076	0.002 ± 0.012	0.032 ± 0.013	0.031 ± 0.012	0.032 ± 0.013
	BN	0.016 ± 0.068	0.05 ± 0.032	0.042 ± 0.017	0.046 ± 0.021	0.032 ± 0.013
	NonPar	0.003 ± 0.075	0.002 ± 0.015	0.03 ± 0.015	0.03 ± 0.015	0.027 ± 0.021
	PV	0.016 ± 0.069	0.049 ± 0.03	0.041 ± 0.017	0.045 ± 0.02	0.032 ± 0.013
	Semi.PV	0.064 ± 0.149	0.014 ± 0.106	0.042 ± 0.052	0.044 ± 0.055	0.035 ± 0.043
Mixed I	Emp	0.002 ± 0.082	-1.143 ± 0.273	-0.388 ± 0.267	-0.576 ± 0.27	0.067 ± 0.257
	BN	0 ± 0.075	0.013 ± 0.267	-0.004 ± 0.182	-0.005 ± 0.206	-0.004 ± 0.206
	NonPar	0.002 ± 0.081	-1.068 ± 0.616	-0.35 ± 0.349	-0.534 ± 0.364	0.023 ± 0.256
	PV	0 ± 0.075	0.013 ± 0.271	-0.005 ± 0.182	-0.007 ± 0.205	-0.004 ± 0.204
	Semi.PV	-0.003 ± 0.07	-0.016 ± 0.279	-0.032 ± 0.187	-0.056 ± 0.199	-0.001 ± 0.202
Mixed II	Emp	-0.013 ± 0.079	-0.801 ± 0.186	-0.159 ± 0.181	-0.21 ± 0.181	-0.084 ± 0.176
	BN	-0.018 ± 0.075	0.002 ± 0.397	-0.002 ± 0.195	0.002 ± 0.211	-0.051 ± 0.179
	NonPar	-0.014 ± 0.078	-0.781 ± 0.287	-0.151 ± 0.225	-0.2 ± 0.234	-0.093 ± 0.192
	PV	-0.018 ± 0.074	0.002 ± 0.398	-0.003 ± 0.194	0 ± 0.212	-0.053 ± 0.18
	Semi.PV	-0.018 ± 0.071	-0.012 ± 0.405	-0.008 ± 0.2	-0.01 ± 0.212	-0.051 ± 0.178

© XXXX WILEY-VCH Verlag GmbH & Co. KGaA, Weinheim

Table B.3b Biases of estimating AUC and optimal cutoffs for different fitting models and medium AUC level for low sample size.

**Data generating mechanism**	**Fitting model**	**Median ± IQR**				
		AUC	J	ER	CZ	IU
BN equal	Emp	0.007 ± 0.065	-0.001 ± 0.136	-0.001 ± 0.136	0 ± 0.137	-0.001 ± 0.133
	BN	-0.002 ± 0.065	0.001 ± 0.204	-0.002 ± 0.128	0 ± 0.143	0.001 ± 0.183
	NonPar	0.003 ± 0.065	-0.001 ± 0.18	0 ± 0.161	0 ± 0.165	0.001 ± 0.156
	PV	-0.002 ± 0.065	0.001 ± 0.204	-0.001 ± 0.128	0 ± 0.143	0.001 ± 0.184
	Semi.PV	-0.009 ± 0.065	-0.001 ± 0.207	-0.006 ± 0.133	-0.002 ± 0.146	-0.001 ± 0.182
BN unequal	Emp	0.007 ± 0.07	0.166 ± 0.124	-0.052 ± 0.121	0.058 ± 0.125	-0.112 ± 0.128
	BN	-0.001 ± 0.066	-0.004 ± 0.111	-0.006 ± 0.09	-0.004 ± 0.092	-0.002 ± 0.128
	NonPar	0.002 ± 0.068	0.154 ± 0.155	-0.043 ± 0.136	0.057 ± 0.141	-0.067 ± 0.174
	PV	-0.001 ± 0.066	-0.005 ± 0.111	-0.006 ± 0.091	-0.004 ± 0.092	-0.003 ± 0.127
	Semi.PV	-0.005 ± 0.066	-0.006 ± 0.111	-0.01 ± 0.091	-0.004 ± 0.093	-0.01 ± 0.13
Skewed I	Emp	-0.171 ± 0.078	1.105 ± 0.201	0.972 ± 0.201	1.029 ± 0.201	1.044 ± 0.21
	BN	-0.199 ± 0.077	1.51 ± 1.021	1.025 ± 0.262	1.12 ± 0.306	1.078 ± 0.214
	NonPar	-0.173 ± 0.077	1.081 ± 0.259	0.933 ± 0.269	0.991 ± 0.266	0.945 ± 0.372
	PV	-0.199 ± 0.076	1.51 ± 1.021	1.024 ± 0.263	1.12 ± 0.306	1.076 ± 0.214
	Semi.PV	-0.18 ± 0.132	1.458 ± 2.34	1.104 ± 0.985	1.209 ± 0.964	1.04 ± 0.892
Skewed II	Emp	0.007 ± 0.064	1.107 ± 0.351	0.872 ± 0.351	0.965 ± 0.351	0.992 ± 0.357
	BN	-0.081 ± 0.077	1.517 ± 0.742	0.873 ± 0.37	1.088 ± 0.434	0.989 ± 0.412
	NonPar	0.002 ± 0.063	1.013 ± 0.575	0.772 ± 0.56	0.868 ± 0.591	0.849 ± 0.595
	PV	-0.076 ± 0.083	1.468 ± 0.718	0.859 ± 0.364	1.057 ± 0.418	1.002 ± 0.397
	Semi.PV	-0.041 ± 0.179	0.771 ± 1.924	0.685 ± 1.222	0.776 ± 1.356	0.754 ± 1.152
Skewed III	Emp	0.004 ± 0.064	0.067 ± 0.042	0.109 ± 0.043	0.097 ± 0.043	0.106 ± 0.044
	BN	-0.031 ± 0.043	0.071 ± 0.042	0.067 ± 0.03	0.086 ± 0.036	0.022 ± 0.024
	NonPar	0.002 ± 0.062	0.058 ± 0.046	0.097 ± 0.054	0.086 ± 0.05	0.092 ± 0.064
	PV	0.016 ± 0.055	0.047 ± 0.041	0.053 ± 0.029	0.062 ± 0.033	0.032 ± 0.023
	Semi.PV	0.085 ± 0.134	0.007 ± 0.074	0.035 ± 0.054	0.029 ± 0.063	0.034 ± 0.05
Mixed I	Emp	0.006 ± 0.065	-0.379 ± 0.258	0.092 ± 0.254	-0.145 ± 0.259	0.259 ± 0.256
	BN	-0.002 ± 0.063	0.008 ± 0.226	-0.003 ± 0.18	-0.005 ± 0.189	-0.003 ± 0.223
	NonPar	0.004 ± 0.064	-0.366 ± 0.308	0.076 ± 0.292	-0.137 ± 0.297	0.157 ± 0.358
	PV	-0.002 ± 0.064	0.002 ± 0.229	-0.006 ± 0.177	-0.01 ± 0.191	-0.007 ± 0.224
	Semi.PV	-0.011 ± 0.07	-0.077 ± 0.243	-0.062 ± 0.197	-0.102 ± 0.204	-0.019 ± 0.251
Mixed II	Emp	-0.033 ± 0.071	-0.405 ± 0.179	-0.064 ± 0.175	-0.137 ± 0.177	-0.208 ± 0.176
	BN	-0.04 ± 0.071	0.003 ± 0.261	-0.006 ± 0.174	-0.002 ± 0.186	-0.137 ± 0.185
	NonPar	-0.035 ± 0.071	-0.39 ± 0.263	-0.056 ± 0.218	-0.131 ± 0.225	-0.225 ± 0.195
	PV	-0.04 ± 0.071	0.004 ± 0.26	-0.006 ± 0.174	-0.002 ± 0.186	-0.137 ± 0.186
	Semi.PV	-0.045 ± 0.068	-0.006 ± 0.26	-0.014 ± 0.172	-0.012 ± 0.191	-0.147 ± 0.195

© XXXX WILEY-VCH Verlag GmbH & Co. KGaA, Weinheim

Table B.3c Biases of estimating AUC and optimal cutoffs for different fitting models and high AUC level for low sample size.

Data generating mechanism	Fitting model	Median ± IQR				
		AUC	J	ER	CZ	IU
BN equal	Emp	0.006 ± 0.021	-0.003 ± 0.136	-0.002 ± 0.135	-0.003 ± 0.136	-0.003 ± 0.136
	BN	-0.001 ± 0.022	-0.003 ± 0.141	-0.002 ± 0.159	-0.003 ± 0.143	-0.003 ± 0.141
	NonPar	0.002 ± 0.023	0 ± 0.153	-0.001 ± 0.144	0 ± 0.147	-0.001 ± 0.151
	PV	-0.003 ± 0.021	-0.004 ± 0.141	-0.002 ± 0.16	-0.004 ± 0.145	-0.004 ± 0.141
	Semi.PV	-0.011 ± 0.025	-0.008 ± 0.148	-0.008 ± 0.169	-0.01 ± 0.15	-0.008 ± 0.148
BN unequal	Emp	0.004 ± 0.037	0.147 ± 0.119	0.281 ± 0.121	0.178 ± 0.121	0.216 ± 0.123
	BN	-0.002 ± 0.035	0.003 ± 0.103	0 ± 0.106	0.001 ± 0.103	-0.004 ± 0.142
	NonPar	0.003 ± 0.037	0.134 ± 0.137	0.263 ± 0.151	0.165 ± 0.141	0.187 ± 0.174
	PV	-0.001 ± 0.036	-0.002 ± 0.106	0.002 ± 0.108	-0.003 ± 0.103	-0.001 ± 0.136
	Semi.PV	-0.013 ± 0.046	-0.063 ± 0.138	-0.033 ± 0.144	-0.062 ± 0.143	-0.032 ± 0.161
Skewed I	Emp	0.009 ± 0.042	-5.616 ± 0.329	-4.711 ± 0.33	-4.835 ± 0.33	-3.384 ± 0.328
	BN	-0.01 ± 0.055	-5.663 ± 0.365	-4.767 ± 0.443	-4.876 ± 0.364	-3.423 ± 0.342
	NonPar	0.002 ± 0.042	-5.677 ± 0.423	-4.769 ± 0.414	-4.892 ± 0.419	-3.441 ± 0.405
	PV	-0.011 ± 0.054	-5.663 ± 0.364	-4.768 ± 0.445	-4.875 ± 0.36	-3.422 ± 0.346
	Semi.PV	-0.023 ± 0.092	-5.386 ± 1.584	-4.52 ± 1.246	-4.619 ± 1.419	-3.129 ± 1.323
Skewed II	Emp	0.01 ± 0.042	3.08 ± 0.902	2.305 ± 0.903	2.846 ± 0.902	2.845 ± 0.901
	BN	-0.056 ± 0.067	3.137 ± 1.293	1.886 ± 1.237	2.713 ± 1.066	2.724 ± 1.225
	NonPar	0.003 ± 0.042	2.713 ± 1.622	1.937 ± 1.462	2.493 ± 1.57	2.462 ± 1.424
	PV	-0.051 ± 0.071	3.073 ± 1.293	1.874 ± 1.231	2.686 ± 1.057	2.773 ± 1.155
	Semi.PV	-0.02 ± 0.123	3.022 ± 4.869	2.479 ± 4.274	3.088 ± 4.483	3.035 ± 4.2
Skewed III	Emp	0.004 ± 0.041	1.549 ± 0.491	1.616 ± 0.491	1.566 ± 0.491	1.607 ± 0.491
	BN	-0.16 ± 0.042	0.132 ± 0.074	0.103 ± 0.051	0.138 ± 0.071	-0.032 ± 0.027
	NonPar	0.003 ± 0.041	1.368 ± 0.693	1.435 ± 0.691	1.384 ± 0.693	1.423 ± 0.697
	PV	0.038 ± 0.03	1.567 ± 0.464	1.633 ± 0.464	1.583 ± 0.464	1.625 ± 0.464
	Semi.PV	-0.019 ± 0.054	0.199 ± 0.362	0.215 ± 0.242	0.197 ± 0.317	0.231 ± 0.196
Mixed I	Emp	0.004 ± 0.038	0.352 ± 0.257	0.64 ± 0.257	0.418 ± 0.256	0.538 ± 0.262
	BN	-0.002 ± 0.035	0.007 ± 0.212	0.002 ± 0.214	0.001 ± 0.213	-0.005 ± 0.301
	NonPar	0.003 ± 0.038	0.313 ± 0.282	0.589 ± 0.31	0.376 ± 0.287	0.47 ± 0.347
	PV	0.001 ± 0.036	-0.008 ± 0.221	0.006 ± 0.219	-0.008 ± 0.215	0.01 ± 0.286
	Semi.PV	-0.014 ± 0.048	-0.144 ± 0.309	-0.081 ± 0.297	-0.145 ± 0.305	-0.069 ± 0.341
Mixed II	Emp	-0.033 ± 0.041	-0.015 ± 0.177	0.116 ± 0.178	0.036 ± 0.178	-0.018 ± 0.178
	BN	-0.041 ± 0.04	-0.004 ± 0.175	-0.005 ± 0.184	-0.003 ± 0.17	-0.011 ± 0.173
	NonPar	-0.037 ± 0.041	-0.022 ± 0.217	0.105 ± 0.208	0.028 ± 0.217	-0.03 ± 0.214
	PV	-0.043 ± 0.039	-0.004 ± 0.176	-0.006 ± 0.183	-0.005 ± 0.171	-0.012 ± 0.172
	Semi.PV	-0.05 ± 0.043	-0.024 ± 0.184	-0.018 ± 0.196	-0.026 ± 0.186	-0.03 ± 0.189

© XXXX WILEY-VCH Verlag GmbH & Co. KGaA, Weinheim

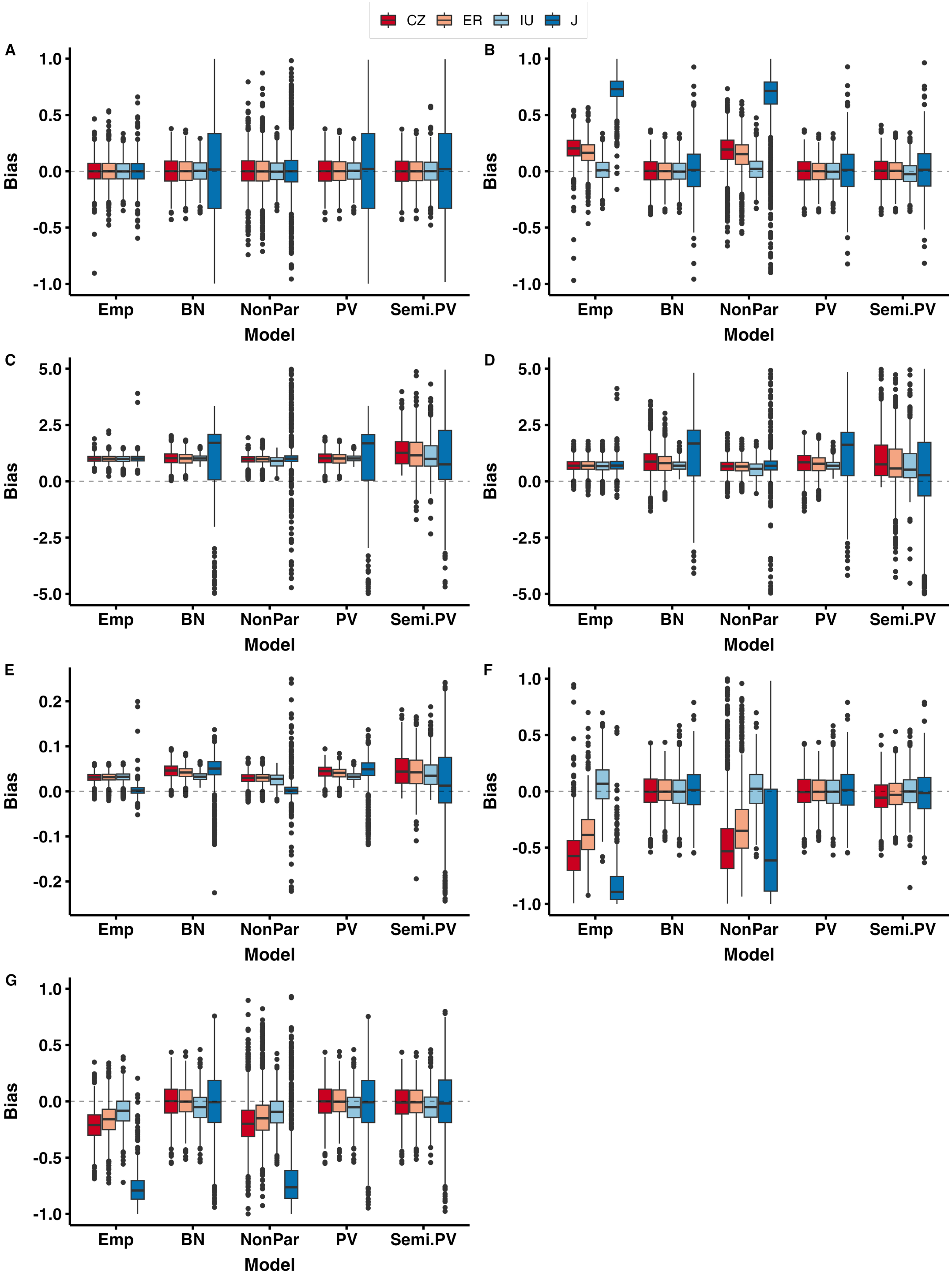

Figure B.7 Bias in estimation of optimal cutoff for low sample size, when AUC level is low. Panels A - G respectively correspond to the simulation scenarios: BN equal, BN unequal, Skewed I, Skewed II, Skewed III, Mixed I, and Mixed II.

© XXXX WILEY-VCH Verlag GmbH & Co. KGaA, Weinheim

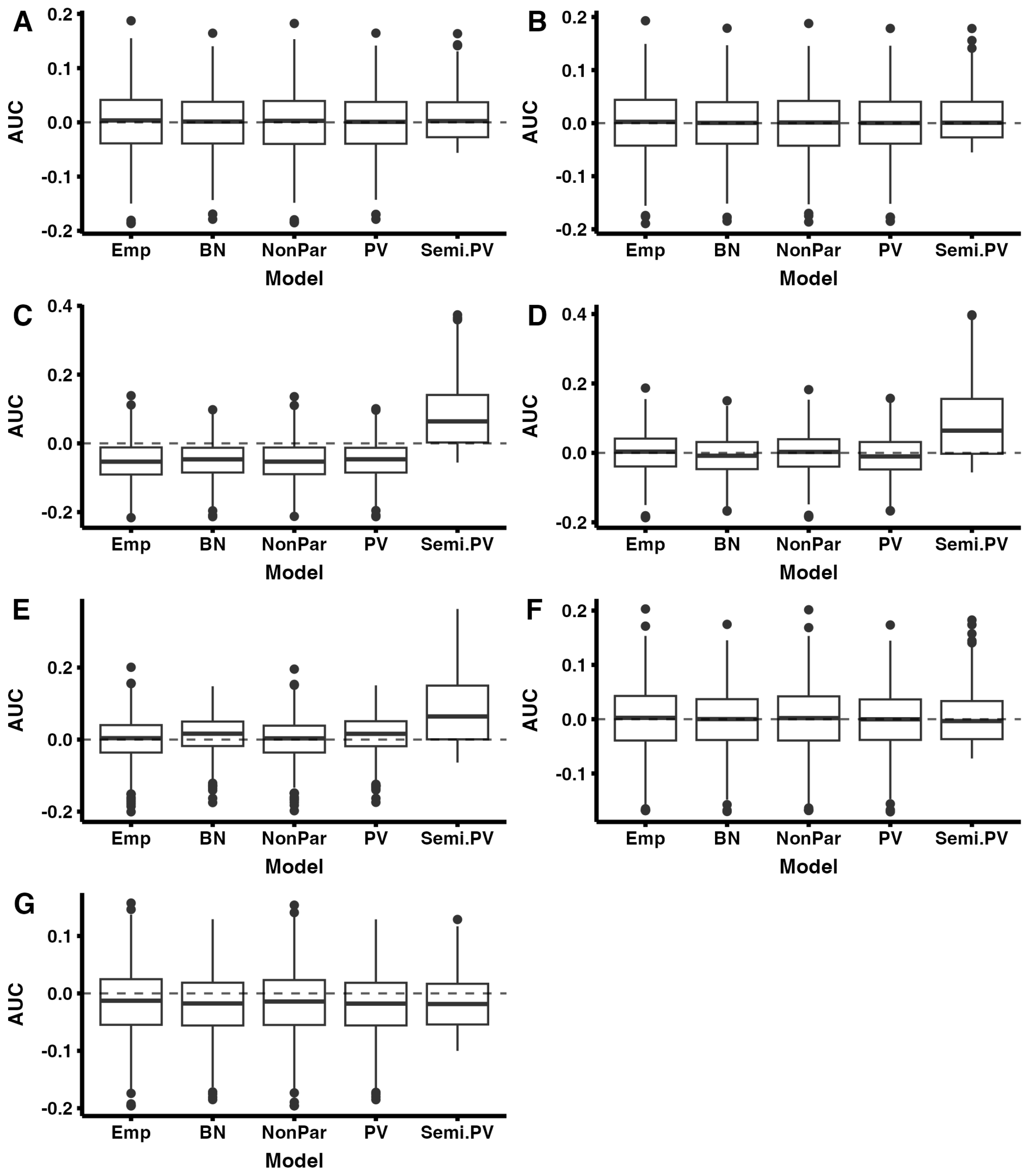

Figure B.8 Bias in estimation of AUC for low sample size, when AUC level is low. Panels A - G respectively correspond to the simulation scenarios: BN equal, BN unequal, Skewed I, Skewed II, Skewed III, Mixed I, and Mixed II.

© XXXX WILEY-VCH Verlag GmbH & Co. KGaA, Weinheim

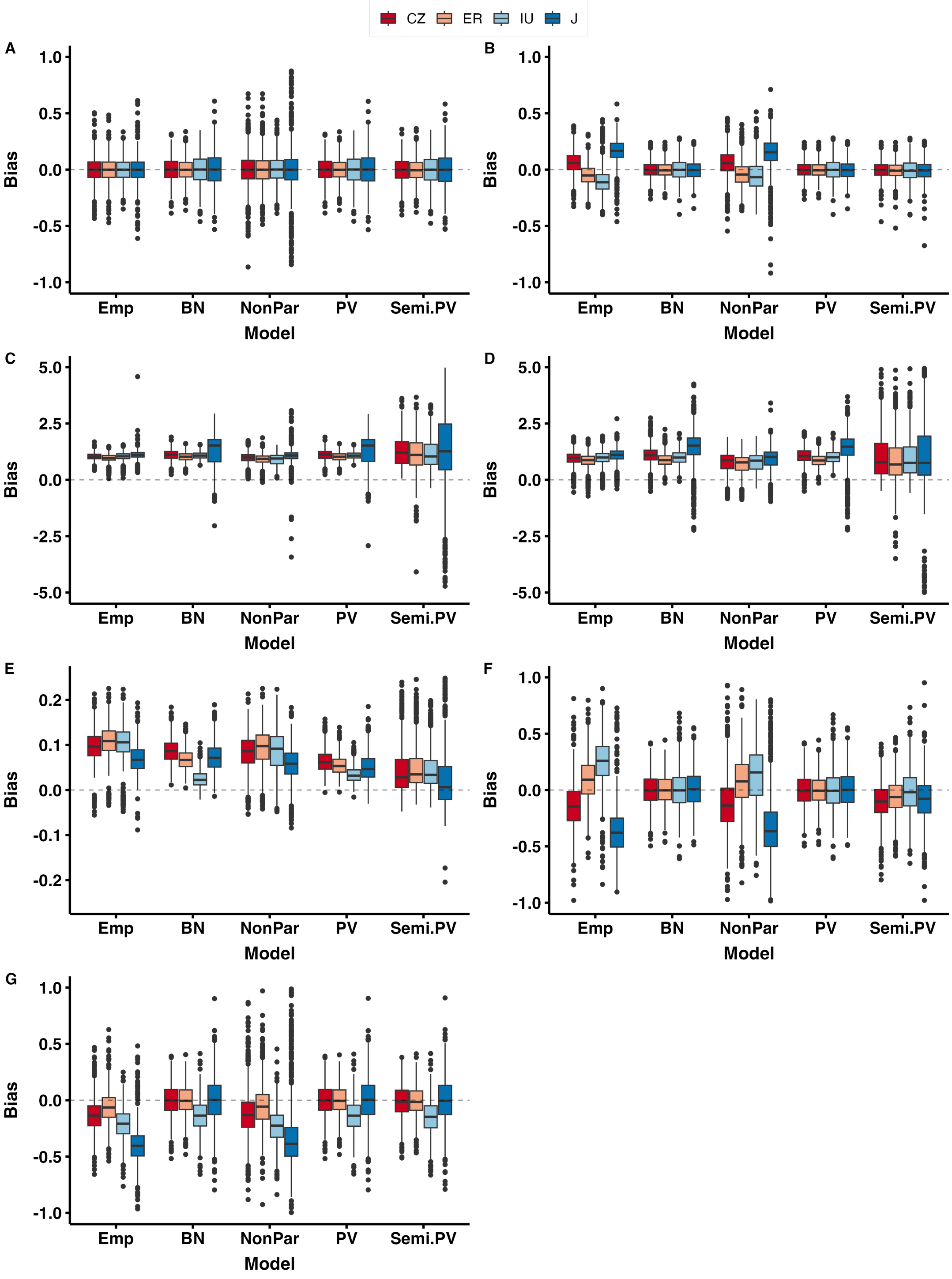

Figure B.9 Bias in estimation of optimal cutoff for low sample size, when AUC level is medium. Panels A - G respectively correspond to the simulation scenarios: BN equal, BN unequal, Skewed I, Skewed II, Skewed III, Mixed I, and Mixed II.

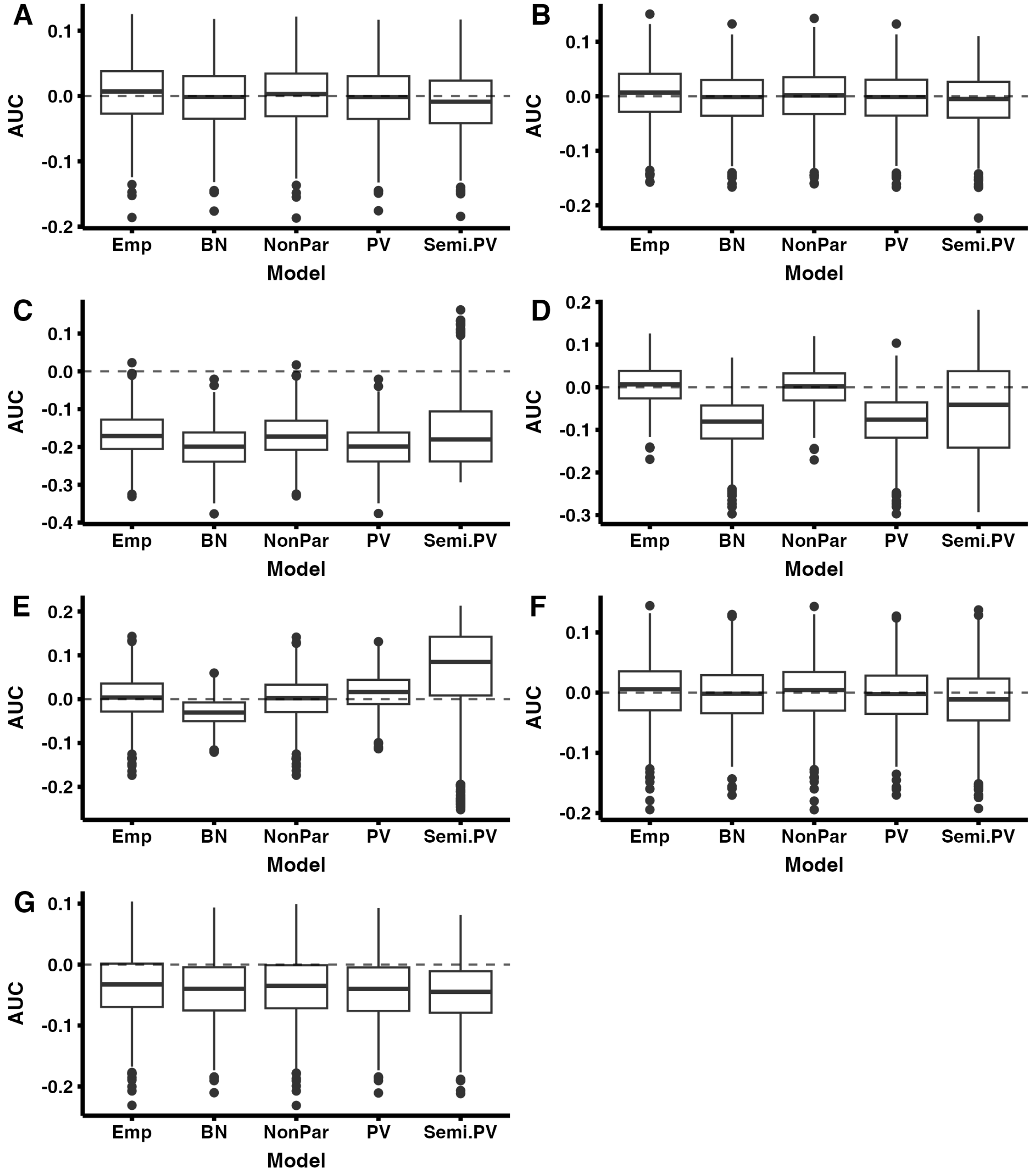

Figure B.10 Bias in estimation of AUC for low sample size, when AUC level is medium. Panels A - G respectively correspond to the simulation scenarios: BN equal, BN unequal, Skewed I, Skewed II, Skewed III, Mixed I, and Mixed II.

© XXXX WILEY-VCH Verlag GmbH & Co. KGaA, Weinheim

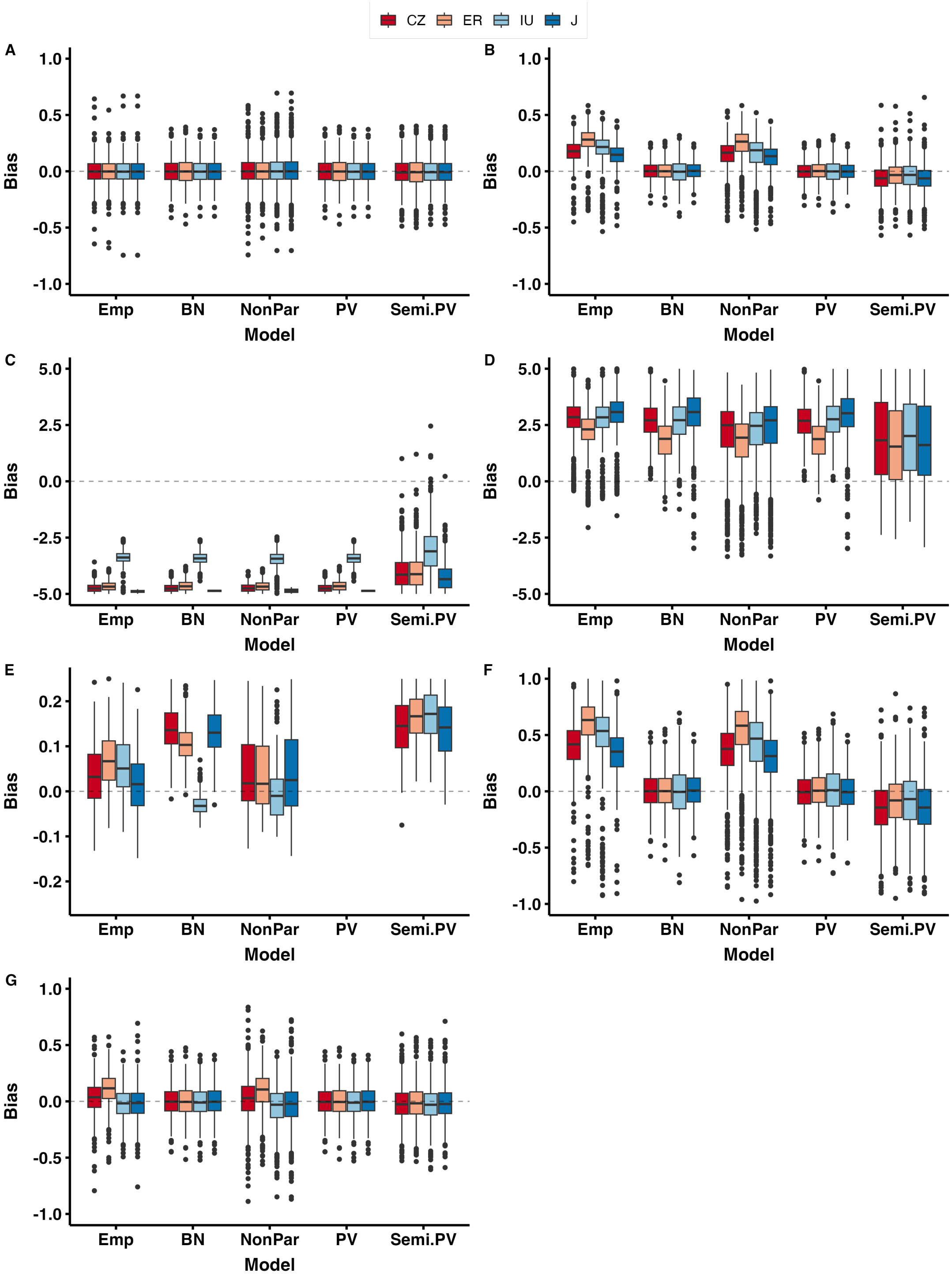

Figure B.11 Bias in estimation of optimal cutoff for low sample size, when AUC level is high. Panels A - G respectively correspond to the simulation scenarios: BN equal, BN unequal, Skewed I, Skewed II, Skewed III, Mixed I, and Mixed II.

© XXXX WILEY-VCH Verlag GmbH & Co. KGaA, Weinheim

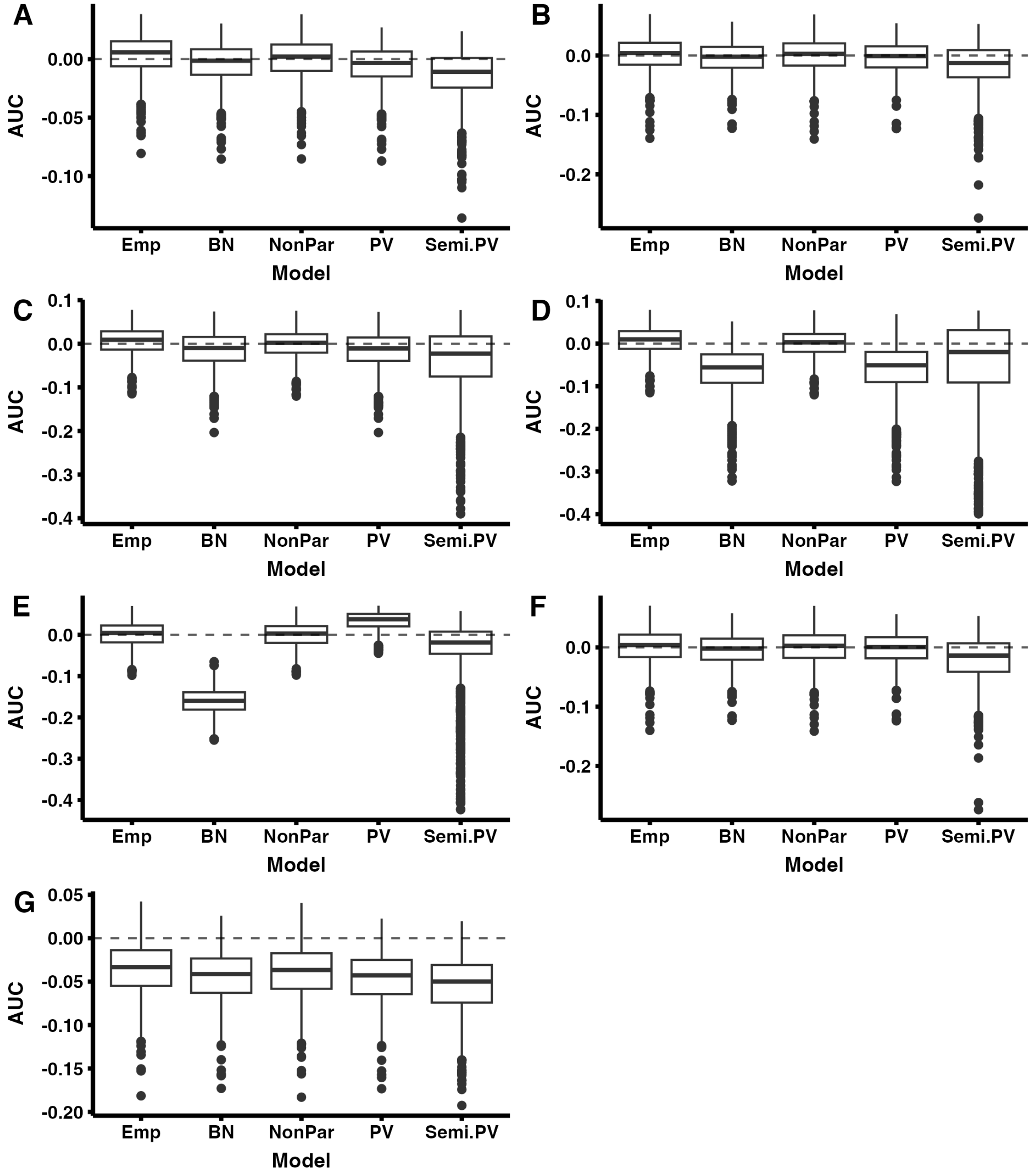

Figure B.12 Bias in estimation of AUC for low sample size, when AUC level is high. Panels A - G respectively correspond to the simulation scenarios: BN equal, BN unequal, Skewed I, Skewed II, Skewed III, Mixed I, and Mixed II.

© XXXX WILEY-VCH Verlag GmbH & Co. KGaA, Weinheim

Table B.4a Biases of estimating AUC and optimal cutoffs for different fitting models and low AUC level for high sample size.

**Data generating mechanism**	**Fitting model**	**Median ± IQR**				
		AUC	J	ER	CZ	IU
BN equal	Emp	0 ± 0.025	0.002 ± 0.07	0.001 ± 0.06	0.002 ± 0.063	0.001 ± 0.047
	BN	0 ± 0.024	0.004 ± 0.298	-0.001 ± 0.052	-0.001 ± 0.055	0.001 ± 0.073
	NonPar	0 ± 0.025	0 ± 0.081	0.003 ± 0.063	0.002 ± 0.066	0.001 ± 0.049
	PV	0 ± 0.024	0.006 ± 0.299	-0.001 ± 0.053	-0.001 ± 0.055	0.001 ± 0.073
	Semi.PV	-0.001 ± 0.025	0.005 ± 0.297	-0.002 ± 0.055	-0.003 ± 0.058	0 ± 0.075
BN unequal	Emp	0 ± 0.024	0.716 ± 0.879	0.154 ± 0.113	0.193 ± 0.108	0.015 ± 0.048
	BN	0 ± 0.023	-0.002 ± 0.092	-0.002 ± 0.049	-0.001 ± 0.053	0.002 ± 0.045
	NonPar	0 ± 0.024	0.704 ± 0.801	0.15 ± 0.126	0.187 ± 0.129	0.016 ± 0.049
	PV	0 ± 0.023	-0.001 ± 0.092	-0.002 ± 0.049	-0.001 ± 0.052	0.002 ± 0.045
	Semi.PV	-0.001 ± 0.024	-0.001 ± 0.096	-0.001 ± 0.05	0 ± 0.054	0.002 ± 0.047
Skewed I	Emp	-0.05 ± 0.024	1.007 ± 0.107	0.975 ± 0.283	0.975 ± 0.227	0.764 ± 0.532
	BN	-0.048 ± 0.023	1.819 ± 2.505	1.038 ± 0.146	1.04 ± 0.151	1.019 ± 0.065
	NonPar	-0.05 ± 0.024	1.007 ± 0.124	0.967 ± 0.303	0.969 ± 0.258	0.737 ± 0.526
	PV	-0.048 ± 0.023	1.785 ± 2.502	1.034 ± 0.141	1.036 ± 0.145	1.018 ± 0.066
	Semi.PV	0.058 ± 0.183	1.308 ± 4.037	1.046 ± 1.083	1.002 ± 0.817	1.069 ± 1.197
Skewed II	Emp	0 ± 0.025	0.715 ± 0.165	0.644 ± 0.488	0.651 ± 0.531	0.227 ± 0.689
	BN	-0.012 ± 0.024	2.117 ± 0.886	0.924 ± 0.301	0.961 ± 0.357	0.748 ± 0.113
	NonPar	0 ± 0.025	0.71 ± 0.196	0.621 ± 0.514	0.634 ± 0.568	0.154 ± 0.675
	PV	-0.013 ± 0.024	1.864 ± 1.109	0.838 ± 0.22	0.857 ± 0.252	0.742 ± 0.111
	Semi.PV	0.056 ± 0.135	1.028 ± 5.926	0.529 ± 1.599	0.526 ± 1.371	0.356 ± 1.228
Skewed III	Emp	0 ± 0.024	0.002 ± 0.006	0.03 ± 0.017	0.029 ± 0.015	0.018 ± 0.033
	BN	0.013 ± 0.022	0.059 ± 0.01	0.045 ± 0.006	0.048 ± 0.008	0.034 ± 0.004
	NonPar	0 ± 0.024	0.002 ± 0.006	0.03 ± 0.018	0.029 ± 0.015	0.017 ± 0.033
	PV	0.013 ± 0.022	0.056 ± 0.009	0.043 ± 0.005	0.046 ± 0.006	0.034 ± 0.004
	Semi.PV	0.039 ± 0.112	0.028 ± 0.14	0.035 ± 0.048	0.032 ± 0.044	0.031 ± 0.042
Mixed I	Emp	0.001 ± 0.026	-1.096 ± 1.605	-0.328 ± 0.326	-0.519 ± 0.641	0.013 ± 0.1
	BN	0 ± 0.024	0.001 ± 0.082	0.001 ± 0.057	-0.001 ± 0.064	0.001 ± 0.071
	NonPar	0.001 ± 0.026	-0.651 ± 1.727	-0.272 ± 0.369	-0.408 ± 0.683	0.007 ± 0.099
	PV	0 ± 0.024	-0.001 ± 0.081	0 ± 0.057	-0.003 ± 0.063	0 ± 0.07
	Semi.PV	-0.004 ± 0.034	-0.047 ± 0.113	-0.042 ± 0.088	-0.064 ± 0.115	-0.007 ± 0.097
Mixed II	Emp	-0.017 ± 0.024	-0.779 ± 0.552	-0.147 ± 0.112	-0.196 ± 0.114	-0.083 ± 0.066
	BN	-0.017 ± 0.024	-0.003 ± 0.127	0 ± 0.06	0 ± 0.067	-0.049 ± 0.066
	NonPar	-0.017 ± 0.024	-0.771 ± 0.698	-0.143 ± 0.12	-0.192 ± 0.133	-0.083 ± 0.067
	PV	-0.018 ± 0.024	-0.002 ± 0.127	0 ± 0.061	0 ± 0.067	-0.049 ± 0.066
	Semi.PV	-0.018 ± 0.024	-0.004 ± 0.132	-0.003 ± 0.063	-0.004 ± 0.069	-0.05 ± 0.066

© XXXX WILEY-VCH Verlag GmbH & Co. KGaA, Weinheim

Table B.4b Biases of estimating AUC and optimal cutoffs for different fitting models and medium AUC level for high sample size.

**Data generating mechanism**	**Fitting model**	**Median ± IQR**				
		AUC	J	ER	CZ	IU
BN equal	Emp	0.001 ± 0.02	0.004 ± 0.057	0 ± 0.055	0.002 ± 0.057	0.004 ± 0.053
	BN	0 ± 0.02	0.001 ± 0.063	0 ± 0.044	0 ± 0.047	0.001 ± 0.063
	NonPar	0 ± 0.02	0.003 ± 0.062	0.003 ± 0.057	0.003 ± 0.06	0.003 ± 0.056
	PV	0 ± 0.02	0.001 ± 0.063	0.001 ± 0.043	0 ± 0.047	0.001 ± 0.063
	Semi.PV	-0.002 ± 0.021	0 ± 0.065	-0.002 ± 0.045	-0.001 ± 0.048	0 ± 0.065
BN unequal	Emp	0 ± 0.021	0.16 ± 0.136	-0.042 ± 0.051	0.057 ± 0.061	-0.029 ± 0.17
	BN	0 ± 0.02	0 ± 0.036	0 ± 0.03	0 ± 0.031	0.002 ± 0.04
	NonPar	-0.001 ± 0.021	0.158 ± 0.154	-0.042 ± 0.052	0.057 ± 0.061	-0.015 ± 0.163
	PV	0 ± 0.02	0 ± 0.036	0 ± 0.03	0 ± 0.031	0.002 ± 0.04
	Semi.PV	-0.002 ± 0.022	-0.001 ± 0.037	-0.002 ± 0.032	-0.001 ± 0.033	-0.002 ± 0.042
Skewed I	Emp	-0.17 ± 0.025	1.103 ± 0.126	0.957 ± 0.297	1.012 ± 0.245	0.954 ± 0.534
	BN	-0.201 ± 0.026	1.521 ± 0.374	1.041 ± 0.082	1.114 ± 0.103	1.125 ± 0.085
	NonPar	-0.17 ± 0.025	1.096 ± 0.25	0.947 ± 0.314	1.004 ± 0.27	0.8 ± 0.511
	PV	-0.201 ± 0.026	1.519 ± 0.372	1.041 ± 0.083	1.114 ± 0.103	1.125 ± 0.085
	Semi.PV	-0.182 ± 0.122	0.99 ± 1.944	0.896 ± 0.82	0.894 ± 0.763	1.063 ± 0.724
Skewed II	Emp	0.001 ± 0.019	1.059 ± 0.585	0.776 ± 0.728	0.885 ± 0.74	0.55 ± 0.739
	BN	-0.092 ± 0.029	1.72 ± 0.339	0.971 ± 0.136	1.178 ± 0.183	1.164 ± 0.178
	NonPar	0 ± 0.019	1.041 ± 0.702	0.617 ± 0.795	0.806 ± 0.799	0.529 ± 0.78
	PV	-0.088 ± 0.03	1.637 ± 0.28	0.951 ± 0.123	1.136 ± 0.153	1.179 ± 0.173
	Semi.PV	-0.08 ± 0.149	0.454 ± 1.367	0.626 ± 1.038	0.569 ± 1.102	0.891 ± 0.885
Skewed III	Emp	0 ± 0.02	0.063 ± 0.039	0.101 ± 0.073	0.089 ± 0.066	0.064 ± 0.114
	BN	-0.033 ± 0.015	0.078 ± 0.015	0.07 ± 0.01	0.092 ± 0.013	0.024 ± 0.008
	NonPar	0 ± 0.02	0.062 ± 0.051	0.099 ± 0.081	0.088 ± 0.069	0.047 ± 0.113
	PV	0.018 ± 0.017	0.051 ± 0.014	0.056 ± 0.009	0.066 ± 0.011	0.035 ± 0.008
	Semi.PV	0.019 ± 0.192	0.05 ± 0.092	0.049 ± 0.058	0.045 ± 0.075	0.04 ± 0.05
Mixed I	Emp	0.001 ± 0.022	-0.35 ± 0.309	0.081 ± 0.11	-0.128 ± 0.134	0.07 ± 0.359
	BN	0 ± 0.02	0.002 ± 0.07	0.003 ± 0.062	0.001 ± 0.061	0.004 ± 0.081
	NonPar	0.001 ± 0.022	-0.34 ± 0.347	0.079 ± 0.11	-0.127 ± 0.143	0.041 ± 0.349
	PV	0 ± 0.02	-0.003 ± 0.07	0.001 ± 0.062	-0.004 ± 0.062	0.001 ± 0.08
	Semi.PV	-0.014 ± 0.059	-0.196 ± 0.375	-0.129 ± 0.239	-0.213 ± 0.339	-0.043 ± 0.211
Mixed II	Emp	-0.037 ± 0.022	-0.391 ± 0.13	-0.057 ± 0.081	-0.128 ± 0.092	-0.2 ± 0.072
	BN	-0.037 ± 0.022	0.001 ± 0.09	0 ± 0.057	0 ± 0.061	-0.122 ± 0.069
	NonPar	-0.037 ± 0.022	-0.381 ± 0.254	-0.055 ± 0.084	-0.122 ± 0.111	-0.2 ± 0.075
	PV	-0.038 ± 0.022	0.001 ± 0.09	0.001 ± 0.057	0 ± 0.061	-0.123 ± 0.068
	Semi.PV	-0.04 ± 0.023	-0.002 ± 0.092	-0.002 ± 0.059	-0.003 ± 0.065	-0.126 ± 0.07

© XXXX WILEY-VCH Verlag GmbH & Co. KGaA, Weinheim

Table B.4c Biases of estimating AUC and optimal cutoffs for different fitting models and high AUC level for high sample size.

Data generating mechanism	Fitting model	Median ± IQR				
		AUC	J	ER	CZ	IU
BN equal	Emp	0 ± 0.007	0.002 ± 0.054	0.001 ± 0.053	0.002 ± 0.054	0.002 ± 0.054
	BN	0 ± 0.007	0.003 ± 0.046	0.002 ± 0.052	0.002 ± 0.047	0.003 ± 0.046
	NonPar	0 ± 0.007	0.002 ± 0.055	0 ± 0.053	0.001 ± 0.056	0.002 ± 0.055
	PV	-0.002 ± 0.007	0.003 ± 0.046	0.002 ± 0.052	0.002 ± 0.048	0.003 ± 0.046
	Semi.PV	-0.004 ± 0.008	-0.001 ± 0.05	-0.003 ± 0.058	-0.001 ± 0.052	-0.001 ± 0.05
BN unequal	Emp	0 ± 0.012	0.136 ± 0.058	0.267 ± 0.225	0.166 ± 0.079	0.191 ± 0.263
	BN	0 ± 0.011	0.001 ± 0.034	0.001 ± 0.036	0.001 ± 0.034	0.002 ± 0.05
	NonPar	0 ± 0.012	0.134 ± 0.065	0.263 ± 0.237	0.162 ± 0.099	0.183 ± 0.266
	PV	0.001 ± 0.011	-0.004 ± 0.036	0.004 ± 0.036	-0.002 ± 0.035	0.005 ± 0.046
	Semi.PV	-0.01 ± 0.023	-0.102 ± 0.221	-0.071 ± 0.135	-0.103 ± 0.207	-0.089 ± 0.125
Skewed I	Emp	0 ± 0.014	-5.652 ± 0.638	-4.757 ± 0.508	-4.878 ± 0.538	-3.42 ± 0.527
	BN	-0.011 ± 0.018	-5.604 ± 0.107	-4.714 ± 0.149	-4.823 ± 0.117	-3.372 ± 0.107
	NonPar	-0.001 ± 0.013	-5.658 ± 0.647	-4.763 ± 0.513	-4.885 ± 0.554	-3.426 ± 0.552
	PV	-0.012 ± 0.018	-5.603 ± 0.107	-4.713 ± 0.149	-4.822 ± 0.117	-3.371 ± 0.107
	Semi.PV	-0.012 ± 0.049	-5.423 ± 1.108	-4.145 ± 1.032	-4.527 ± 1.058	-2.873 ± 1.102
Skewed II	Emp	0.001 ± 0.013	2.919 ± 1.806	2.071 ± 1.857	2.622 ± 1.743	2.684 ± 1.76
	BN	-0.067 ± 0.03	3.54 ± 0.435	2.16 ± 0.465	2.995 ± 0.343	3.272 ± 0.436
	NonPar	0 ± 0.014	2.809 ± 2.322	1.323 ± 2.144	1.877 ± 2.319	2.574 ± 1.865
	PV	-0.066 ± 0.031	3.486 ± 0.397	2.16 ± 0.465	2.968 ± 0.335	3.244 ± 0.394
	Semi.PV	-0.044 ± 0.075	2.227 ± 2.735	2.263 ± 2.546	2.255 ± 2.558	3.05 ± 2.626
Skewed III	Emp	0.001 ± 0.012	1.461 ± 0.9	1.528 ± 0.9	1.478 ± 0.9	1.519 ± 0.905
	BN	-0.166 ± 0.016	0.057 ± 0.024	0.051 ± 0.017	0.066 ± 0.023	-0.055 ± 0.01
	NonPar	0.001 ± 0.012	1.424 ± 1.087	1.491 ± 1.102	1.441 ± 1.102	1.483 ± 1.109
	PV	0.04 ± 0.01	1.559 ± 0.154	1.626 ± 0.154	1.576 ± 0.154	1.618 ± 0.154
	Semi.PV	0.036 ± 0.08	1.526 ± 0.874	1.531 ± 0.837	1.511 ± 0.829	1.156 ± 1.285
Mixed I	Emp	0 ± 0.012	0.33 ± 0.121	0.61 ± 0.467	0.389 ± 0.178	0.492 ± 0.551
	BN	0 ± 0.011	0.003 ± 0.072	0.003 ± 0.073	0.003 ± 0.07	0.003 ± 0.101
	NonPar	0 ± 0.012	0.325 ± 0.142	0.602 ± 0.488	0.384 ± 0.246	0.477 ± 0.561
	PV	0.002 ± 0.011	-0.013 ± 0.074	0.009 ± 0.073	-0.009 ± 0.072	0.021 ± 0.095
	Semi.PV	-0.01 ± 0.023	-0.232 ± 0.455	-0.18 ± 0.258	-0.244 ± 0.401	-0.2 ± 0.269
Mixed II	Emp	-0.039 ± 0.013	-0.016 ± 0.076	0.103 ± 0.083	0.03 ± 0.084	-0.017 ± 0.076
	BN	-0.039 ± 0.012	0.001 ± 0.056	0.003 ± 0.061	0.003 ± 0.057	0.001 ± 0.056
	NonPar	-0.039 ± 0.013	-0.015 ± 0.081	0.102 ± 0.092	0.028 ± 0.086	-0.017 ± 0.078
	PV	-0.041 ± 0.012	0.001 ± 0.056	0.003 ± 0.061	0.003 ± 0.057	0.001 ± 0.056
	Semi.PV	-0.043 ± 0.016	-0.01 ± 0.061	-0.003 ± 0.07	-0.007 ± 0.065	-0.011 ± 0.062

© XXXX WILEY-VCH Verlag GmbH & Co. KGaA, Weinheim

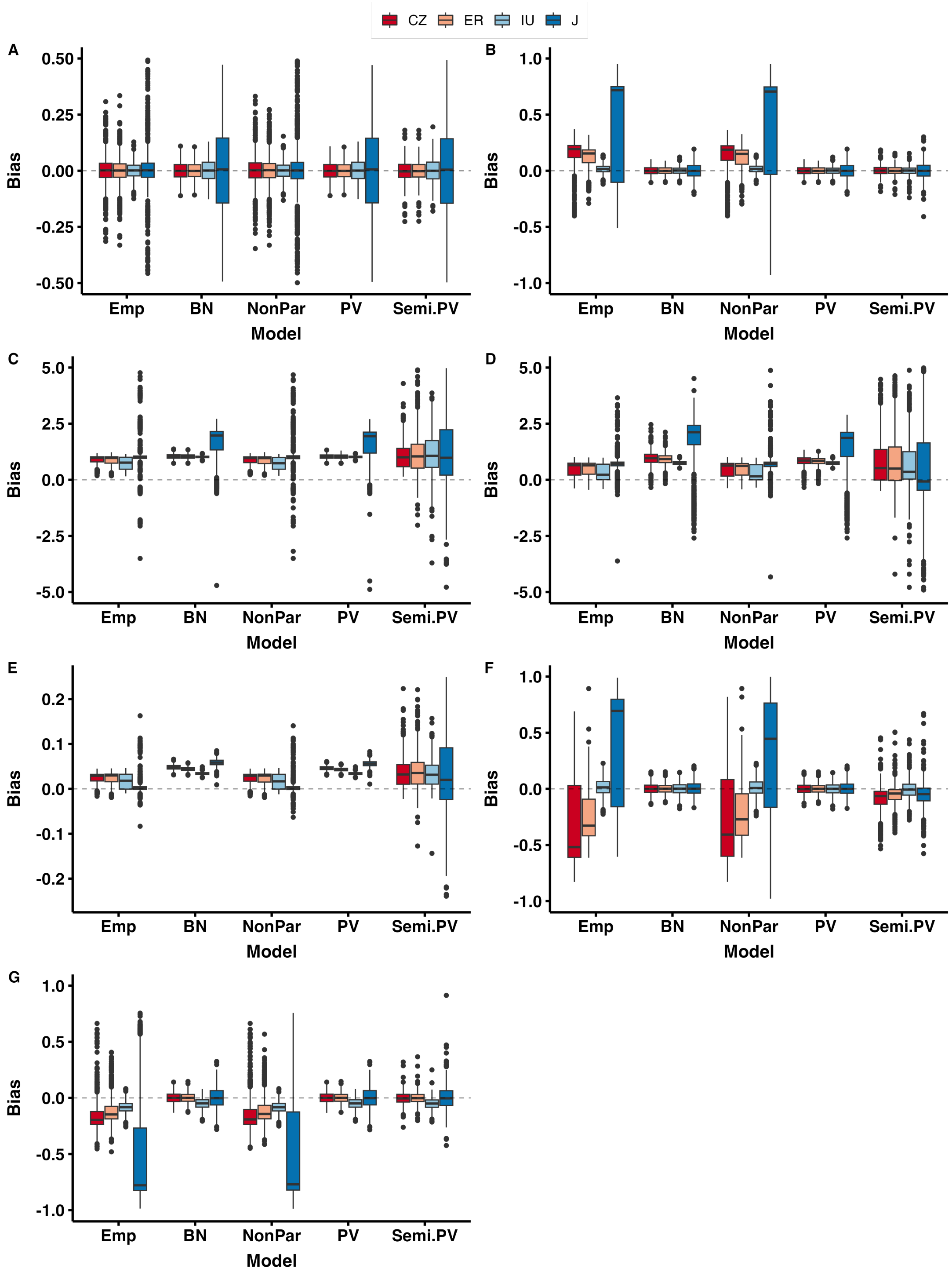

Figure B.13 Bias in estimation of optimal cutoff for high sample size, when AUC level is low. Panels A - G respectively correspond to the simulation scenarios: BN equal, BN unequal, Skewed I, Skewed II, Skewed III, Mixed I, and Mixed II.

© XXXX WILEY-VCH Verlag GmbH & Co. KGaA, Weinheim

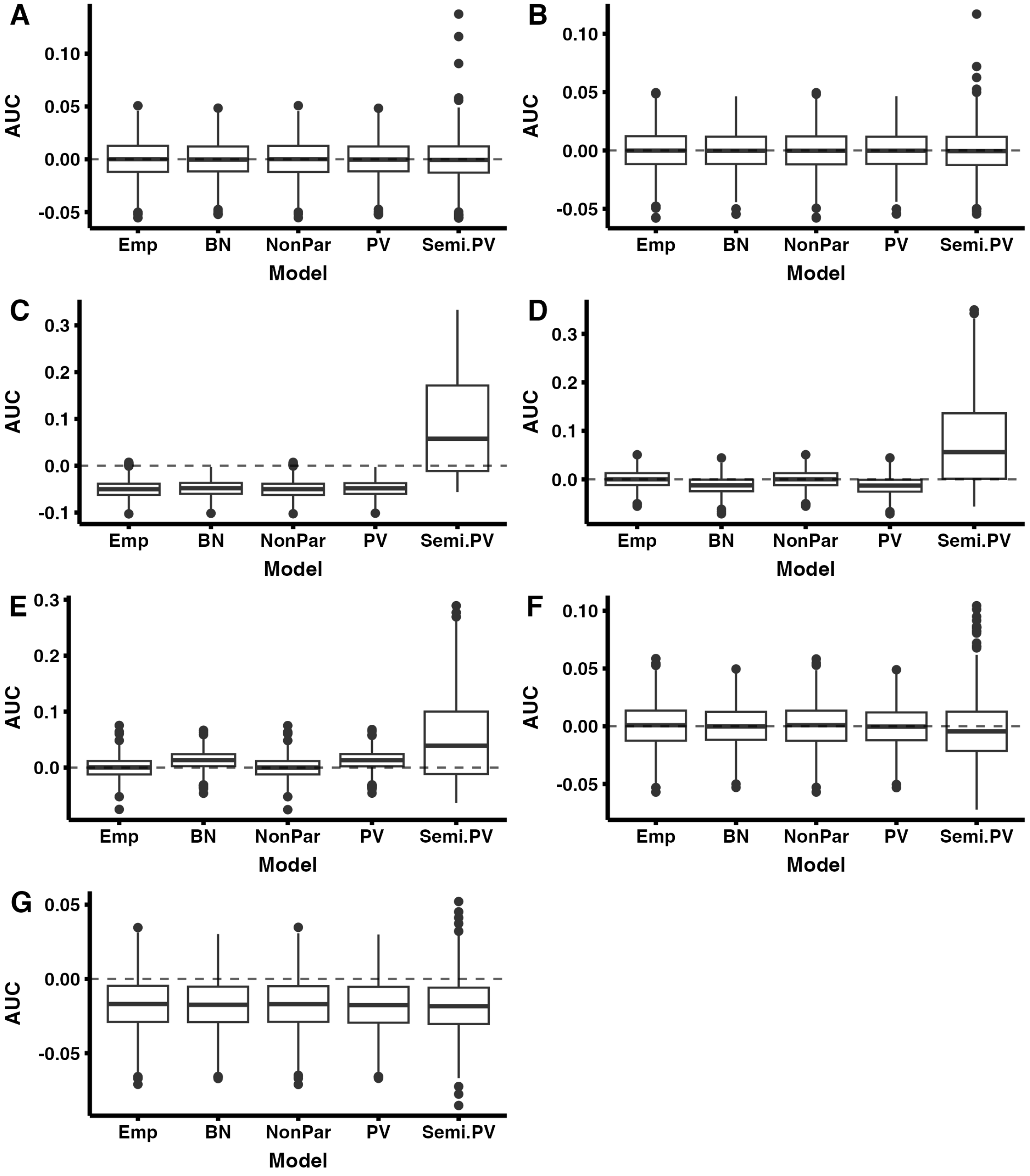

Figure B.14 Bias in estimation of AUC for high sample size, when AUC level is low. Panels A - G respectively correspond to the simulation scenarios: BN equal, BN unequal, Skewed I, Skewed II, Skewed III, Mixed I, and Mixed II.

© XXXX WILEY-VCH Verlag GmbH & Co. KGaA, Weinheim

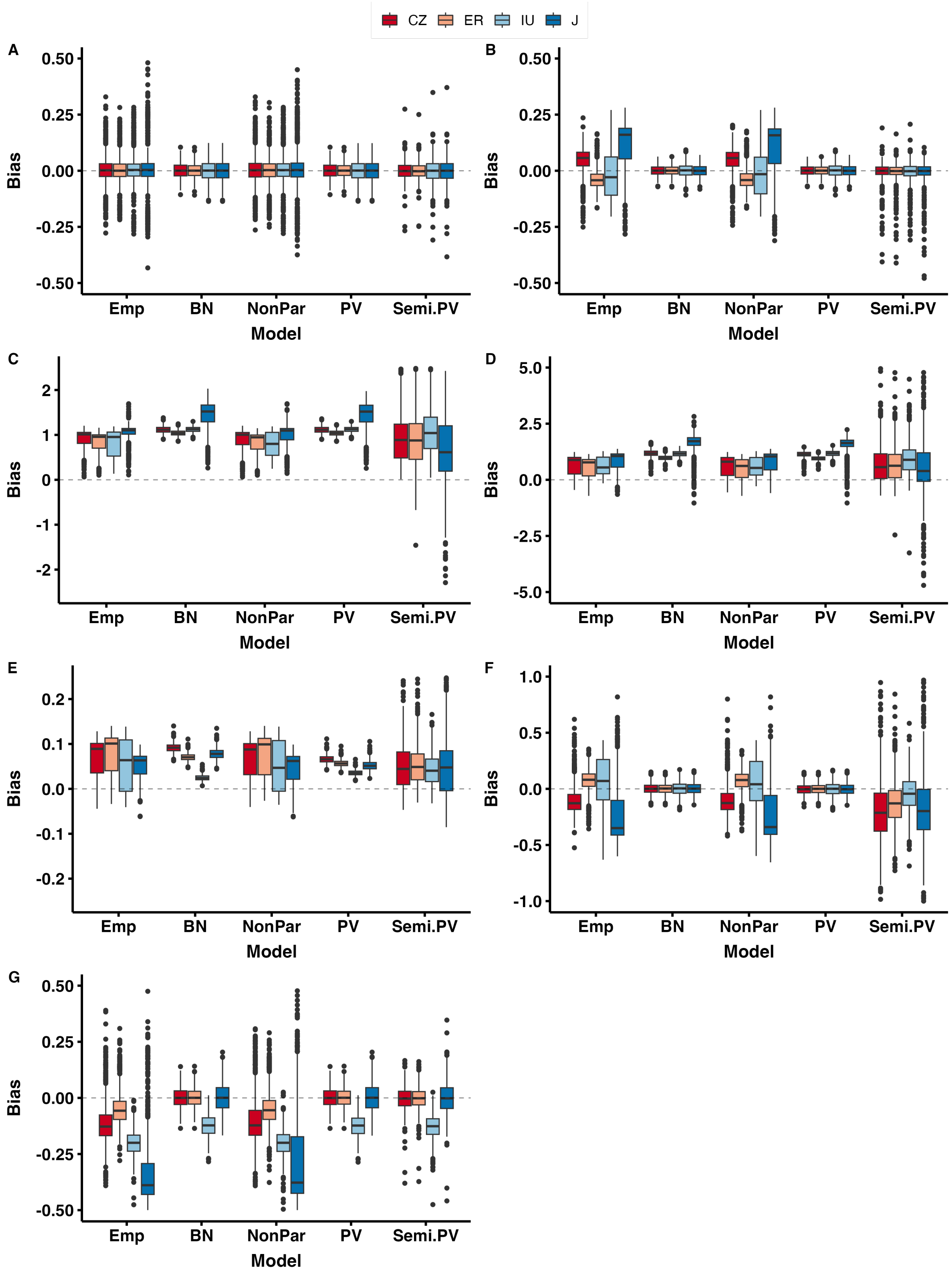

Figure B.15 Bias in estimation of optimal cutoff for high sample size, when AUC level is medium. Panels A - G respectively correspond to the simulation scenarios: BN equal, BN unequal, Skewed I, Skewed II, Skewed III, Mixed I, and Mixed II.

© XXXX WILEY-VCH Verlag GmbH & Co. KGaA, Weinheim

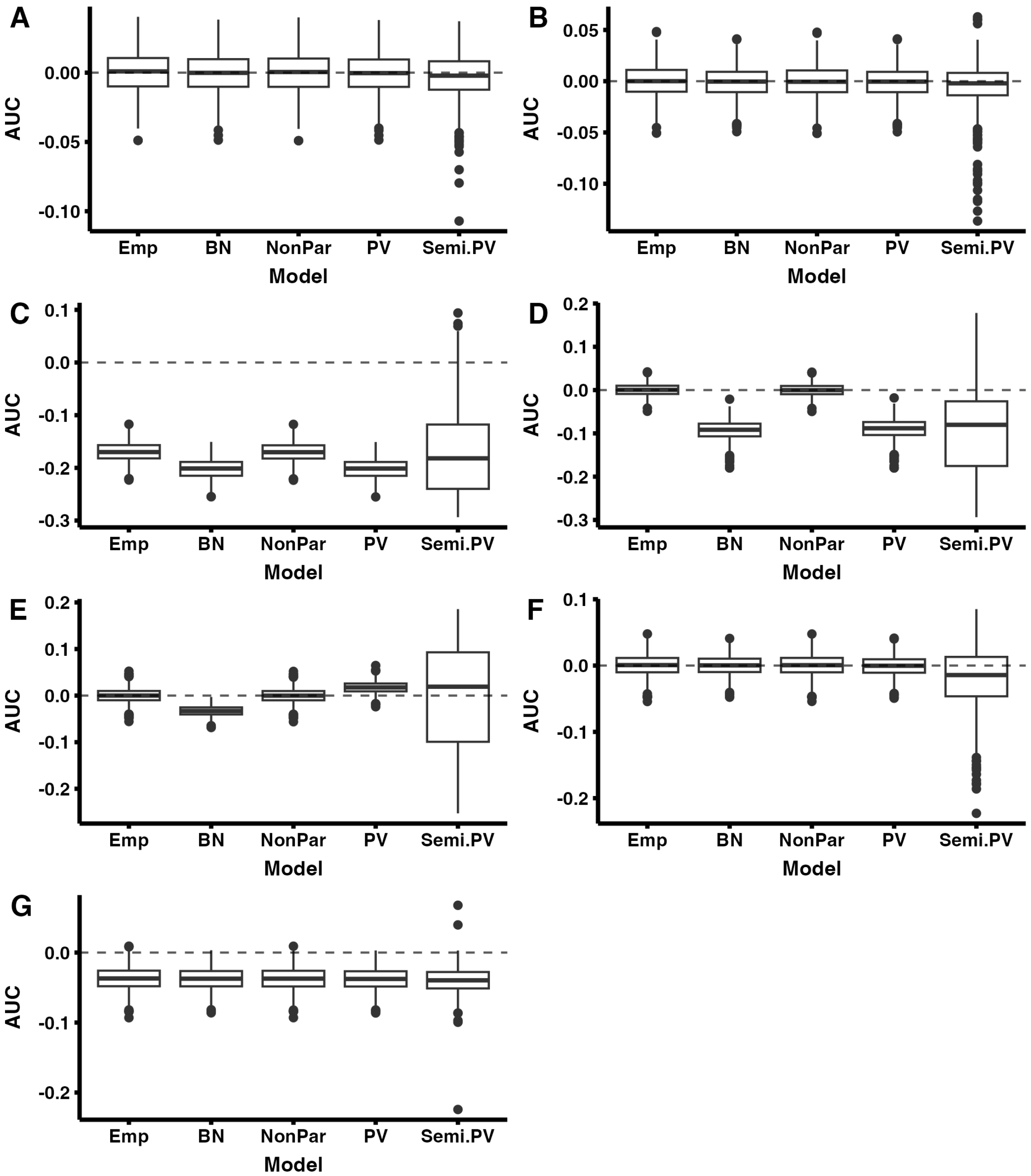

Figure B.16 Bias in estimation of AUC for high sample size, when AUC level is medium. Panels A - G respectively correspond to the simulation scenarios: BN equal, BN unequal, Skewed I, Skewed II, Skewed III, Mixed I, and Mixed II.

© XXXX WILEY-VCH Verlag GmbH & Co. KGaA, Weinheim

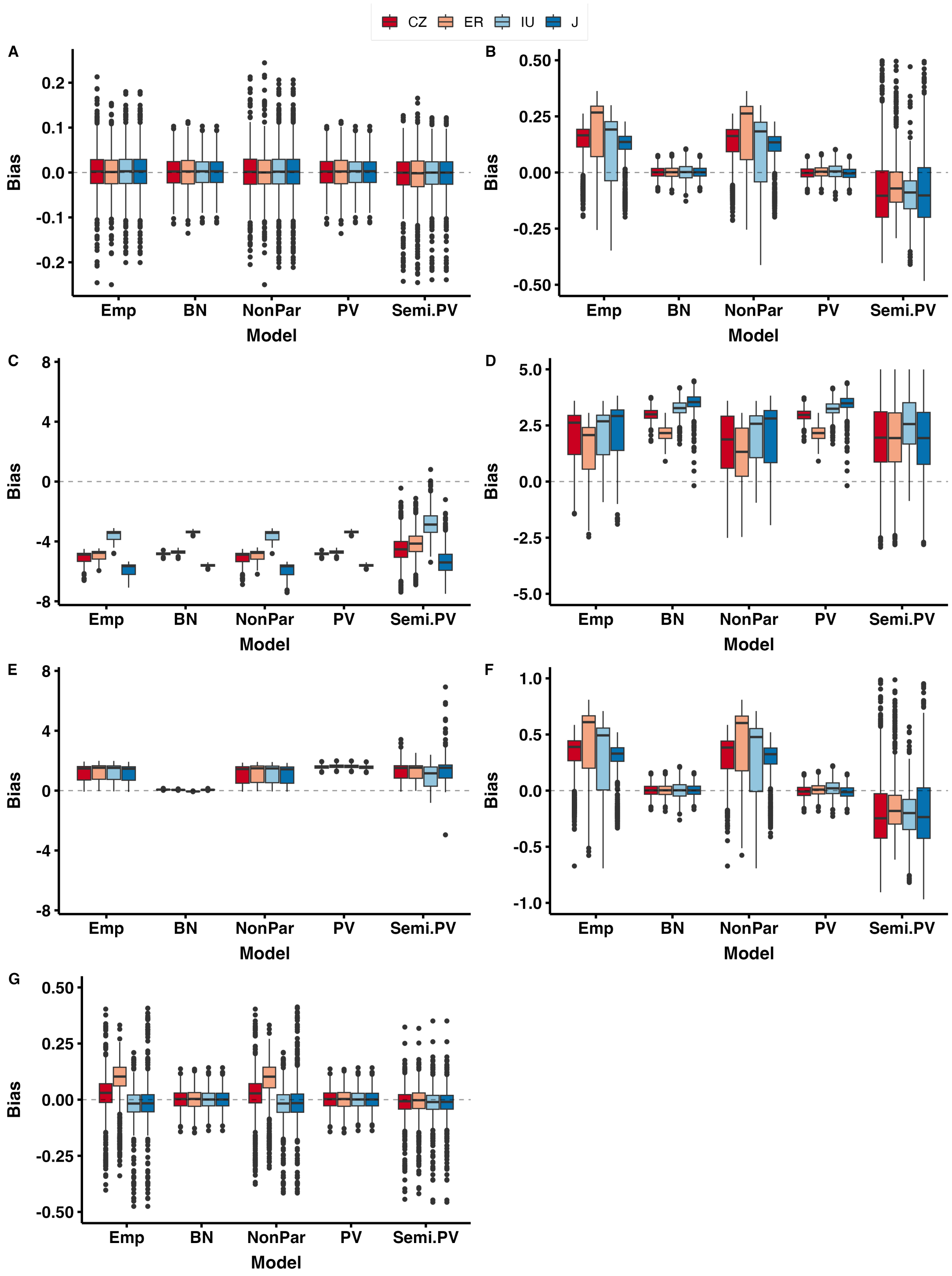

Figure B.17 Bias in estimation of optimal cutoff for high sample size, when AUC level is high. Panels A - G respectively correspond to the simulation scenarios: BN equal, BN unequal, Skewed I, Skewed II, Skewed III, Mixed I, and Mixed II.

© XXXX WILEY-VCH Verlag GmbH & Co. KGaA, Weinheim

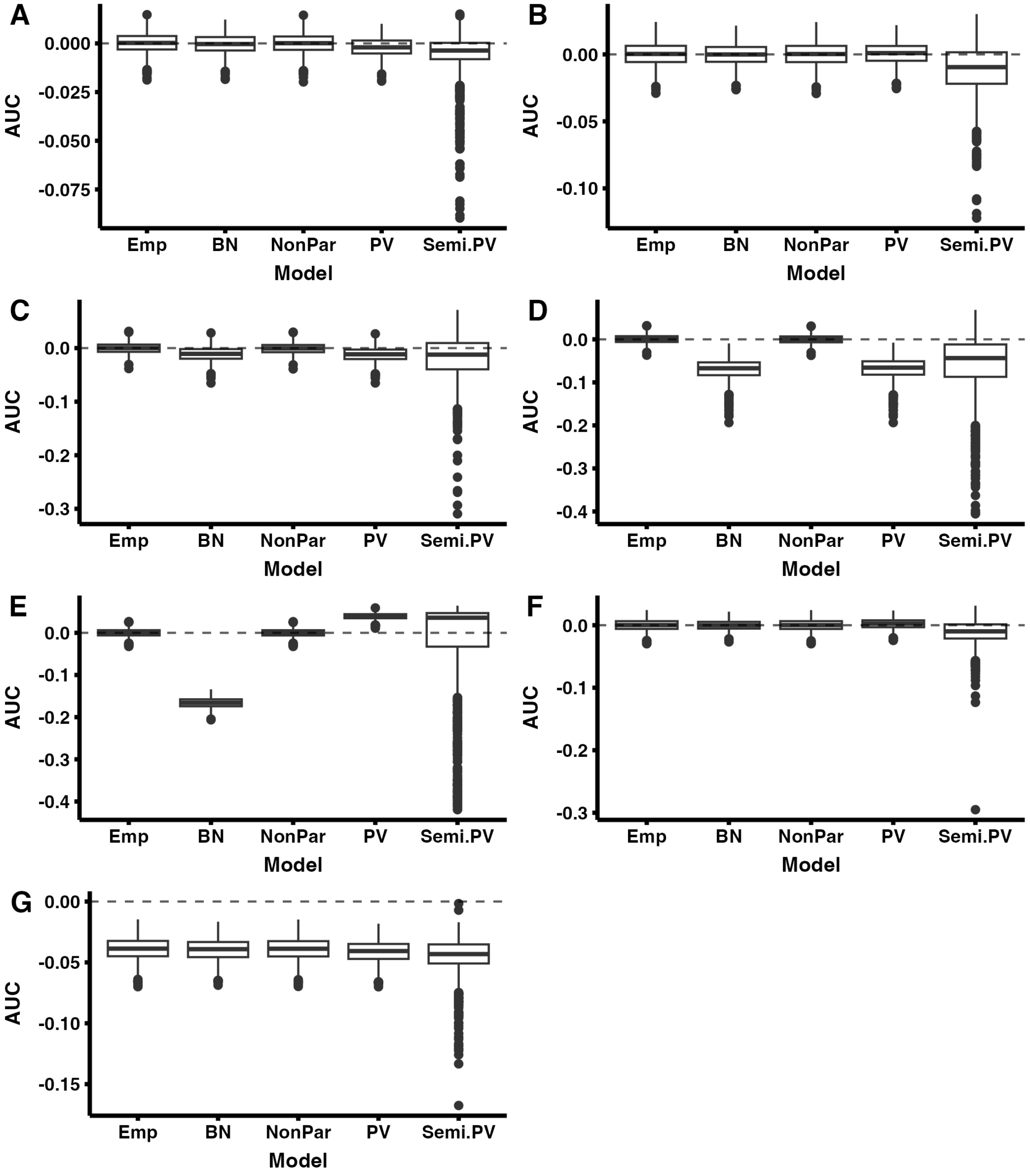

Figure B.18 Bias in estimation of AUC for high sample size, when AUC level is high. Panels A - G respectively correspond to the simulation scenarios: BN equal, BN unequal, Skewed I, Skewed II, Skewed III, Mixed I, and Mixed II.

© XXXX WILEY-VCH Verlag GmbH & Co. KGaA, Weinheim

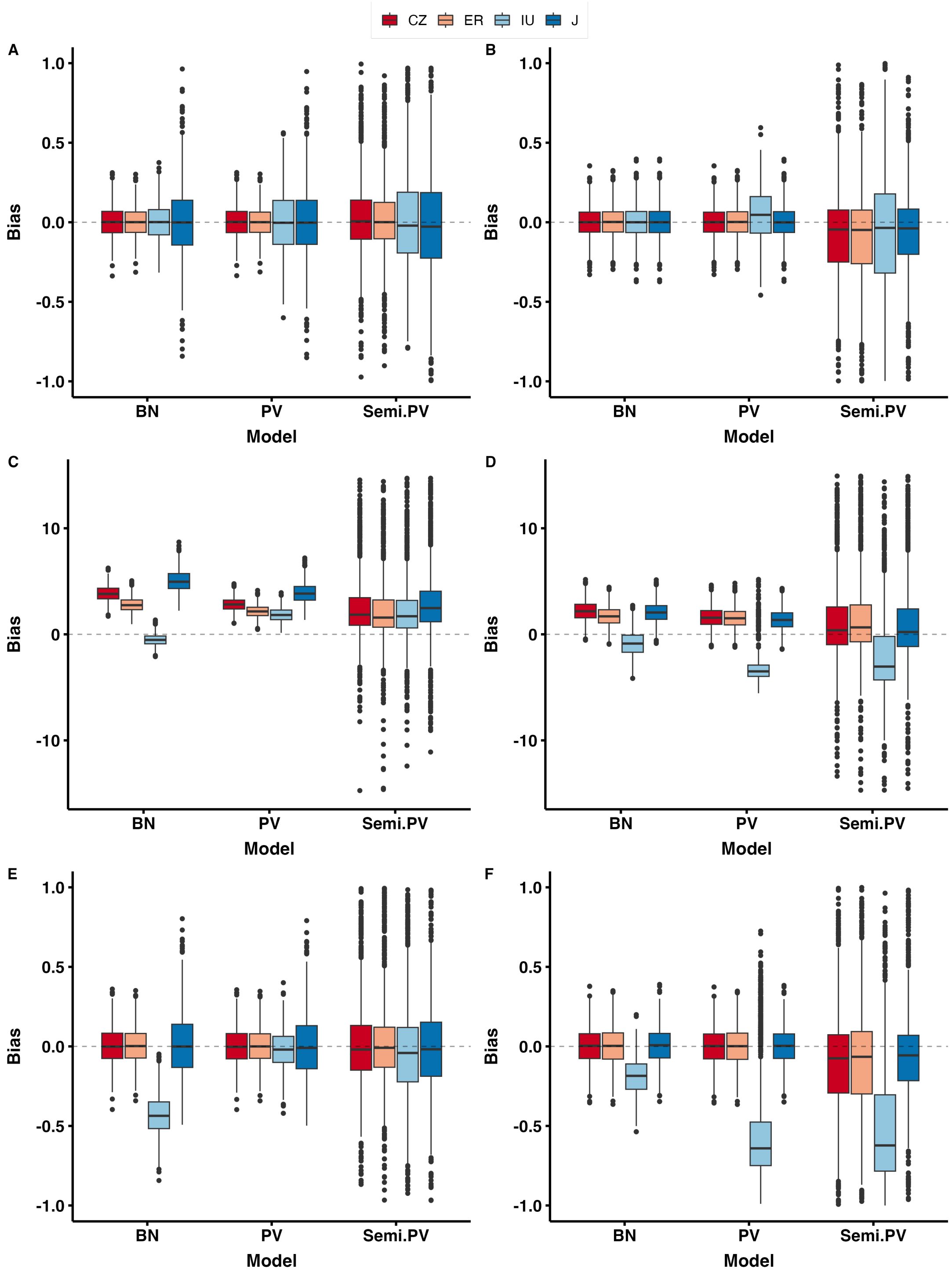

Figure B.19 Bias in estimation of optimal cutoff for medium sample size in the presence of covariates. The left panel (A, C, E) respectively corresponds to simulation scenarios: BN, Skewed, and Mixed for covariate value at 0. The right panel (B, D, F) corresponds to the covariate value at 1.

© XXXX WILEY-VCH Verlag GmbH & Co. KGaA, Weinheim

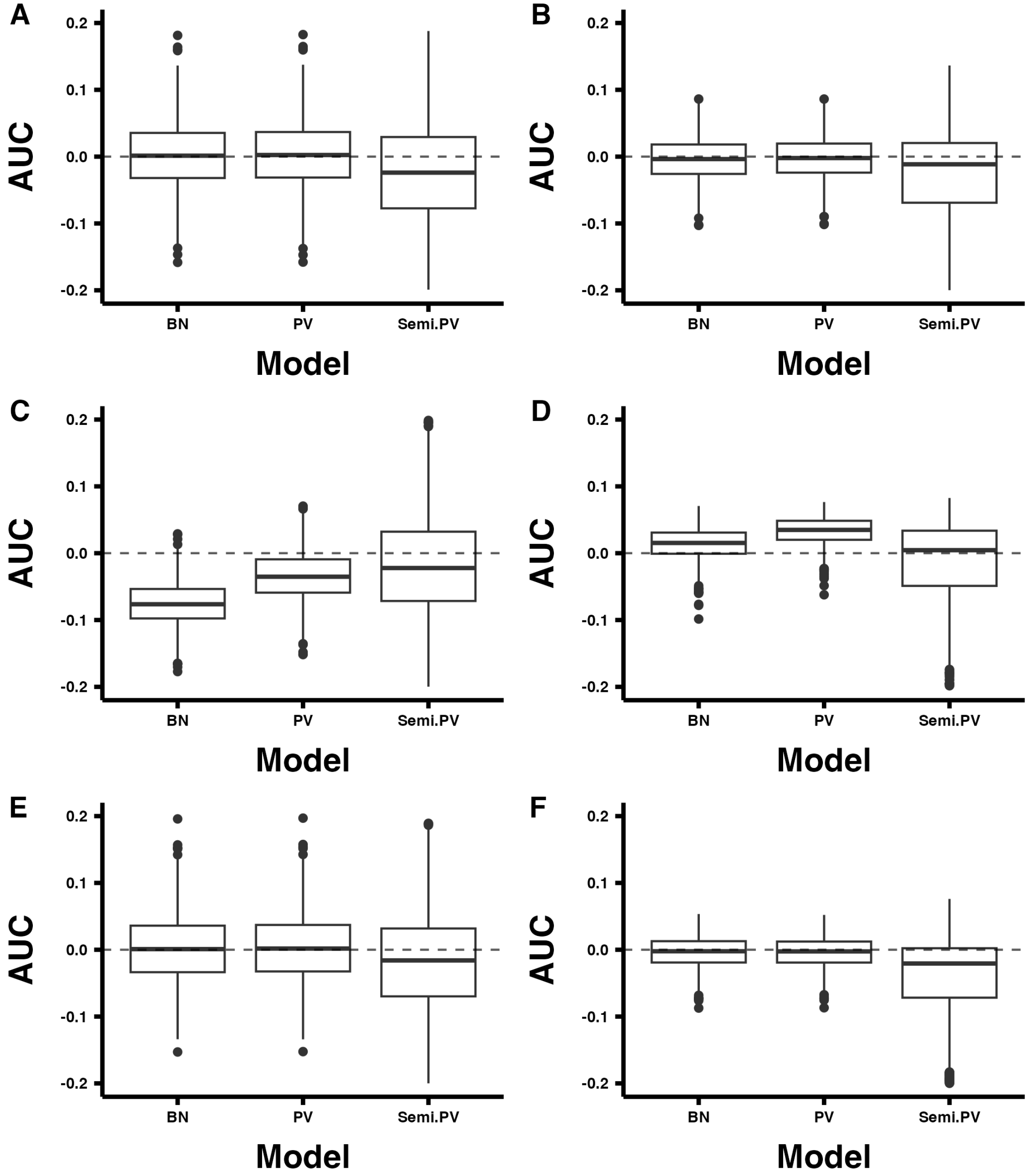

Figure B.20 Bias in estimation of AUC for medium sample size in the presence of covariates. The left panel (A, C, E) respectively corresponds to simulation scenarios: BN, Skewed, and Mixed for covariate value at 0. The right panel (B, D, F) corresponds to the covariate value at 1.

© XXXX WILEY-VCH Verlag GmbH & Co. KGaA, Weinheim

Table B.5 Biases of estimating of AUC and optimal cutoffs for different fitting models at different covariate levels for low sample size.

Data generating mechanism	Fitting model	Median ± IQR				
		AUC	J	ER	CZ	IU
**x = 0**						
BN	BN	-0.002 ± 0.099	0.001 ± 0.402	-0.007 ± 0.186	-0.002 ± 0.197	-0.001 ± 0.2
	PV	0 ± 0.101	0 ± 0.399	-0.006 ± 0.186	-0.002 ± 0.195	-0.006 ± 0.37
	Semi.PV	-0.051 ± 0.152	0.024 ± 0.798	-0.011 ± 0.305	-0.001 ± 0.318	-0.022 ± 0.437
Skewed	BN	-0.078 ± 0.059	4.987 ± 2.044	2.73 ± 1.343	3.794 ± 1.463	-0.586 ± 1.073
	PV	-0.036 ± 0.074	3.758 ± 1.991	2.062 ± 1.274	2.68 ± 1.342	1.699 ± 1.357
	Semi.PV	-0.053 ± 0.149	2.808 ± 3.815	1.428 ± 2.378	1.697 ± 2.583	1.635 ± 2.76
Mixed	BN	-0.001 ± 0.1	0.024 ± 0.418	0.001 ± 0.223	-0.005 ± 0.235	-0.448 ± 0.225
	PV	0 ± 0.102	0.006 ± 0.415	-0.004 ± 0.221	-0.01 ± 0.234	-0.042 ± 0.231
	Semi.PV	-0.04 ± 0.148	0.061 ± 0.868	-0.011 ± 0.371	-0.04 ± 0.385	-0.078 ± 0.476
**x = 1**						
BN	BN	-0.002 ± 0.061	0.008 ± 0.19	0.007 ± 0.175	0.007 ± 0.177	0.009 ± 0.189
	PV	0.002 ± 0.06	0.006 ± 0.187	0.007 ± 0.175	0.007 ± 0.179	0.028 ± 0.31
	Semi.PV	-0.046 ± 0.179	-0.062 ± 0.364	-0.075 ± 0.387	-0.076 ± 0.375	-0.077 ± 0.6
Skewed	BN	0.013 ± 0.046	2.082 ± 1.865	1.642 ± 1.742	2.174 ± 1.815	-0.994 ± 2.068
	PV	0.036 ± 0.042	1.298 ± 1.827	1.459 ± 1.741	1.482 ± 1.794	-3.324 ± 1.703
	Semi.PV	-0.021 ± 0.126	0.255 ± 4.376	0.794 ± 4.579	0.538 ± 4.629	-2.97 ± 5.266
Mixed	BN	-0.002 ± 0.045	0.012 ± 0.21	0.003 ± 0.216	0.006 ± 0.21	-0.194 ± 0.213
	PV	0 ± 0.044	0.003 ± 0.211	0 ± 0.217	0.002 ± 0.209	-0.628 ± 0.426
	Semi.PV	-0.045 ± 0.153	-0.084 ± 0.419	-0.078 ± 0.491	-0.094 ± 0.476	-0.729 ± 0.607

© XXXX WILEY-VCH Verlag GmbH & Co. KGaA, Weinheim

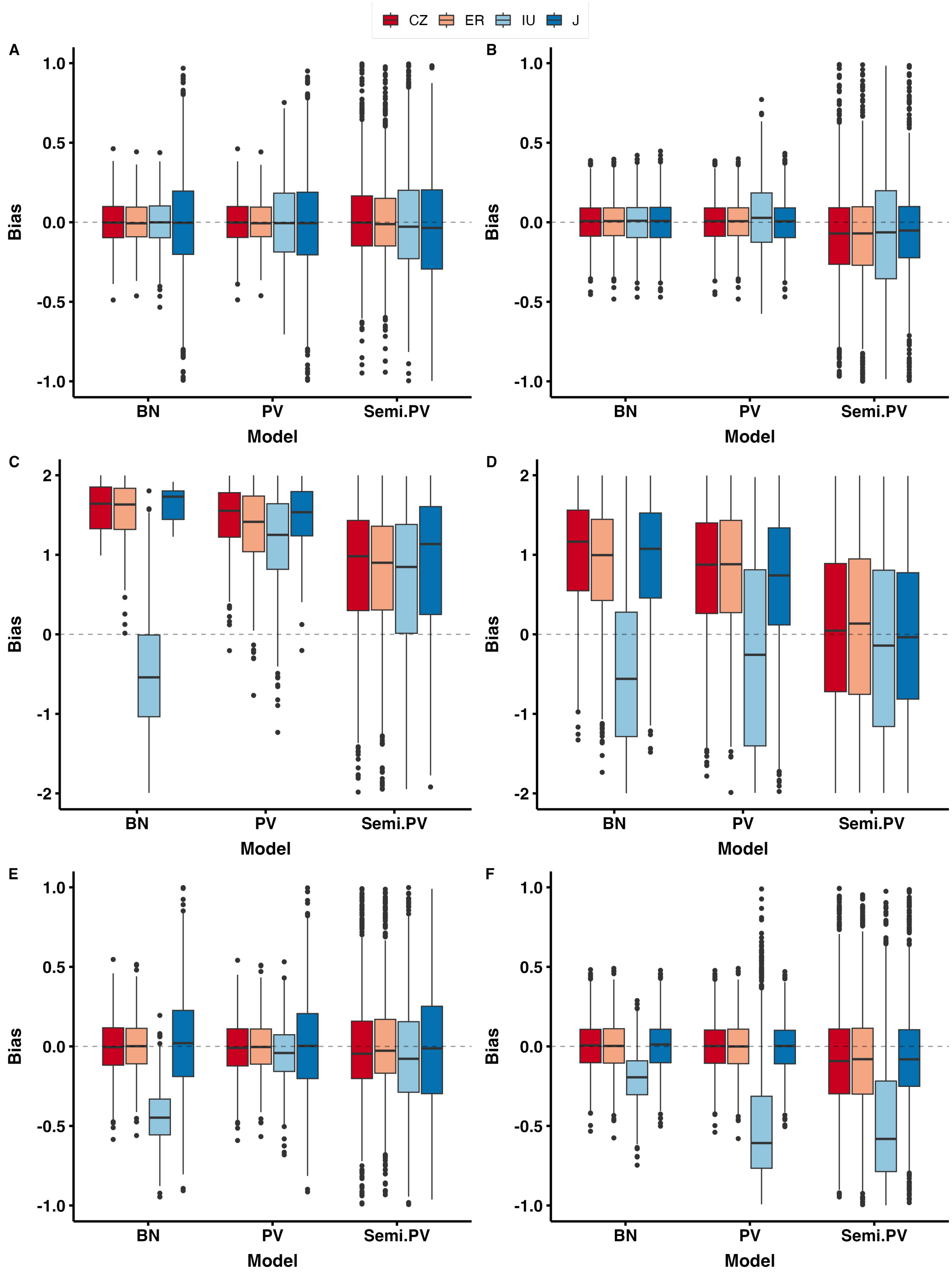

Figure B.21 Bias in estimation of AUC and optimal cutoff for low sample size in the presence of covariates. The left panel (A, C, E) respectively corresponds to simulation scenarios: BN, Skewed, and Mixed for covariate value at 0. The right panel (B, D, F) corresponds to the covariate value at 1.

© XXXX WILEY-VCH Verlag GmbH & Co. KGaA, Weinheim

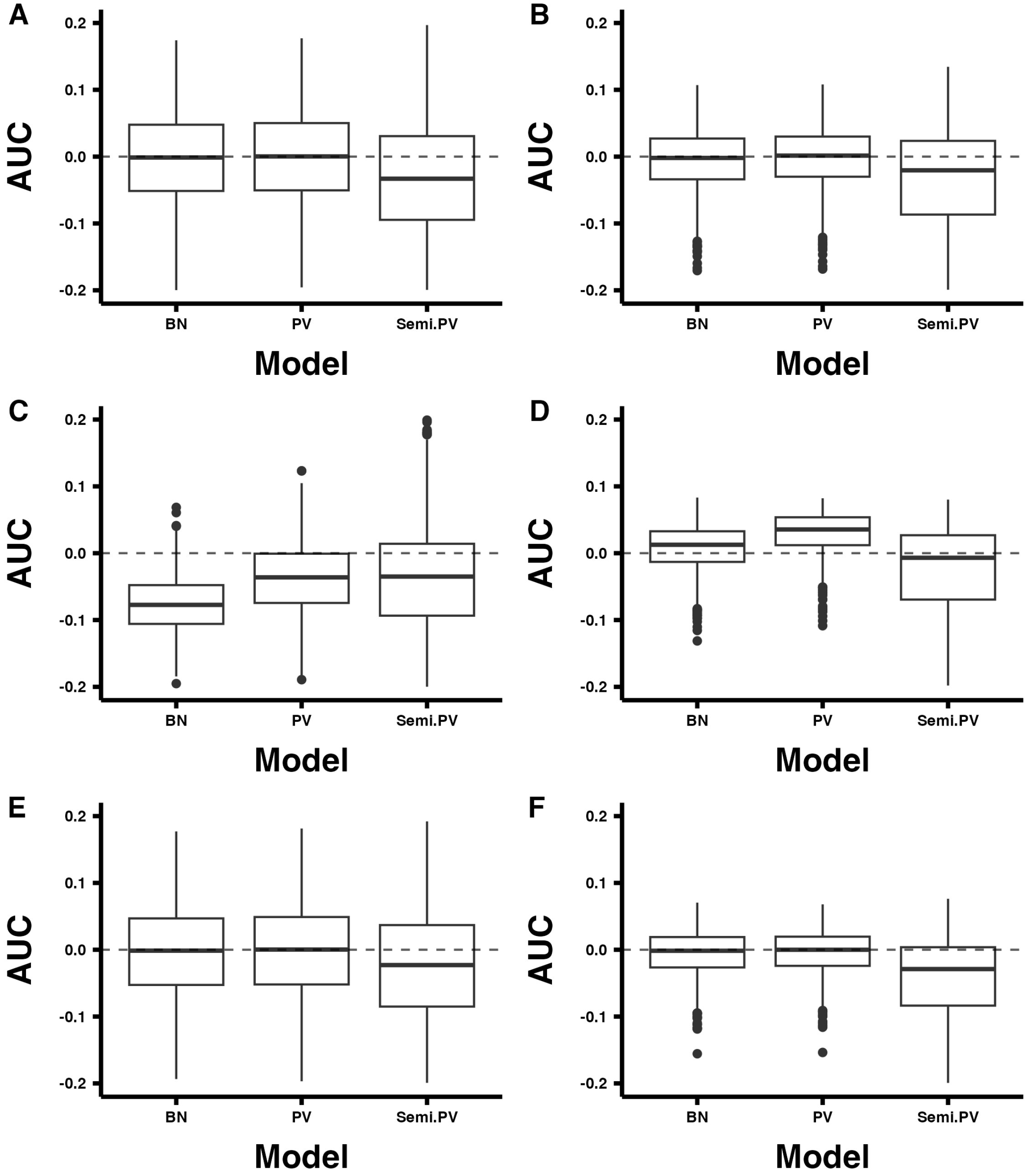

Figure B.22 Bias in estimation of AUC for low sample size in the presence of covariates. The left panel (A, C, E) respectively corresponds to simulation scenarios: BN, Skewed, and Mixed for covariate value at 0. The right panel (B, D, F) corresponds to the covariate value at 1.

© XXXX WILEY-VCH Verlag GmbH & Co. KGaA, Weinheim

Table B.6 Biases of estimating of AUC and optimal cutoffs for different fitting models at different covariate levels for high sample size.

Data generating mechanism	Fitting model	Median ± IQR				
		AUC	J	ER	CZ	IU
		**x = 0**				
BN	BN	0 ± 0.029	0 ± 0.126	-0.001 ± 0.065	-0.002 ± 0.069	0.001 ± 0.114
	PV	0 ± 0.029	0 ± 0.127	-0.002 ± 0.065	-0.002 ± 0.069	0 ± 0.127
	Semi.PV	-0.02 ± 0.118	0.024 ± 0.341	0.005 ± 0.15	0.005 ± 0.161	0 ± 0.233
Skewed	BN	-0.083 ± 0.018	5.178 ± 0.577	2.901 ± 0.387	4.023 ± 0.422	-0.564 ± 0.344
	PV	-0.043 ± 0.022	3.985 ± 0.585	2.211 ± 0.384	2.895 ± 0.405	1.813 ± 0.397
	Semi.PV	-0.015 ± 0.16	1.386 ± 7.347	0.761 ± 3.196	0.753 ± 3.443	0.447 ± 4.154
Mixed	BN	0 ± 0.032	-0.001 ± 0.122	-0.003 ± 0.075	-0.002 ± 0.079	-0.435 ± 0.08
	PV	0 ± 0.032	-0.003 ± 0.122	-0.003 ± 0.075	-0.003 ± 0.08	-0.001 ± 0.076
	Semi.PV	-0.018 ± 0.118	0.022 ± 0.431	-0.012 ± 0.187	-0.02 ± 0.202	-0.019 ± 0.278
		**x = 1**				
BN	BN	-0.001 ± 0.019	0.001 ± 0.058	0 ± 0.056	0.001 ± 0.056	0.001 ± 0.058
	PV	-0.001 ± 0.019	0.001 ± 0.057	0 ± 0.056	0.001 ± 0.056	0.071 ± 0.112
	Semi.PV	-0.019 ± 0.142	-0.017 ± 0.245	-0.026 ± 0.271	-0.026 ± 0.276	0.03 ± 0.443
Skewed	BN	0.005 ± 0.015	2.075 ± 0.609	1.59 ± 0.555	2.179 ± 0.581	-1.209 ± 0.707
	PV	0.031 ± 0.013	1.353 ± 0.608	1.461 ± 0.559	1.564 ± 0.583	-3.653 ± 0.469
	Semi.PV	0.001 ± 0.087	0.609 ± 4.827	1.274 ± 4.479	0.833 ± 4.69	-2.168 ± 4.012
Mixed	BN	0 ± 0.014	0.001 ± 0.068	0.001 ± 0.071	0.001 ± 0.067	-0.189 ± 0.068
	PV	-0.002 ± 0.014	0.001 ± 0.068	0.001 ± 0.071	0.001 ± 0.067	-0.695 ± 0.119
	Semi.PV	-0.019 ± 0.128	-0.053 ± 0.249	-0.048 ± 0.342	-0.055 ± 0.33	-0.741 ± 0.382

© XXXX WILEY-VCH Verlag GmbH & Co. KGaA, Weinheim

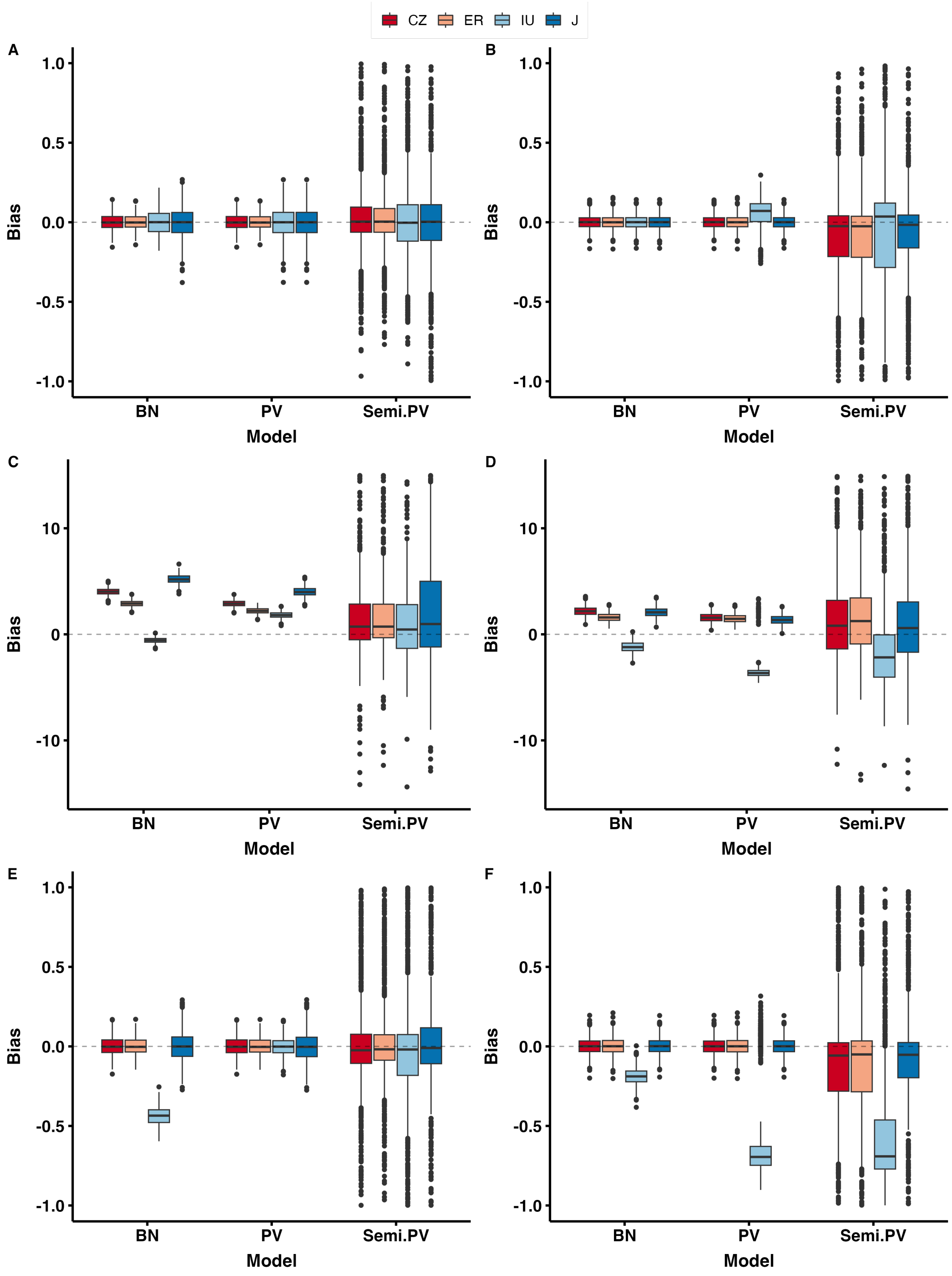

Figure B.23 Bias in estimation of AUC and optimal cutoff for high sample size in the presence of covariates. The left panel (A, C, E) respectively corresponds to simulation scenarios: BN, Skewed, and Mixed for covariate value at 0. The right panel (B, D, F) corresponds to the covariate value at 1.

© XXXX WILEY-VCH Verlag GmbH & Co. KGaA, Weinheim

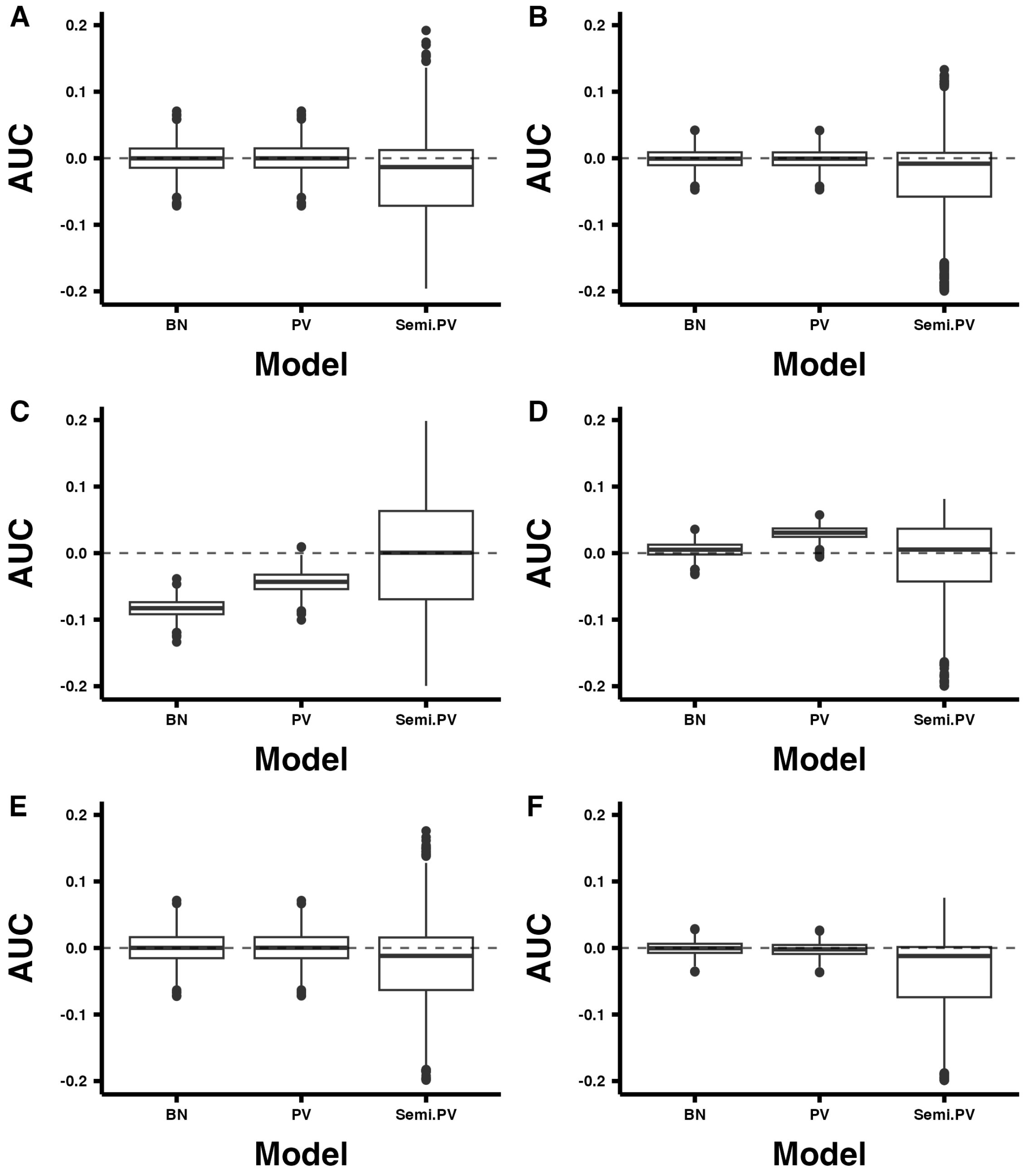

Figure B.24 Bias in estimation of AUC for high sample size in the presence of covariates. The left panel (A, C, E) respectively corresponds to simulation scenarios: BN, Skewed, and Mixed for covariate value at 0. The right panel (B, D, F) corresponds to the covariate value at 1.

© XXXX WILEY-VCH Verlag GmbH & Co. KGaA, Weinheim
